# Influence of intramolecular interactions and transition state energetics on the strain-promoted azide–alkyne cycloaddition of 2-aminobenzenesulfonamide-containing cyclononynes

**DOI:** 10.1039/d5ra06229b

**Published:** 2026-01-05

**Authors:** Kazuhide Nakahara, Kyosuke Kaneda

**Affiliations:** a Hokkaido University of Science, Faculty of Pharmaceutical Sciences 15-4-1, Maeda 7-jo Teine-ku Sapporo-shi Hokkaido 006-8585 Japan nakahara-k@hus.ac.jp

## Abstract

Computational investigation of the reaction mechanism and selectivity of strain-promoted azide–alkyne cycloaddition indicated that 2-aminobenzenesulfonamide-containing cyclononyne (ABSACN) is a HOMO donor; the energy difference is slight between the transition states (TSs) for the major and minor products. The reaction between unsubstituted ABSACN(R^1^=R^2^=H) and benzyl azide proceeds *via* the pathway that is most energetically favorable, minimizing the influence of steric repulsion, intermolecular interactions, and the strain energy of ABSACN. In the case of R^1^=Boc, the intramolecular hydrogen bond is maintained in both the X-ray-characterized minor product and its corresponding transition state, and the calculated product ratios coincided with the experimental data.

## Abstract 2

When a Boc-protected cyclononyne reacts, intramolecular hydrogen bonds are retained in the transition state. The calculated product ratios align with experimental findings.

## Graphical abstract

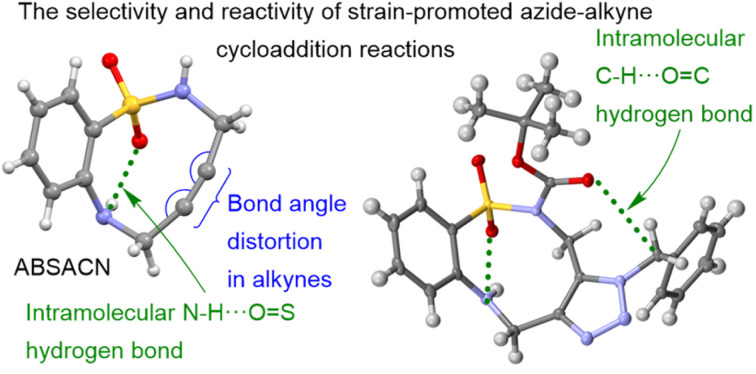

## Introduction

Click chemistry has been used for efficient, selective molecular ligation reactions across myriad fields, including medicinal chemistry (*e.g.*, drug conjugation) and materials science (*e.g.*, polymer modification).^1^ In particular, copper(i)-catalyzed azide–alkyne cycloaddition (Cu-AAC) has been widely used because of its high reactivity,^2^ as pioneered by Sharpless *et al.* However, because CuAAC uses a copper catalyst, toxicity concerns regarding its use in biological samples^3^ limit its application in bioorthogonal chemistry. Therefore, Bertozzi *et al.*^4^ developed strain-promoted azide–alkyne cycloaddition (SPAAC), which proceeds without a copper catalyst. SPAAC has attracted attention as a bioorthogonal reaction, including biocompatible reactions for live-cell imaging and drug delivery, which are extensively used in biomedicine and materials science. The reactivity of SPAAC is highly dependent on the degree of strain in the alkyne; thus, developing highly reactive alkynes is key to expanding the application range of SPAAC.^5^ This pursuit has led to the development of a diverse range of cyclooctyne-based reagents, each presenting a unique balance between reactivity and stability. For instance, difluorinated cyclooctynes (DIFO)^6^ achieve exceptionally high reaction rates due to significant ring strain, but their stability can be a practical concern for specific applications. In contrast, bicyclo[6.1.0]nonyne (BCN)^7^ offers excellent stability and has become a widely used tool, albeit with more moderate reactivity. Other notable designs, such as biarylazacyclooctynone (BARAC),^8^ have also been introduced to attain a favorable combination of these properties.^9^ Therefore, the rational design of novel scaffolds that possess both high stability for ease of handling and sufficient strain for rapid kinetics remains a critical challenge in the field.^10^

**Fig. 1:**
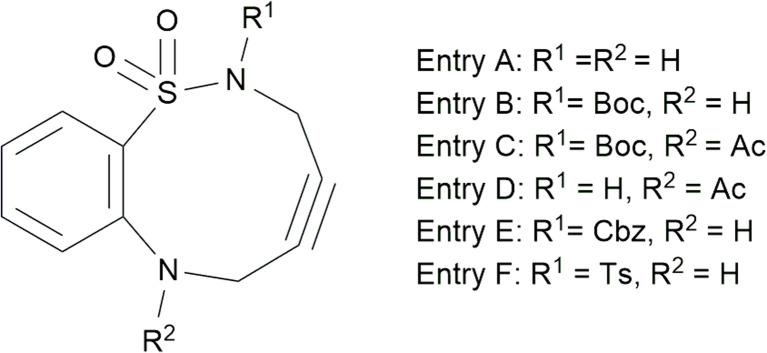
ABSACN derivatives.

In this context, this study focused on 2-aminobenzenesulfonamide-containing cyclononyne (ABSACN), a novel cyclononyne derivative with unique characteristics (Fig. 1). We hypothesized that the rigid 2-aminobenzenesulfonamide backbone and the potential for intramolecular hydrogen bonding in ABSACN would confer enhanced stability compared to highly reactive reagents, such as DIFO, while maintaining sufficient ring strain for effective SPAAC reactions. The presence of an S–N bond and intramolecular hydrogen bonding in ABSACN ensures its higher crystallinity and stability than those of conventional cycloalkynes, which are potentially advantageous for its synthesis and application in SPAAC reactions.

Previously,^11^ we reported the synthesis of ABSACN and detailed its solid-state structure using single-crystal X-ray analysis, which showed that the intramolecular hydrogen bonding of ABSACN significantly influences the SPAAC reaction. Furthermore, the regioselectivity (positional selectivity) of this reaction, specifically the ratio of major-to-minor products, was affected by the different N-substituents of the ABSACN derivatives.

## Experimental

### Computational methods

#### Reaction mechanism and frontier molecular orbital analysis of ABSACN with methyl azide using simplified models

In this study, we first attempted to analyze the reaction mechanism by performing transition-state (TS) calculations using density functional theory (DFT; B3LYP-D3(BJ)/6-31G(d))^12^ on simplified model molecules with the GAMESS program^13^ and Winmostar as the graphical user interface.^14^ We used the experimentally determined solid-state structures of ABSACN and its click reaction product (obtained *via* single-crystal X-ray analysis) as initial coordinates.

**Scheme 1:**
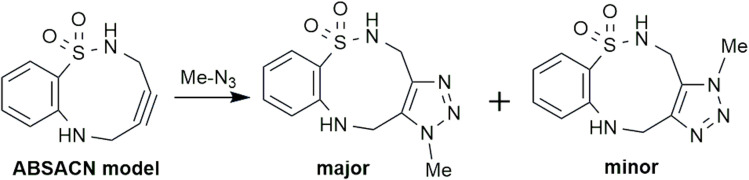
Click reaction of the model system: ABSACN and MeN_3_.

We investigated the reaction mechanism using TS structures with simplified model frameworks. Regarding TS structures, preliminary calculations were performed incrementally using the semi-empirical molecular orbital method PM7,^15^ and subsequently, calculations were conducted at the B3LYP-D3(BJ)/6-31G(d) level. For the nine-membered ring (ABSACN) model molecule, a structure containing sulfonamide, benzene, and alkyne moieties was used. For benzyl azide (BnN_3_), we used methyl azide (MeN_3_) (Scheme 1) to account for the rotational freedom of the benzene moiety and to avoid steric issues. Calculations for ground state (GS) geometry optimization and TS structures were performed at the B3LYP-D3(BJ)/6-31G(d) level of theory. Optimization calculations were performed for each model molecule, and the orbital coefficients and energies were compared.

#### Reactivity evaluation with solvent effects

Subsequently, we explored the reactivity of ABSACN derivatives in a reaction solvent, considering the solvent effect using the Polarizable Continuum Model (PCM)^16^ (B3LYP-D3(BJ)/6-31G(d)/PCM; solvent = METHYCL or solvent = ACETNTRL). This calculation specifically accounts for dichloromethane (CH_2_Cl_2_) or acetonitrile (CH_3_CN), which were used as the reaction solvent.

## Results

This section presents the computational results for SPAAC of ABSACN derivatives. We first analyze the frontier molecular orbitals (FMOs)^17^ of a model system, followed by the energetic and structural analysis of TSs for various substituted ABSACN derivatives.

### Frontier molecular orbital of the model system: reaction of ABSACN with methyl azide

To elucidate the fundamental electronic nature of the reaction, we first performed calculations on a model system: the reaction of the ABSACN (R^1^=R^2^=H) with methyl azide (MeN_3_). The calculated HOMO and LUMO energies of ABSACN were −6.1008 eV and −0.3701 eV, respectively, while those of MeN_3_ were −6.8545 eV and −0.6667 eV. The energy gap between the HOMO of ABSACN and the LUMO of MeN_3_ (Δ*E*_1_ = 5.4341 eV) was found to be smaller than that between the LUMO of ABSACN and the HOMO of MeN_3_ (Δ*E*_2_ = 6.4844 eV). This result indicates that the reaction proceeds *via* an inverse electron-demand cycloaddition (Table 1), where ABSACN acts as the HOMO donor and MeN_3_ as the LUMO acceptor.^18^

**Table 1:** HOMO and LUMO energies (table) and orbital diagrams (figure) of ABSACN and methyl azide

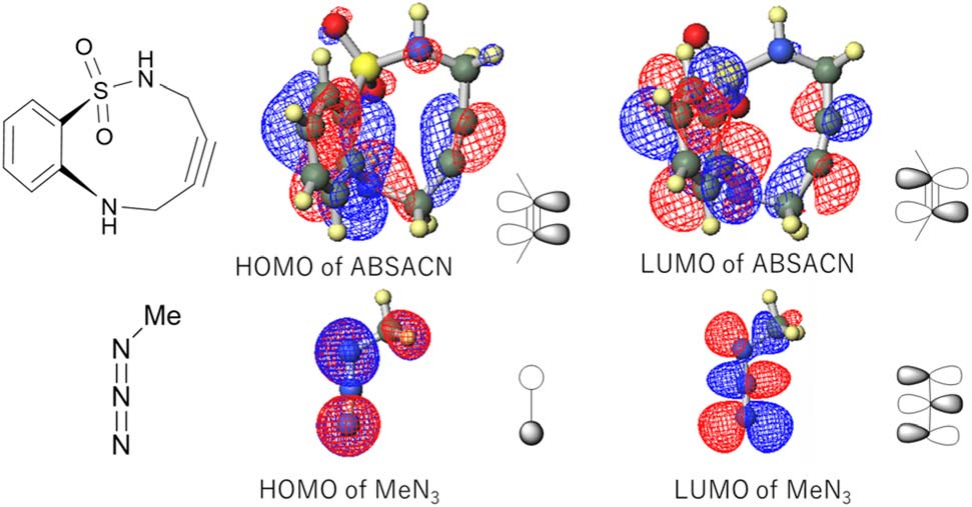		
	*E*_HOMO_ (eV)	*E*_LUMO_ (eV)
ABSACN (R^1^=R^2^=H)	−6.1008	−0.3701
Methyl azide (MeN_3_)	−6.8545	−0.6667

### Reactivity of N-substituted ABSACN derivatives

We then investigated the effect of N-substituents (R^1^ and R^2^) on the reactivity and selectivity of ABSACN. Table 2 summarizes the key computational results, including FMO energies and alkyne bond angles, alongside previously reported experimental reactivity data.^11^

**Table 2:** Experimental reactivity and calculated properties of N-substituted ABSACN derivatives

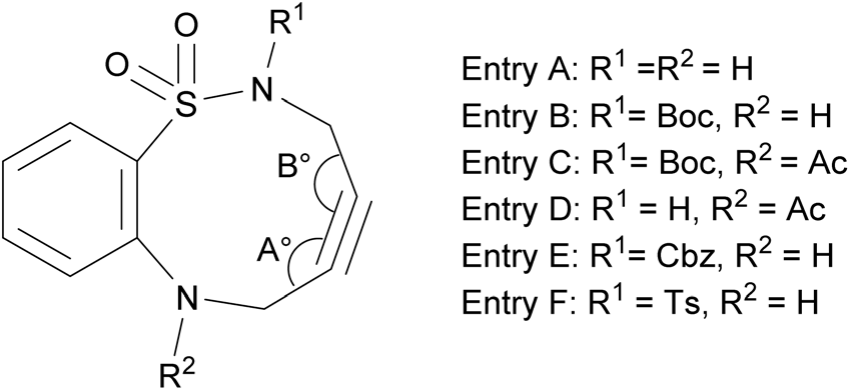				
Entry (reactivity)	HOMO (eV)	HOMO–LUMO gap (eV)	Angle (A°/B°)	Alkyne bending
A (0.43)	−6.193	5.371	158.1/166.0	18.0
B (1.50)	−6.123	5.301	155.1/162.0	21.5
C (0.74)	−6.719	5.897	160.2/162.0	18.9
D (0.27)	−6.797	5.972 (6.770)	162.9/164.4	16.4
E (2.10)	−6.142	5.320	155.4/164.0	20.3
F (1.30)	−6.234	5.412 (5.633)	157.4/167.0	17.8

### Transition state analysis for the model system

**Fig. 2:**
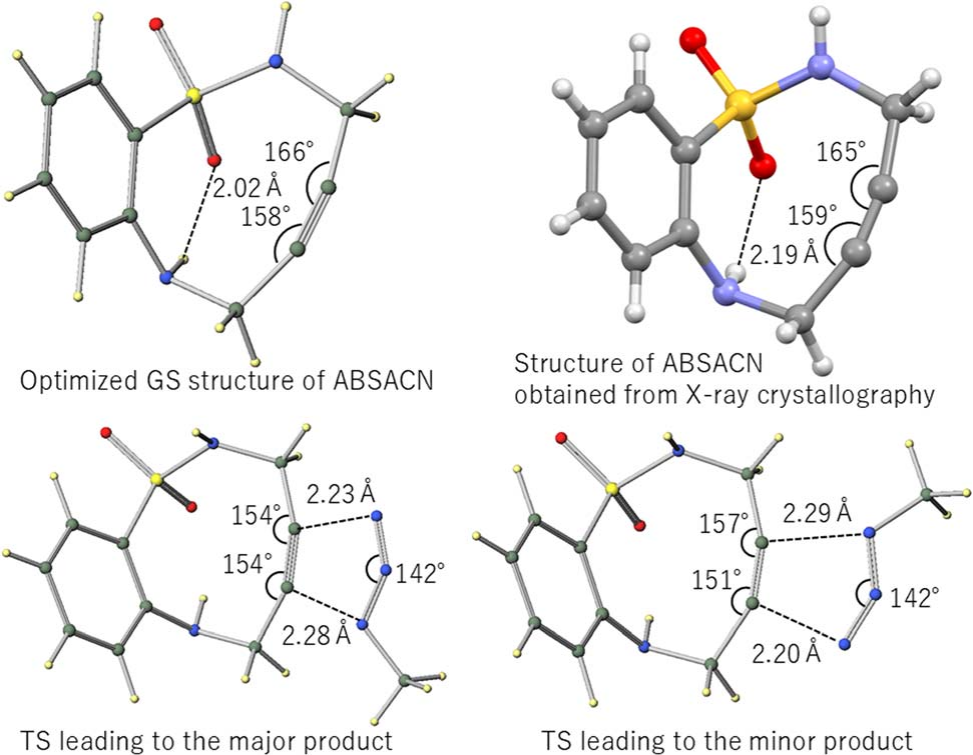
Optimized GS structure of ABSACN and structure of ABSACN obtained from X-ray crystallography^19^ and TS structures for the model reaction between ABSACN and MeN_3_: TS leading to the (left) major and (right) minor products.

We next located the TS structures for the reaction between ABSACN and MeN_3_, leading to both major and minor regioisomeric products (Fig. 2, bottom).

A comparison of the optimized GS structure of ABSACN with its TS structures reveals significant changes in the alkyne geometry. For the major product pathway, the alkyne angle on the sulfonamide side deforms from 166° to 154°, whereas the corresponding N⋯C distance is 2.23 Å. For the minor product, this angle is 157° at a distance of 2.29 Å. Conversely, the alkyne angle on the amine side changes from 158° to 154° (major) and 151° (minor).

The calculated activation energies for the major and minor pathways were 5.98 kcal mol^−1^ and 6.30 kcal mol^−1^, respectively (Table 3). The slight energy difference of 0.32 kcal mol^−1^ suggests that both products can be formed, with a slight preference for the major regioisomer.

**Table 3:** Calculated potential energies of reactants, products, and TS structures using the B3LYP-D3(BJ)/6-31G(d) level of theory^a^

Model (ABSACN + MeN_3_)		
State	Major	Minor
GS1	−1248.68113 au (0.0 kcal mol^−1^)	−1248.68113 au (0.0 kcal mol^−1^)
TS	−1248.67161 au (5.98 kcal mol^−1^)	−1248.67109 au (6.30 kcal mol^−1^)
GS2	−1248.81829 au (−86.07 kcal mol^−1^)	−1248.81597 au (−84.62 kcal mol^−1^)

a GS1 serves as the common ground state for both pathways.

### Reaction of unsubstituted ABSACN with benzyl azide

**Fig. 3:**
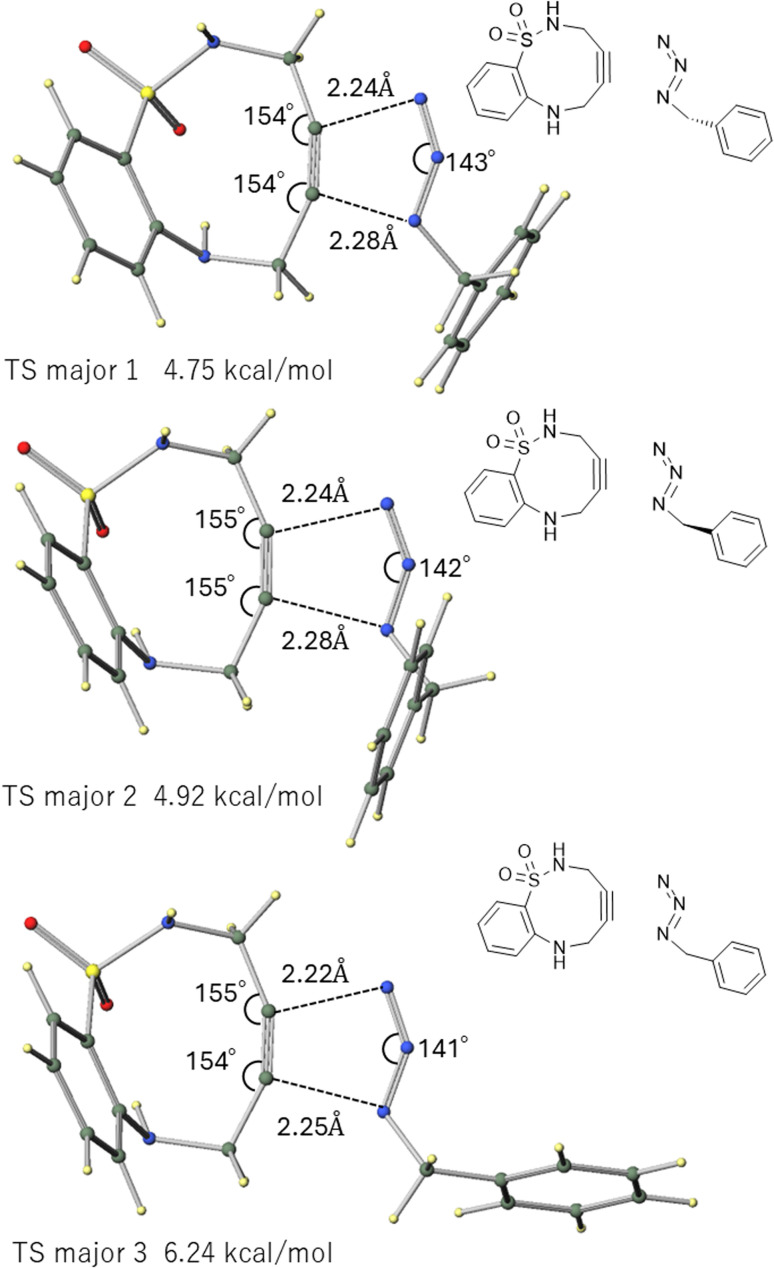
TS structures leading to the major products in the reaction between ABSACN and BnN_3_.

**Fig. 4:**
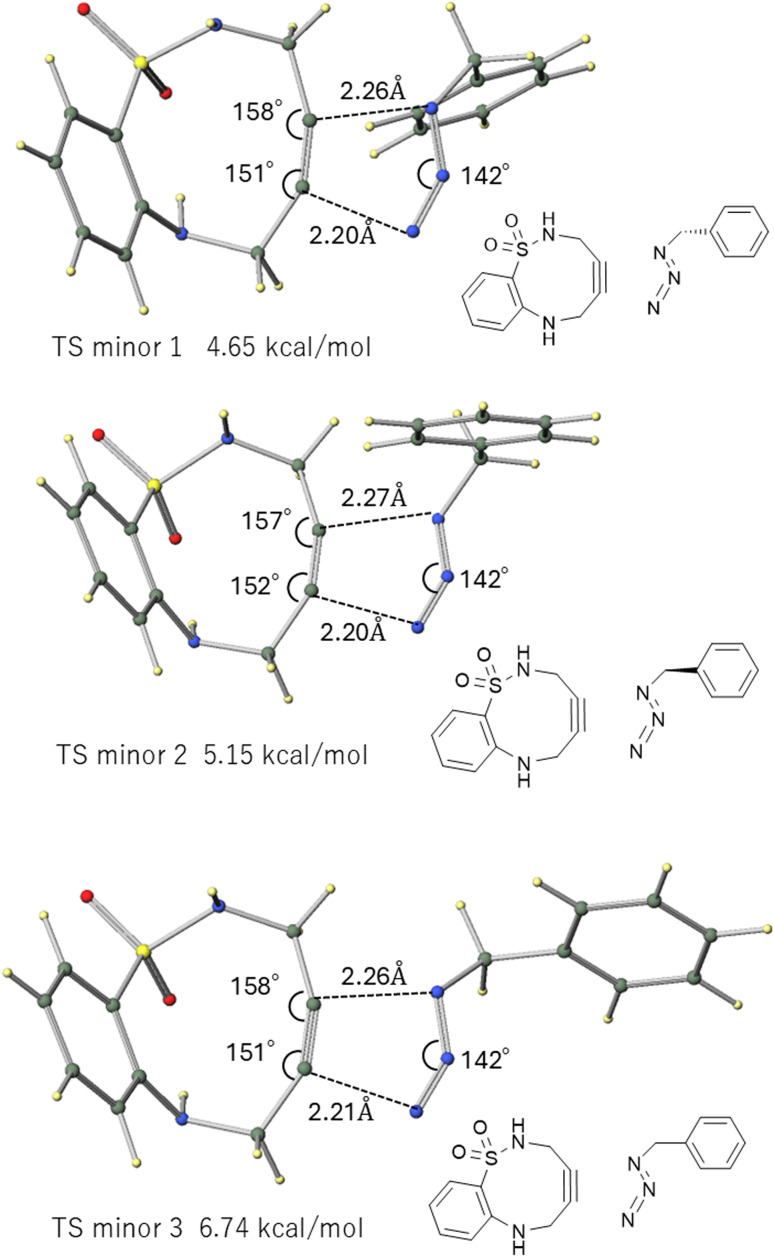
TS structures leading to the minor products in the reaction between ABSACN and BnN_3_.

To better model the experimental system, we calculated the TS structures for the reaction between ABSACN (R^1^=R^2^=H) and benzyl azide (BnN_3_). Considering the rotational freedom of the benzyl group, we identified three primary TS structures, corresponding to different orientations of the benzene rings for each regioisomeric pathway (Fig. 3 and 4).

For the major product, comparing the three TS structures—two conformers where the benzene rings of ABSACN and BnN_3_ are in relatively greater proximity (major 1 [Fig. 3, top] and major2 [Fig. 3, middle]), and one conformer where the benzene rings of ABSACN and BnN_3_ are oriented away from each other (major3 [Fig. 3, bottom])—the conformer with the benzene rings oriented away from each other (major 3) was found to be the most unstable. The respective TS energies are 4.75 (major 1), 4.92 (major 2), and 6.24 kcal mol^−1^ (major 3). Similarly, for the minor product, the TS in which the benzene rings are in relatively greater proximity (minor 1 [Fig. 4, top] and minor 2 [middle]) were more stable by 4.65 kcal mol^−1^ and 5.15 kcal mol^−1^, respectively. The conformer with the benzene rings oriented away from each other (minor 3) was the most unstable, with an energy of 6.24 kcal mol^−1^. The lowest-energy TS for the major pathway (major 1: 4.75 kcal mol^−1^) was 0.1 kcal mol^−1^ less stable than that for the minor pathway (minor 1: 4.65 kcal mol^−1^). The product structures were also calculated, revealing that the major product was 2.0 kcal mol^−1^ more stable than the minor product.

### Transition state energetics for substituted ABSACN derivatives

The calculated TS energies energies, including intermolecular interactions and solvent effects, for various substituted systems (entries A and B) are presented in Table 4. For the unsubstituted entry A, the lowest TS energies leading to the major and minor products were 5.62 kcal mol^−1^ and 6.14 kcal mol^−1^, respectively. For entry B (R^1^=Boc, R^2^=H), the corresponding values were 4.13 and 4.54 kcal mol^−1^, respectively. Calculated reaction energies indicated that all reactions were strongly exergonic.

**Table 4:** Calculated potential energies of TS structures relative to the reactants (set to 0.0 kcal mol^−1^)

Entry	Pathway		(kcal mol^−1^)
A	Major	TS1	5.62
		TS2	7.00
		TS3	7.11
	Minor	TS1	6.14
		TS2	6.57
		TS3	8.63
B	Major	TS1	4.13
		TS2	5.30
		TS3	6.05
	Minor	TS1	4.54
		TS2	5.67
		TS3	4.83
D (CH_2_Cl_2_)	Major	TS1	6.84
	Minor	TS1	7.80
D (CH_3_CN)	Major	TS1	6.19
	Minor	TS1	7.80

**Fig. 5:**
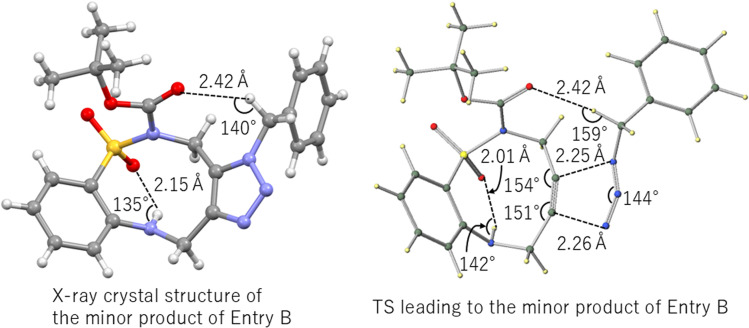
Key TS structures for the reaction of entry B (R^1^=Boc, R^2^=H) with BnN_3_.

Finally, we analyzed the minor product of entry B (R^1^=Boc, R^2^=H), which was confirmed by X-ray crystallography^20^ and the TS structure leading to it (Fig. 5). The TS calculations confirmed the presence of a C=O⋯H–C intermolecular hydrogen bond (O⋯H: 2.42 Å, ∠C–H⋯O = 159°)^21^ and a S=O⋯H–N intramolecular hydrogen bond (O⋯H: 2.01 Å, ∠N–H⋯O = 142°).

The deformation analysis of the TS for entry B shows that the strain is primarily released through the flexible deformation of the alkyne angle on the sulfonamide side (angle B), which changes by 8° (from 162° to 154°) for the minor product (Table 2 entry B; angle (A°/B°) and Fig. 5). This highlights its importance in lowering the activation barrier.

## Discussion

These results provide significant insights into the factors governing the reactivity and selectivity of ABSACN derivatives in SPAAC reactions. Here, we interpret these findings in the context of steric effects, electronic properties, intramolecular interactions, and alkyne strain, comparing them with established principles and literature precedents.

### Influence of electronic effects and alkyne strain

The reactivity trends can be rationalized by considering both FMO theory^17^ and ring strain. As shown in Table 2, electron-donating groups (Boc, Cbz) raise the HOMO energy of ABSACN, narrowing the HOMO–LUMO gap for an inverse electron-demand reaction and thus enhancing reactivity. Conversely, electron-withdrawing groups (Ac) lower the HOMO energy and decrease reactivity.

An interesting case is entry F, where a strong electron-withdrawing tosyl group lowers both the HOMO and LUMO levels significantly. Here, the energy gap for a normal electron-demand pathway (5.412 eV) becomes remarkably close to that of an inverse electron-demand pathway (5.633 eV).^18^ This suggests a potential mechanistic crossover, where both pathways could be operative, a phenomenon not observed in the other derivatives.

Ring strain also plays a crucial role. Bulky substituents such as Boc and Cbz (entries B and E), increase the alkyne bending angle (21.5 and 20.3), indicating greater strain. This predistortion of the alkyne toward the TS geometry is associated with higher reactivity.

### Intermolecular interaction governs regioselectivity, overriding steric repulsion

In the model calculation using MeN_3_, the pathway leading to the major product was 0.32 kcal mol^−1^ more stable than the pathway leading to the minor product, resulting in a slight preference for the major product (Table 3). However, this trend changes in the BnN_3_ system, which more faithfully reflects the experimental conditions. In the BnN_3_ system, conformers where the benzyl group is in great proximity to the ABSACN (major 1, major 2, minor 1, minor 2) are significantly stabilized by over 1.3 kcal mol^−1^ compared to the conformers where they are oriented away from each other (major 3, minor 3). Our calculations indicate that the regioselectivity of the reaction involving this ABSACN derivative is determined by a combination of multiple factors in the TS, going beyond simple steric hindrance. These factors include intermolecular interactions and the strain energy of the ABSACN backbone. In particular, for the reaction between unsubstituted ABSACN and BnN_3_, the reaction pathway that minimizes the influence of steric hindrance, intermolecular interactions, and the strain energy of ABSACN is the most energetically favorable (Fig. 3 and 4). This finding is consistent with recent research trends exploring a crucial paradigm in the design of SPAAC reactions: the balance between destabilization of the reagent (strain) and stabilization of the TS (electronic and non-covalent effects).^22,23^

### Crucial role of solvent effects verified by comparison with vacuum calculations

This point is critical for validating our computational model. A direct comparison of the calculations for entry A (unsubstituted ABSACN with BnN_3_) with and without solvent effects highlights this necessity.

### Comparison of entry A

The gas-phase calculation (analyzed in the “Reaction of unsubstituted ABSACN with benzyl azide” section) predicted the lowest-energy TS for the minor pathway (minor 1: 4.65 kcal mol^−1^) to be 0.1 kcal mol^−1^ more stable than the TS for the major pathway (major 1: 4.75 kcal mol^−1^). This gas-phase result incorrectly predicts the minor product as the kinetically favored product.

In stark contrast, our calculations including solvent effects (PCM, presented in Table 4) for entry A show that the major TS (TS1: 5.62 kcal mol^−1^) is 0.52 kcal mol^−1^ more stable than the minor TS (TS1: 6.14 kcal mol^−1^). This solvent-included model correctly predicts the major product as the favored regioisomer. Based on the calculated gas-phase TS energy difference of 0.1 kcal mol^−1^ for entry A, the Curtin–Hammett principle^24^ predicts a major-to-minor product ratio of approximately 54 : 46 at 298.15 K. However, calculations including solvent effects (using the 0.52 kcal mol^−1^ difference) predict a major-to-minor product ratio of approximately 71 : 29. This calculated ratio is in excellent agreement with the experimental value (5 : 2, or 71.4 : 28.6),^11^ validating our computational model. This demonstrates that even subtle conformational preferences in the TS, driven by non-bonded interactions, can dictate the final product distribution.

### Correlation between TS energies and experimental reactivity

The calculated activation energies (Table 4) show excellent agreement with the previously reported experimental relative reactivities (entry A: 0.43; entry B: 1.50; entry D: 0.27).^11^ For example, entry B, which exhibit high experimental yields (99%), have the lowest calculated activation energies (4.13–6.05 kcal mol^−1^). Conversely, the unsubstituted entry A and the less reactive entry D have higher activation barriers (5.62–8.63 kcal mol^−1^, 6.19–7.80). This strong correlation confirms that the reaction rate is primarily governed by the height of the TS energy barrier.

The case of entry D further highlights the importance of reaction conditions. While the calculated activation energy is high, the experimental yield improves dramatically from near zero in CH_2_Cl_2_ to 88% in CH_3_CN. Our calculations show similar TS energies in both solvents, indicating that the experimental outcome is dominated by the improved solubility of the starting material in acetonitrile, thereby resolving a kinetic bottleneck. Nevertheless, the yield remains lower than for entries A and B, consistent with its higher intrinsic activation barrier.

### Role of intramolecular hydrogen bonding

A key structural feature of the ABSACN scaffold is the presence of an N–H⋯O=S intramolecular hydrogen bond, observed in the crystal structure (Fig. 2) and maintained in the calculated TS structures (Fig. 5). This interaction likely contributes to the notable stability and crystallinity of ABSACN derivatives compared to other cycloalkynes. The retention of this hydrogen bond even in the TS suggests that it acts as a conformational lock, pre-organizing the molecule for the reaction and potentially stabilizing the TS structure, thereby contributing to its overall reactivity profile.

## Conclusions

DFT calculations elucidated the SPAAC reaction mechanism and selectivity of the ABSACN derivatives. A model system revealed that ABSACN acts as a HOMO donor, with minimal energy difference between the TSs of the major and minor products. For the reaction of ABSACN (R^1^=R^2^=H) with BnN_3_, it was revealed that the selectivity cannot be explained solely by simple steric repulsion in the TS. Instead, it is shown to be governed by a delicate balance between the structural distortion energy (Δ*E*_dist_) of the entire host molecule—required to achieve the optimal geometric arrangement (*ca.* 2.2 Å internuclear distance) at the reaction centers—and the intermolecular interaction energy (Δ*E*_int_). The most energetically favorable pathway (leading to the major product) corresponds to the optimal route that minimizes this overall distortion cost of the host molecule while maximizing the orbital interactions with the azide. Conversely, other approach trajectories that appear sterically accessible are suggested to be disfavored, as they impose excessive structural strain (*e.g.*, ring torsion) on the host molecule to reach the TS, resulting in a higher overall activation energy. In N-substituted ABSACN (R^1^=Boc), intramolecular hydrogen bonding^21^ was crucial for TS formation, and the calculated product ratios agree well with the experimental data. These results strongly suggest that the reactivity and selectivity of ABSACN in SPAAC reactions are precisely controlled by a combination of the alkyne strain, structural distortion in the TS, electronic substituent effects, intramolecular hydrogen bonding, and steric interactions. To validate the computational method used in this study, we also attempted TSs calculations for entry A in a vacuum, without considering solvent effects. The results showed that the TS leading to the minor product was more stable than that leading to the major product, contradicting the experimentally observed selectivity. This strongly supports the idea that including solvent effects *via* PCM is crucial for accurately reproducing the experimental results in this reaction system. Concurrently, it also suggests that a computational level such as B3LYP-D3(BJ)/6-31G(d) alone may have limitations in accurately evaluating intermolecular interaction energies. Therefore, higher-precision calculations (*e.g.*, using other density functionals with dispersion corrections (M06-2X, ω97X-D, *etc.*) or larger basis sets) remain a subject for future work. Notably, methyl azide was used as a simplified model for the initial mechanistic analysis. While this provided insight into the fundamental reactivity, it may not fully capture all steric and electronic effects imparted by the benzyl group in the actual TSs.

## Author contributions

The manuscript was written through the contributions of all authors.

## Conflicts of interest

There are no conflicts to declare.

## Supplementary Material

RA-016-D5RA06229B-s001

## Data Availability

All data are available in the main text or the supporting information (SI). Supplementary information is available. See DOI: https://doi.org/10.1039/d5ra06229b.
