# Supplementary material for: Influence of intramolecular interactions and transition state energetics on the strain-promoted azide–alkyne cycloaddition of 2-aminobenzenesulfonamide-containing cyclononynes
Source: RSC Adv. 2026 Jan 5;16(2):1030–7. doi: 10.1039/d5ra06229b (PMC12766270; doi:10.1039/d5ra06229b)
Supplement: RA-016-D5RA06229B-s001 [file RA-016-D5RA06229B-s001.pdf]

Supplementary Information (SI) for RSC Advances.
This journal is © The Royal Society of Chemistry 2025

Supporting Information

Influence of Intramolecular Interactions and Transition State Energetics on the Strain-Promoted Azide-Alkyne Cycloaddition of 2-Aminobenzenesulfonamide-Containing Cyclononynes

Kazuhide Nakahara* and Kyosuke Kaneda

Hokkaido Pharmaceutical University School of Pharmacy, 7-15-4-1 Maeda Teine Sapporo, Hokkaido 006-8590, Japan

*E-mail: nakahara-k@hus.ac.jp

Table of Contents

S1. Computational Methods · 3

S2. Tables of Calculated Energies · 4

S3. Cartesian Coordinates of Optimized Structures · 45

S1. Computational Methods

Computational Details

All structural optimizations and frequency analyses in this study were performed based on density functional theory (DFT). The calculations were carried out using the GAMESS (version June 30, 2023 R1) software package.

Theoretical Level
The level of theory was a combination of the B3LYP functional and the 6-31G(d) basis set, with the DFT-D3(BJ) method used to incorporate dispersion corrections. A high-precision TIGHT GRID was used for the integration grid.

Adoption of the DFT-D3(BJ) method: This method efficiently adds a D3 correction to account for long-range van der Waals forces (dispersion forces) that are not fully captured by the standard B3LYP functional. The (BJ) stands for the Becke-Johnson damping function, which improves the accuracy of the calculation by properly handling short-range repulsions. This allows for a more accurate description of both intermolecular and intramolecular non-covalent interactions.

Convergence Criteria: The convergence criterion for geometry optimization was set to OPTTOL = 0.00045 (Hartree/Bohr). The SCF convergence criterion was set to CONV = 1.0×10^{-5}.

Initial Structure Preparation
Initial structures for the DFT calculations were prepared using different methods depending on the target:
For stable structures, experimental geometries determined by single-crystal X-ray analysis were used as the initial coordinates.
For the exploration of transition state (TS) structures, a preliminary search was first conducted using the semi-empirical PM7 method with MOPAC2016 via the Winmostar (V11.8.6) interface.

Final Optimization and Solvent Effects
For this optimization, calculations were first performed under solvent-free conditions using the B3LYP-6-31G(d) method. Furthermore, optimization calculations considering the solvent effect were carried out using the structure obtained from the solvent-free conditions as the initial coordinates. The solvent effects of either dichloromethane (CH_2Cl_2) or acetonitrile (CH_3CN) were incorporated using the polarizable continuum model (PCM).

Characterization of Stationary Points
The nature of all stationary points was determined by frequency analysis:
Reactants and products were confirmed as local minima with no imaginary frequencies.
Transition states were confirmed as first-order saddle points with exactly one imaginary frequency. For instance, the imaginary frequencies for key transition states were:
Model, Major TS: -327 cm^{-1}
Model, Minor TS: -335 cm^{-1}

Unless otherwise noted, the energy values discussed in this paper include zero-point vibrational energy (ZPVE) corrections calculated at 298.15 K. No scaling factor was applied to the vibrational frequencies.

S2. Tables of Calculated Energies

Computational Results
The absolute energies for each computed species and the imaginary frequencies for the transition states are provided below. The atomic coordinates (in XYZ format) provided at the end of this Supporting Information.

Model Reactants (Table 1 & Figure 2 & Table 3)

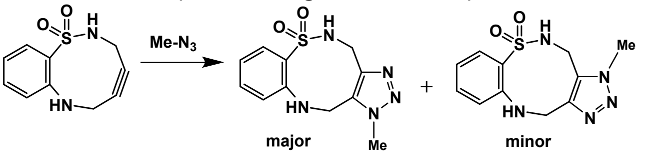

Figure S1. Formation of regioisomers in the strain-promoted cycloaddition of model molecule ABSACN with methyl azide.

Table S1. Total energies and frequency analysis results at the B3LYP-D3(BJ)/6-31G(d) level for model reactants (ABSACN and methyl azide), key transition states, and products.

Molecule/State	Total Energy (E)	No. of Imaginary Frequencies	Imaginary Frequency (cm^{-1})
ABSACN model	-1044.69247	0	-
Methyl azide	-203.98866	0	-
TS major	-1248.67161	1	-327
TS minor	-1248.67109	1	-335
GS2 Major product	-1248.81829	0	-
GS2 Minor product	-1248.81597	0	-

Model Reactants and Products
Total Reactant (E): E (ABSACN model) + E (Methyl azide) = -1044.69247 a.u.+(-203.98866 a.u.) =-1248.68113 a.u.= -1248.68113 a.u. × 627.51 kcal/mol/a.u.= -783637.38 kcal/mol

Activation Barrier for TS major: (-1248.67161 a.u.) − (-1248.68113 a.u.) =0.00952 a.u. =0.00952 a.u.×627.51 kcal/mol/a.u.= 5.976 kcal/mol
Activation Barrier for TS minor: (-1248.67109 a.u.) − (-1248.68113 a.u.) = 0.01004 a.u. =0.01004 a.u.×627.51 kcal/mol/a.u.= 6.300 kcal/mol

Reaction Energy for GS2 Major product: (-1248.81829 a.u.) − (-1248.68113 a.u.) =−0.13716 a.u. =−0.13716 a.u.×627.51 kcal/mol/a.u.= -86.06882 kcal/mol
Reaction Energy for GS2 Minor product: (-1248.81597 a.u.) − (-1248.68113 a.u.) = −0.13484 a.u. =−0.13484 a.u.×627.51 kcal/mol/a.u.= -84.61510 kcal/mol

ABSACN model: The optimized ground state structure (GS1) representing the total energy of the reactants (model molecules: optimized ABSACN Entry A and optimized MeN_3).

Table S2. Calculated thermodynamic quantities and frontier orbital energies for ABSACN model (GS1).

	E (kcal/mol)	H (kcal/mol)	G (kcal/mol)	CV (cal/(mol·K))	CP (cal/(mol·K))	S (cal/(mol·K))
ELEC.	0	0	0	0	0	0
TRANS.	0.889	1.481	-11.07	2.981	4.968	42.096
ROT.	0.889	0.889	-8.576	2.981	2.981	31.745
VIB.	127.014	127.014	116.772	43.919	43.919	34.35
TOTAL	128.791	129.384	97.127	49.881	51.868	108.191
VIB. THERMAL CORRECTION E(T)-E(0) = H(T)-H(0) = 6032.873 cal/mol						
Orbital Energy: HOMO: -6.1008 eV, LUMO: -0.3701 eV						

Note: All values are calculated at the B3LYP-D3(BJ)/6-31G(d) level. ELEC., TRANS., ROT., and VIB. denote electronic, translational, rotational, and vibrational contributions, respectively.

Methyl azide Model: The optimized ground state structure (GS1) representing the total energy of the reactants (model molecules: optimized ABSACN Entry A and optimized MeN_3).

Table S3. Calculated thermodynamic quantities and frontier orbital energies for Methyl azide Model (GS1).

	E (kcal/mol)	H (kcal/mol)	G (kcal/mol)	CV (cal/(mol·K))	CP (cal/(mol·K))	S (cal/(mol·K))
ELEC.	0	0	0	0	0	0
TRANS.	0.889	1.481	-9.862	2.981	4.968	38.044
ROT.	0.889	0.889	-6.007	2.981	2.981	23.129
VIB.	32.807	32.807	31.022	7	7	5.986
TOTAL	34.584	35.177	15.153	12.962	14.949	67.159
VIB. THERMAL CORRECTION E(T)-E(0) = H(T)-H(0) = 1007.794 cal/mol						
Orbital Energy: HOMO: -6.8545 eV, LUMO: -0.6667 eV						

TS major Model: The transition state structure leading to the major product in the click reaction between the model molecules, ABSACN Entry A and MeN_3.

Table S4. Calculated thermodynamic quantities and energy components for the transition state leading to the major product (TS major).

	E (kcal/mol)	H (kcal/mol)	G (kcal/mol)	CV (cal/(mol·K))	CP (cal/(mol·K))	S (cal/(mol·K))
ELEC.	0.000	0.000	0.000	0.000	0.000	0.000
TRANS.	0.889	1.481	-11.273	2.981	4.968	42.777
ROT.	0.889	0.889	-9.143	2.981	2.981	33.645
VIB.	162.681	162.681	145.073	60.212	60.212	59.056
TOTAL	164.458	165.051	124.658	66.174	68.161	135.478
VIB. THERMAL CORRECTION E(T)-E(0) = H(T)-H(0) = 204.008 cal/mol						
Imaginary Frequency: -327 cm^{-1}						

Note: All values are calculated at the B3LYP-D3(BJ)/6-31G(d) level. The presence of a single imaginary frequency confirms the nature of the transition state. ELEC., TRANS., ROT., and VIB. denote electronic, translational, rotational, and vibrational contributions, respectively.

ENERGY COMPONENTS□□Value (Hartree)
WAVEFUNCTION NORMALIZATION = .0000000000
ONE ELECTRON ENERGY = -4933.3157145563
TWO ELECTRON ENERGY = 2060.7223967211
NUCLEAR REPULSION ENERGY = 1623.9217119680
TOTAL ENERGY = -1248.6716058672
ELECTRON-ELECTRON POTENTIAL ENERGY = 2060.7223967211
NUCLEUS-ELECTRON POTENTIAL ENERGY = -6172.9578084715
NUCLEUS-NUCLEUS POTENTIAL ENERGY = 1623.9217119680
TOTAL POTENTIAL ENERGY = -2488.3136997824
TOTAL KINETIC ENERGY = 1239.6420939152
VIRIAL RATIO (V/T) = 2.0072839669

TS minor Model: The transition state structure leading to the minor product in the click reaction between the model molecules, ABSACN Entry A and MeN_3.

Table S5. Calculated thermodynamic quantities and energy components for the transition state leading to the minor product (TS minor).

	E (kcal/mol)	H (kcal/mol)	G (kcal/mol)	CV (cal/(mol·K))	CP (cal/(mol·K))	S (cal/(mol·K))
ELEC.	0.000	0.000	0.000	0.000	0.000	0.000

TRANS.	0.889	1.481	-11.273	2.981	4.968	42.777
ROT.	0.889	0.889	-9.145	2.981	2.981	33.653
VIB.	162.486	162.486	144.419	60.447	60.447	60.598
TOTAL	164.263	164.856	124.001	66.408	68.396	137.027
VIB. THERMAL CORRECTION E(T)-E(0) = H(T)-H(0) = 9322.980 cal/mol						
Imaginary Frequency: -335 cm^{-1}						

Note: All values are calculated at the B3LYP-D3(BJ)/6-31G(d) level. The presence of a single imaginary frequency confirms the nature of the transition state. ELEC., TRANS., ROT., and VIB. denote electronic, translational, rotational, and vibrational contributions, respectively.

ENERGY COMPONENTS Value (Hartree)
WAVEFUNCTION NORMALIZATION = 1.0000000000
ONE ELECTRON ENERGY = -4931.3068663597
TWO ELECTRON ENERGY = 2059.6945588948
NUCLEAR REPULSION ENERGY = 1622.9412199214
TOTAL ENERGY = -1248.6710875434
ELECTRON-ELECTRON POTENTIAL ENERGY = 2059.6945588948
NUCLEUS-ELECTRON POTENTIAL ENERGY = -6170.9519777009
NUCLEUS-NUCLEUS POTENTIAL ENERGY = 1622.9412199214
TOTAL POTENTIAL ENERGY = -2488.3161988846
TOTAL KINETIC ENERGY = 1239.6451113412
VIRIAL RATIO (V/T) = 2.0072810969

GS2 Major product Model: The ground state structure (GS2) of the major product obtained from the click reaction between the model molecules, ABSACN Entry A and MeN_3.

Table S6. Calculated thermodynamic quantities and energy components for the major product (GS2 major).

	E (kcal/mol)	H (kcal/mol)	G (kcal/mol)	CV (cal/(mol·K))	CP (cal/(mol·K))	S (cal/(mol·K))
ELEC.	0	0	0	0	0	0
TRANS.	0.889	1.481	-11.273	2.981	4.968	42.777
ROT.	0.889	0.889	-8.986	2.981	2.981	33.121
VIB.	165.602	165.602	150.9	56.5	56.5	49.313
TOTAL	167.38	167.972	130.641	62.461	64.448	125.211
VIB. THERMAL CORRECTION E(T)-E(0) = H(T)-H(0) = 8097.410 cal/mol						

Note: All values are calculated at the B3LYP-D3(BJ)/6-31G(d) level. ELEC., TRANS., ROT., and VIB. denote electronic, translational, rotational, and vibrational contributions, respectively.

ENERGY COMPONENTS Value (Hartree)
WAVEFUNCTION NORMALIZATION = 1.0000000000
ONE ELECTRON ENERGY = -5099.0309852413
TWO ELECTRON ENERGY = 2143.0121817999
NUCLEAR REPULSION ENERGY = 1707.2005164971
TOTAL ENERGY = -1248.8182869443
ELECTRON-ELECTRON POTENTIAL ENERGY = 2143.0121817999
NUCLEUS-ELECTRON POTENTIAL ENERGY = -6338.9295833313
NUCLEUS-NUCLEUS POTENTIAL ENERGY = 1707.2005164971
TOTAL POTENTIAL ENERGY = -2488.7168850343
TOTAL KINETIC ENERGY = 1239.8985980899
VIRIAL RATIO (V/T) = 2.0071938857

GS2 Minor product Model: The ground state structure (GS2) of the minor product obtained from the click reaction between the model molecules, ABSACN Entry A and MeN_3.

Table S7. Calculated thermodynamic quantities and energy components for the minor product (GS2 minor).

	E (kcal/mol)	H (kcal/mol)	G (kcal/mol)	CV (cal/(mol·K))	CP (cal/(mol·K))	S (cal/(mol·K))

ELEC.	0	0	0	0	0	0
TRANS.	0.889	1.481	-11.273	2.981	4.968	42.777
ROT.	0.889	0.889	-9.007	2.981	2.981	33.191
VIB.	165.618	165.618	151.263	56.544	56.544	48.146
TOTAL	167.395	167.988	130.983	62.506	64.493	124.113
VIB. THERMAL CORRECTION E(T)-E(0) = H(T)-H(0) = 8011.266 cal/mol						

Note: All values are calculated at the B3LYP-D3(BJ)/6-31G(d) level. ELEC., TRANS., ROT., and VIB. denote electronic, translational, rotational, and vibrational contributions, respectively.

ENERGY COMPONENTS□□Value (Hartree)
WAVEFUNCTION NORMALIZATION = 1.0000000000
ONE ELECTRON ENERGY = -5084.1401563981
TWO ELECTRON ENERGY = 2135.5547013347
NUCLEAR REPULSION ENERGY = 1699.7694847638
TOTAL ENERGY = -1248.8159702997
ELECTRON-ELECTRON POTENTIAL ENERGY = 2135.5547013347
NUCLEUS-ELECTRON POTENTIAL ENERGY = -6324.0292546924
NUCLEUS-NUCLEUS POTENTIAL ENERGY = 1699.7694847638
TOTAL POTENTIAL ENERGY = -2488.7050685940
TOTAL KINETIC ENERGY = 1239.8890982943
VIRIAL RATIO (V/T) = 2.0071997342

Model IRC: The vertical axis represents Total Energy (unit: kcal/mol). Calculations were performed at the B3LYP-D3(BJ)/6-31G(d) level. The figure consists of two comparative profiles: the upper profile corresponds to the pathway leading to the major product (TS major), and the lower profile corresponds to the pathway leading to the minor product (TS minor).

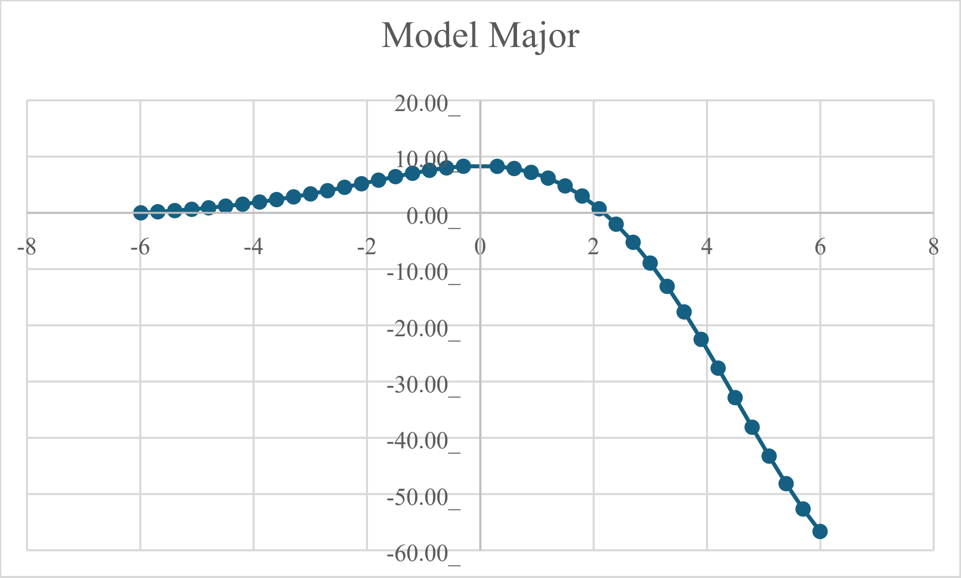

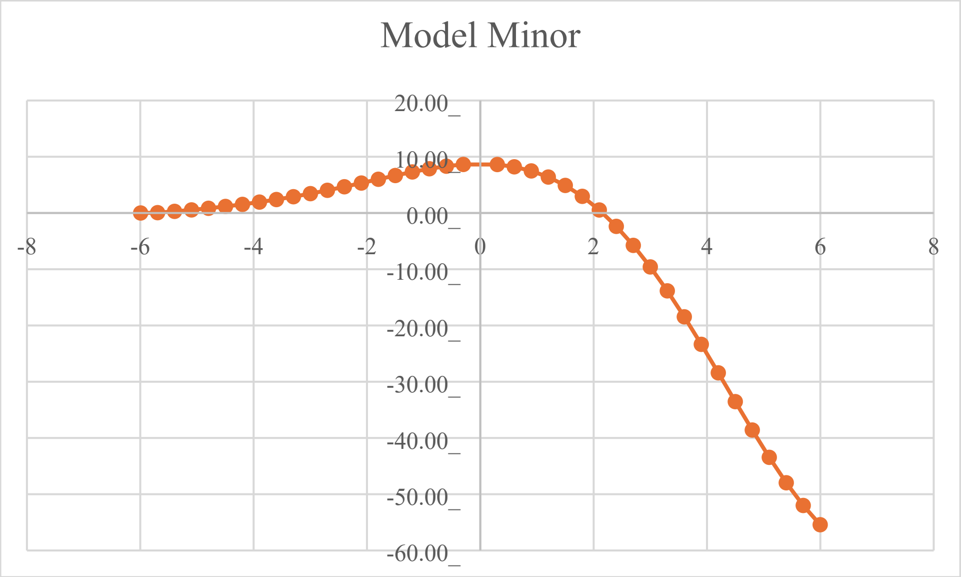

Figure S2. Comparison of potential energy profiles (IRC calculations) for the pathways leading to the major and minor products in the cycloaddition of ABSACN with methyl azide.

Model major IRC=+1

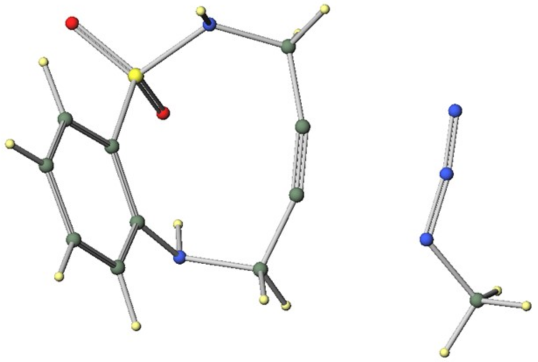

Energy -1248.68502
Distance -5.98999

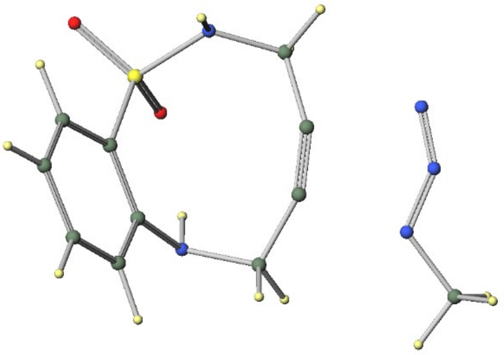

Energy -1248.67182
Distance -0.29790

Model Major IRC=-1

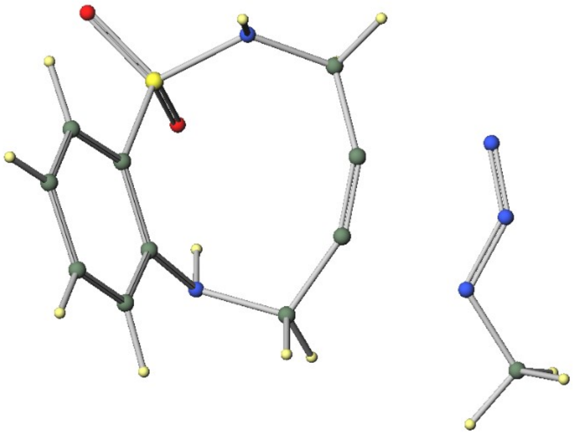

Energy -1248.67185
Distance 0.29842

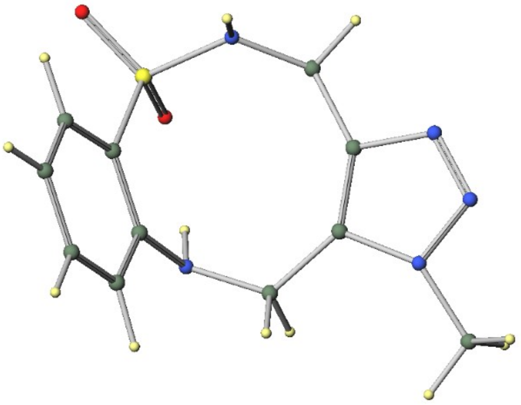

Energy -1248.77530
Distance 5.99505

Figure S3. Results of Intrinsic Reaction Coordinate (IRC) analysis corresponding to TS major
In the Model major IRC calculation, the IRC=+1 path proceeds from the transition state (Energy: -1248.67182, Distance: -0.29790) toward the reactant (Energy: -1248.68502, Distance: -5.98999). Conversely, the IRC=-1 path leads from the transition state (Energy: -1248.67185, Distance: -0.29842) toward the product (Energy -1248.77530, Distance 5.99505).

Model minor IRC=1

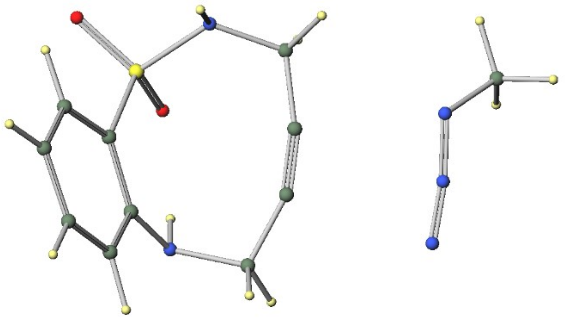

Energy -1248. 68525
Distance -5.99365

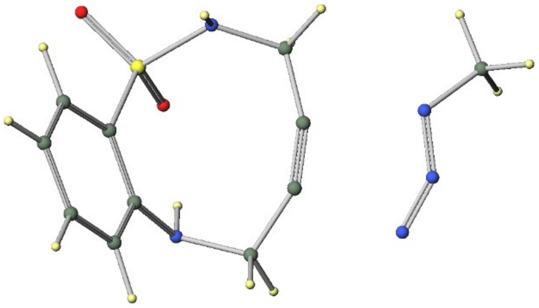

Energy -1248.67127
Distance -0.29985

Model minor IRC=-1

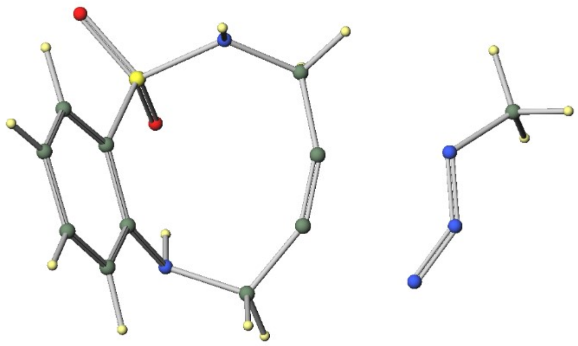

Energy -1248.67129
Distance 0.29987

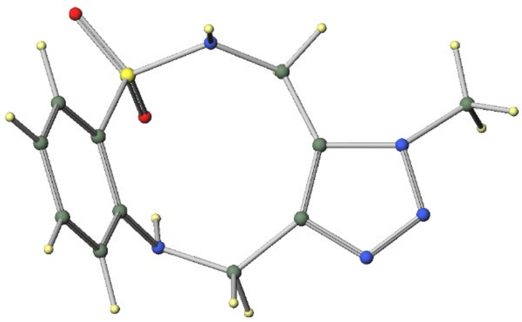

Energy -1248.77334
Distance 5.99717

Figure S4. Results of Intrinsic Reaction Coordinate (IRC) analysis corresponding to TS minor
In the Model major IRC calculation, the IRC=+1 path proceeds from the transition state (Energy: -1248.67127, Distance: -0.29985) toward the reactant (Energy: -1248.68525, Distance: -5.99365). Conversely, the IRC=-1 path leads from the transition state (Energy: -1248.67129, Distance: -0.29987) toward the product (Energy -1248.77334, Distance 5.99717).

Reaction of the ABSACN and BnN_3 (Figure 3 & 4)

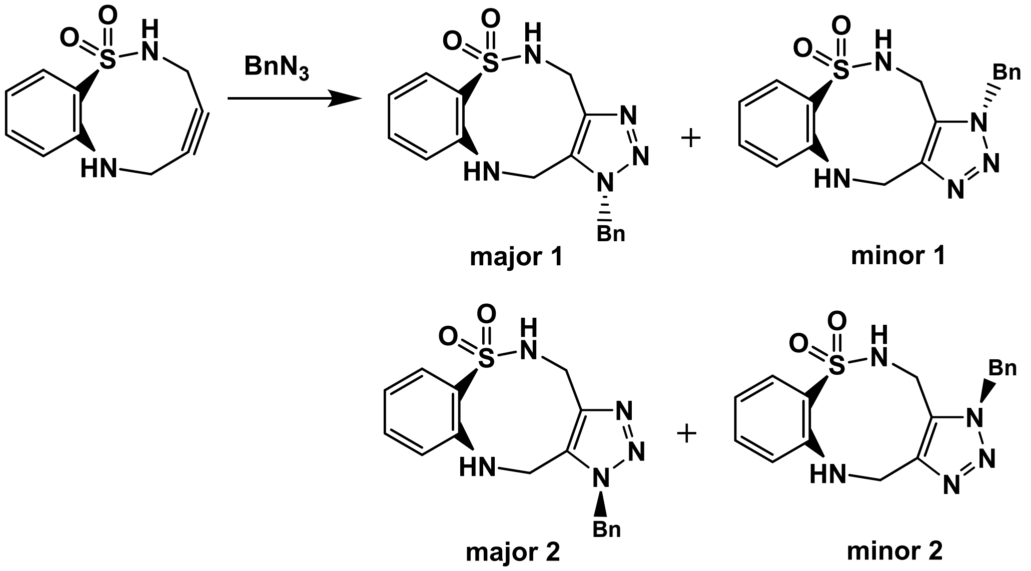

Figure S5. Formation of regioisomers in the strain-promoted cycloaddition of ABSACN (R^1=R^2=H) and Benzyl azide.

Table S8. Total energies, imaginary frequencies, activation barriers, and reaction energies calculated at the B3LYP-D3(BJ)/6-31G(d) level for the ABSACN (R^1=R^2=H) and Benzyl azide reaction.

Molecule/State	Total Energy (E)	No. of Imaginary Frequencies	Imaginary Frequency (cm^{-1})
ABSACN(R^1=R^2=H)	-1044.69247	0	-
Benzyl azide (BnN_3)	-434.91764	0	-
TS major type1	-1479.60254	1	-348
TS major type2	-1479.60226	1	-320
TS major type3	-1479.60017	1	-310
TS minor type1	-1479.60270	1	-344
TS minor type2	-1479.60190	1	-337
TS minor type3	-1479.59935	1	-329
GS2 major 1	-1479.75002	0	-
GS2 major 2	-1479.75394	0	-
GS2 minor 1	-1479.74798	0	-
GS2 minor 2	-1479.75716	0	-

Reactions with ABSACN(R^1=R^2=H) and Benzyl azide
Total Reactant (E): E(ABSACN(R^1=R^2=H)) + E(Benzyl azide (BnN_3)) = −1044.69247 a.u.+(−434.91764 a.u.)= -1479.61011 a.u.=− -1479.61011 a.u.×627.51 kcal/mol/a.u.=-928470.14 kcal/mol

Activation Barrier for TS major type1: (-1479.60254 a.u.)−(−1479.61011 a.u.)=0.00757 a.u. =0.00757 a.u.×627.51 kcal/mol/a.u.=4.750 kcal/mol
Activation Barrier for TS major type2: (-1479.60226 a.u.)−(−1479.61011 a.u.)=0.00785 a.u. =0.00785 a.u.×627.51 kcal/mol/a.u.=4.926 kcal/mol
Activation Barrier for TS major type3: (-1479.60017 a.u.)−(−1479.61011 a.u.)=0.00994 a.u. =0.00994 a.u.×627.51 kcal/mol/a.u.=6.237 kcal/mol
Activation Barrier for TS minor type1: (-1479.60270 a.u.)−(−1479.61011 a.u.)=0.00741 a.u. =0.00741 a.u.×627.51 kcal/mol/a.u.=4.649 kcal/mol
Activation Barrier for TS minor type2: (-1479.60190 a.u.)−(−1479.61011 a.u.)=0.00821 a.u. =0.00821 a.u.×627.51 kcal/mol/a.u.=5.159 kcal/mol
Activation Barrier for TS minor type3: (-1479.59935 a.u.)−(−1479.61011 a.u.)=0.01076 a.u. =0.01076 a.u.×627.51 kcal/mol/a.u.=6.752 kcal/mol

Reaction Energy for GS2 major 1: (-1479.75002 a.u.)−(−1479.61011 a.u.)=−0.13991 a.u.
=−0.13991 a.u.×627.51 kcal/mol/a.u.=-87.79 kcal/mol
Reaction Energy for GS2 major 2: (-1479.75394 a.u.)−(−1479.61011 a.u.)=−0.14383 a.u.
=−0.14383 a.u.×627.51 kcal/mol/a.u.=-90.25 kcal/mol
Reaction Energy for GS2 minor 1: (-1479.74798 a.u.)−(−1479.61011 a.u.)=−0.13787 a.u.
=−0.13787 a.u.×627.51 kcal/mol/a.u.=-86.51 kcal/mol
Reaction Energy for GS2 minor 2: (-1479.75716 a.u.)−(−1479.61011 a.u.)=−0.14705 a.u.
=−0.14705 a.u.×627.51 kcal/mol/a.u.=-92.28 kcal/mol

Entry A and Benzyl azide
GS1 Entry A ABSACN(R^1=R^2=H) **: The optimized ground state structure (GS1) representing the total energy of the reactants (optimized ABSACN Entry A and optimized BnN_3).**

Table S9. Calculated thermodynamic quantities for the ABSACN(R^1=R^2=H) reactant model (GS1).

	E (kcal/mol)	H (kcal/mol)	G (kcal/mol)	CV (cal/(mol·K))	CP (cal/(mol·K))	S (cal/(mol·K))
ELEC.	0	0	0	0	0	0
TRANS.	0.889	1.481	-11.07	2.981	4.968	42.096
ROT.	0.889	0.889	-8.576	2.981	2.981	31.745
VIB.	127.014	127.014	116.772	43.919	43.919	34.35
TOTAL	128.791	129.384	97.127	49.881	51.868	108.191
VIB. THERMAL CORRECTION E(T)-E(0) = H(T)-H(0) = 6032.873 cal/mol						

GS1 BnN_3: The optimized ground state structure (GS1) representing the total energy of the reactants (optimized ABSACN Entry A and optimized BnN_3).

Table S10. Calculated thermodynamic quantities for the Benzyl azide (BnN_3) reactant model (GS1).

	E (kcal/mol)	H (kcal/mol)	G (kcal/mol)	CV (cal/(mol·K))	CP (cal/(mol·K))	S (cal/(mol·K))
ELEC.	0	0	0	0	0	0
TRANS.	0.889	1.481	-10.615	2.981	4.968	40.569
ROT.	0.889	0.889	-7.897	2.981	2.981	29.467
VIB.	86.783	86.783	79.662	24.894	24.894	23.884
TOTAL	88.56	89.153	61.15	30.855	32.842	93.92
VIB. THERMAL CORRECTION E(T)-E(0) = H(T)-H(0) = 3515.684 cal/mol						

TS major type1: The transition state structure (Type 1) leading to the major product in the click reaction between ABSACN Entry A and BnN_3.

Table S11. Calculated thermodynamic quantities and energy components for the transition state structures leading to the major product (TS major type1) in the cycloaddition reaction of ABSACN (R^1=R^2=H) with Benzyl azide.

	E (kcal/mol)	H (kcal/mol)	G (kcal/mol)	CV (cal/(mol·K))	CP (cal/(mol·K))	S (cal/(mol·K))
ELEC.	0	0	0	0	0	0
TRANS.	0.889	1.481	-11.487	2.981	4.968	43.495
ROT.	0.889	0.889	-9.644	2.981	2.981	35.325
VIB.	216.649	216.649	192.535	78.224	78.224	80.88

TOTAL	218.426	219.019	171.404	84.185	86.173	159.701
VIB. THERMAL CORRECTION E(T)-E(0) = H(T)-H(0) = 11851.269 cal/mol						
Imaginary Frequency: -348 cm⁻¹						

Note: All values are calculated at the B3LYP-D3(BJ)/6-31G(d) level. The presence of a single imaginary frequency confirms the nature of the transition state. ELEC., TRANS., ROT., and VIB. denote electronic, translational, rotational, and vibrational contributions, respectively.

ENERGY COMPONENTS□□Value (Hartree)
WAVEFUNCTION NORMALIZATION = 1.0000000000
ONE ELECTRON ENERGY = -6680.6495265446
TWO ELECTRON ENERGY = 2856.4314520769
NUCLEAR REPULSION ENERGY = 2344.6155308196
TOTAL ENERGY = -1479.6025436481
ELECTRON-ELECTRON POTENTIAL ENERGY = 2856.4314520769
NUCLEUS-ELECTRON POTENTIAL ENERGY = -8149.1133141787
NUCLEUS-NUCLEUS POTENTIAL ENERGY = 2344.6155308196
TOTAL POTENTIAL ENERGY = -2948.0663312821
TOTAL KINETIC ENERGY = 1468.4637876341
VIRIAL RATIO (V/T) = 2.0075853120

TS major type2 : The transition state structure (Type 2) leading to the major product in the click reaction between ABSACN Entry A and BnN_3.

Table S12. Calculated thermodynamic quantities and energy components for the transition state structures leading to the major product (TS major type2) in the cycloaddition reaction of ABSACN (R^1=R^2=H) with Benzyl azide.

	E(kcal/mol)	H(kcal/mol)	G(kcal/mol)	CV(cal/(mol·K))	CP(cal/(mol·K))	S(cal/(mol·K))
ELEC.	0	0	0	0	0	0
TRANS.	0.889	1.481	-11.487	2.981	4.968	43.495
ROT.	0.889	0.889	-9.629	2.981	2.981	35.276
VIB.	216.572	216.572	192.314	78.377	78.377	81.362
TOTAL	218.350	218.942	171.199	84.339	86.326	160.133
VIB. THERMAL CORRECTION E(T)-E(0) = H(T)-H(0) = 11901.471 cal/mol						
Imaginary Frequency: -320 cm⁻¹						

Note: All values are calculated at the B3LYP-D3(BJ)/6-31G(d) level. The presence of a single imaginary frequency confirms the nature of the transition state. ELEC., TRANS., ROT., and VIB. denote electronic, translational, rotational, and vibrational contributions, respectively.

ENERGY COMPONENTS□□Value (Hartree)
WAVEFUNCTION NORMALIZATION = 1.0000000000
ONE ELECTRON ENERGY = -6697.3154208224
TWO ELECTRON ENERGY = 2864.6994067666
NUCLEAR REPULSION ENERGY = 2353.0137511901
TOTAL ENERGY = -1479.6022628657
ELECTRON-ELECTRON POTENTIAL ENERGY = 2864.6994067666
NUCLEUS-ELECTRON POTENTIAL ENERGY = -8165.7648002675
NUCLEUS-NUCLEUS POTENTIAL ENERGY = 2353.0137511901
TOTAL POTENTIAL ENERGY = -2948.0516423108
TOTAL KINETIC ENERGY = 1468.4493794451
VIRIAL RATIO (V/T) = 2.0075950071

TS major type3: The transition state structure (Type 3) leading to the major product in the click reaction between ABSACN Entry A and BnN_3. Imaginary Frequency: -310

Table S13. Calculated thermodynamic quantities and energy components for the transition state structures leading to the major product (TS major type3) in the cycloaddition reaction of ABSACN (R^1=R^2=H) with Benzyl azide.

	E (kcal/mol)	H (kcal/mol)	G (kcal/mol)	CV (cal/(mol·K))	CP (cal/(mol·K))	S (cal/(mol·K))

ELEC.	0	0	0	0	0	0
TRANS.	0.889	1.481	-11.487	2.981	4.968	43.495
ROT.	0.889	0.889	-9.765	2.981	2.981	35.733
VIB.	216.667	216.667	193.461	78.174	78.174	77.831
TOTAL	218.444	219.037	172.21	84.136	86.123	157.059
VIB. THERMAL CORRECTION E(T)-E(0) = H(T)-H(0) = 11804.217 cal/mol						
Imaginary Frequency: -310 cm^{-1}						

Note: All values are calculated at the B3LYP-D3(BJ)/6-31G(d) level. The presence of a single imaginary frequency confirms the nature of the transition state. ELEC., TRANS., ROT., and VIB. denote electronic, translational, rotational, and vibrational contributions, respectively.

ENERGY COMPONENTS Value (Hartree)
WAVEFUNCTION NORMALIZATION = 1.0000000000
ONE ELECTRON ENERGY = -6516.1220220162
TWO ELECTRON ENERGY = 2774.1009476904
NUCLEAR REPULSION ENERGY = 2262.4209085253
TOTAL ENERGY = -1479.6001658005
ELECTRON-ELECTRON POTENTIAL ENERGY = 2774.1009476904
NUCLEUS-ELECTRON POTENTIAL ENERGY = -7984.5622864717
NUCLEUS-NUCLEUS POTENTIAL ENERGY = 2262.4209085253
TOTAL POTENTIAL ENERGY = -2948.0404302560
TOTAL KINETIC ENERGY = 1468.4402644555
VIRIAL RATIO (V/T) = 2.0075998334

TS minor type1: The transition state structure (Type 1) leading to the minor product in the click reaction between ABSACN Entry A and BnN_3.

Table S14. Calculated thermodynamic quantities and energy components for the transition state structures leading to the minor product (TS minor type1) in the cycloaddition reaction of ABSACN (R^1=R^2=H) with Benzyl azide.

	E (kcal/mol)	H (kcal/mol)	G (kcal/mol)	CV (cal/(mol·K))	CP (cal/(mol·K))	S (cal/(mol·K))
ELEC.	0	0	0	0	0	0
TRANS.	0.889	1.481	-11.487	2.981	4.968	43.495
ROT.	0.889	0.889	-9.65	2.981	2.981	35.346
VIB.	216.628	216.628	193.585	78.077	78.077	77.284
TOTAL	218.405	218.998	172.449	84.038	86.025	156.126
VIB. THERMAL CORRECTION E(T)-E(0) = H(T)-H(0) = 11732.333 cal/mol						
Imaginary Frequency: -344 cm^{-1}						

Note: All values are calculated at the B3LYP-D3(BJ)/6-31G(d) level. The presence of a single imaginary frequency confirms the nature of the transition state. ELEC., TRANS., ROT., and VIB. denote electronic, translational, rotational, and vibrational contributions, respectively.

ENERGY COMPONENTS Value (Hartree)
WAVEFUNCTION NORMALIZATION = 1.0000000000
ONE ELECTRON ENERGY = -6660.9904265150
TWO ELECTRON ENERGY = 2846.4934810127
NUCLEAR REPULSION ENERGY = 2334.8942499937
TOTAL ENERGY = -1479.6026955087
ELECTRON-ELECTRON POTENTIAL ENERGY = 2846.4934810127
NUCLEUS-ELECTRON POTENTIAL ENERGY = -8129.4566522879
NUCLEUS-NUCLEUS POTENTIAL ENERGY = 2334.8942499937
TOTAL POTENTIAL ENERGY = -2948.0689212816
TOTAL KINETIC ENERGY = 1468.4662257729

VIRIAL RATIO (V/T) = 2.0075837425

TS minor type2: The transition state structure (Type 2) leading to the minor product in the click reaction between ABSACN Entry A and BnN_3.

Table S15. Calculated thermodynamic quantities and energy components for the transition state structures leading to the minor product (TS minor type2) in the cycloaddition reaction of ABSACN (R^1=R^2=H) with Benzyl azide.

	E (kcal/mol)	H (kcal/mol)	G (kcal/mol)	CV (cal/(mol·K))	CP (cal/(mol·K))	S (cal/(mol·K))
ELEC.	0	0	0	0	0	0
TRANS.	0.889	1.481	-11.487	2.981	4.968	43.495
ROT.	0.889	0.889	-9.666	2.981	2.981	35.402
VIB.	216.685	216.685	192.961	78.148	78.148	79.573
TOTAL	218.463	219.055	171.807	84.109	86.097	158.469
VIB. THERMAL CORRECTION E(T)-E(0) = H(T)-H(0) = 11815.357 cal/mol						
Imaginary Frequency: -337 cm⁻¹						

Note: All values are calculated at the B3LYP-D3(BJ)/6-31G(d) level. The presence of a single imaginary frequency confirms the nature of the transition state. ELEC., TRANS., ROT., and VIB. denote electronic, translational, rotational, and vibrational contributions, respectively.

ENERGY COMPONENTS□□Value (Hartree)
WAVEFUNCTION NORMALIZATION = 1.0000000000
ONE ELECTRON ENERGY = -6668.7779367301
TWO ELECTRON ENERGY = 2850.2984654473
NUCLEAR REPULSION ENERGY = 2338.8775756056
TOTAL ENERGY = -1479.6018956772
ELECTRON-ELECTRON POTENTIAL ENERGY = 2850.2984654473
NUCLEUS-ELECTRON POTENTIAL ENERGY = -8137.2403840717
NUCLEUS-NUCLEUS POTENTIAL ENERGY = 2338.8775756056
TOTAL POTENTIAL ENERGY = -2948.0643430188
TOTAL KINETIC ENERGY = 1468.4624473416
VIRIAL RATIO (V/T) = 2.0075857904

TS minor type3: The transition state structure (Type 3) leading to the minor product in the click reaction between ABSACN Entry A and BnN_3.

Table S16. Calculated thermodynamic quantities and energy components for the transition state structures leading to the minor product (TS minor type3) in the cycloaddition reaction of ABSACN (R^1=R^2=H) with Benzyl azide.

	E (kcal/mol)	H (kcal/mol)	G (kcal/mol)	CV (cal/(mol·K))	CP (cal/(mol·K))	S (cal/(mol·K))
ELEC.	0	0	0	0	0	0
TRANS.	0.889	1.481	-11.487	2.981	4.968	43.495
ROT.	0.889	0.889	-9.771	2.981	2.981	35.752
VIB.	216.584	216.584	192.044	78.272	78.272	82.307
TOTAL	218.361	218.954	170.786	84.234	86.221	161.554
VIB. THERMAL CORRECTION E(T)-E(0) = H(T)-H(0) = 11926.250 cal/mol						
Imaginary Frequency: -329 cm⁻¹						

Note: All values are calculated at the B3LYP-D3(BJ)/6-31G(d) level. The presence of a single imaginary frequency confirms the nature of the transition state. ELEC., TRANS., ROT., and VIB. denote electronic, translational, rotational, and vibrational contributions, respectively.

ENERGY COMPONENTS□□Value (Hartree)
WAVEFUNCTION NORMALIZATION = 1.0000000000
ONE ELECTRON ENERGY = -6508.6779365291
TWO ELECTRON ENERGY = 2770.3565400905
NUCLEAR REPULSION ENERGY = 2258.7220434814
TOTAL ENERGY = -1479.5993529572
ELECTRON-ELECTRON POTENTIAL ENERGY = 2770.3565400905
NUCLEUS-ELECTRON POTENTIAL ENERGY = -7977.1288927036
NUCLEUS-NUCLEUS POTENTIAL ENERGY = 2258.7220434814
TOTAL POTENTIAL ENERGY = -2948.0503091317
TOTAL KINETIC ENERGY = 1468.4509561745
VIRIAL RATIO (V/T) = 2.0075919436

GS2 major type1: The ground state structure (GS2) of the major product (Type 1) obtained from the click reaction between ABSACN Entry A and BnN_3

Table S17. Calculated thermodynamic quantities and energy components for the major product (GS2 major type1).

	E (kcal/mol)	H (kcal/mol)	G (kcal/mol)	CV (cal/(mol·K))	CP (cal/(mol·K))	S (cal/(mol·K))
ELEC.	0	0	0	0	0	0
TRANS.	0.889	1.481	-11.487	2.981	4.968	43.495
ROT.	0.889	0.889	-9.574	2.981	2.981	35.093
VIB.	219.664	219.664	198.397	74.398	74.398	71.331
TOTAL	221.441	222.034	177.336	80.36	82.347	149.918
VIB. THERMAL CORRECTION E(T)-E(0) = H(T)-H(0) = 10786.781 cal/mol						

Note: All values are calculated at the B3LYP-D3(BJ)/6-31G(d) level. ELEC., TRANS., ROT., and VIB. denote electronic, translational, rotational, and vibrational contributions, respectively.

ENERGY COMPONENTS□□Value (Hartree)
WAVEFUNCTION NORMALIZATION = 1.0000000000
ONE ELECTRON ENERGY = -6826.3127660847
TWO ELECTRON ENERGY = 2928.5569070541
NUCLEAR REPULSION ENERGY = 2418.0058345766
TOTAL ENERGY = -1479.7500244540
ELECTRON-ELECTRON POTENTIAL ENERGY = 2928.5569070541
NUCLEUS-ELECTRON POTENTIAL ENERGY = -8295.0221262081
NUCLEUS-NUCLEUS POTENTIAL ENERGY = 2418.0058345766
TOTAL POTENTIAL ENERGY = -2948.4593845773
TOTAL KINETIC ENERGY = 1468.7093601234
VIRIAL RATIO (V/T) = 2.0075172561

GS2 major type2: The ground state structure (GS2) of the major product (Type 2) obtained from the click reaction between ABSACN Entry A and BnN_3.

Table S18. Calculated thermodynamic quantities and energy components for the major product (GS2 major type2).

	E (kcal/mol)	H (kcal/mol)	G (kcal/mol)	CV (cal/(mol·K))	CP (cal/(mol·K))	S (cal/(mol·K))
ELEC.	0	0	0	0	0	0
TRANS.	0.889	1.481	-11.487	2.981	4.968	43.495
ROT.	0.889	0.889	-9.482	2.981	2.981	34.782
VIB.	219.691	219.691	199.284	74.613	74.613	68.443
TOTAL	221.468	222.061	178.316	80.575	82.562	146.72
VIB. THERMAL CORRECTION E(T)-E(0) = H(T)-H(0) = 10733.503 cal/mol						

Note: All values are calculated at the B3LYP-D3(BJ)/6-31G(d) level. ELEC., TRANS., ROT., and VIB. denote electronic, translational, rotational, and vibrational contributions, respectively.

ENERGY COMPONENTS□□Value (Hartree)
WAVEFUNCTION NORMALIZATION = 1.0000000000
ONE ELECTRON ENERGY = -6947.9157373566
TWO ELECTRON ENERGY = 2989.4236212030
NUCLEAR REPULSION ENERGY = 2478.7381749679
TOTAL ENERGY = -1479.7539411857
ELECTRON-ELECTRON POTENTIAL ENERGY = 2989.4236212030
NUCLEUS-ELECTRON POTENTIAL ENERGY = -8416.6348924536
NUCLEUS-NUCLEUS POTENTIAL ENERGY = 2478.7381749679
TOTAL POTENTIAL ENERGY = -2948.4730962827
TOTAL KINETIC ENERGY = 1468.7191550971
VIRIAL RATIO (V/T) = 2.0075132036

GS2 minor type1: The ground state structure (GS2) of the minor product (Type 1) obtained from the click reaction between ABSACN Entry A and BnN_3.

Table S19. Calculated thermodynamic quantities and energy components for the major product (GS2 minor type1).

	E (kcal/mol)	H (kcal/mol)	G (kcal/mol)	CV (cal/(mol·K))	CP (cal/(mol·K))	S (cal/(mol·K))
ELEC.	0	0	0	0	0	0
TRANS.	0.889	1.481	-11.487	2.981	4.968	43.495
ROT.	0.889	0.889	-9.579	2.981	2.981	35.108
VIB.	219.72	219.72	199.073	74.503	74.503	69.249
TOTAL	221.497	222.09	178.007	80.465	82.452	147.852
VIB. THERMAL CORRECTION E(T)-E(0) = H(T)-H(0) = 10746.933 cal/mol						

Note: All values are calculated at the B3LYP-D3(BJ)/6-31G(d) level. ELEC., TRANS., ROT., and VIB. denote electronic, translational, rotational, and vibrational contributions, respectively.

ENERGY COMPONENTS□□Value (Hartree)
WAVEFUNCTION NORMALIZATION = 1.0000000000
ONE ELECTRON ENERGY = -6828.4286924547
TWO ELECTRON ENERGY = 2929.6337087351
NUCLEAR REPULSION ENERGY = 2419.0470083866
TOTAL ENERGY = -1479.7479753331
ELECTRON-ELECTRON POTENTIAL ENERGY = 2929.6337087351
NUCLEUS-ELECTRON POTENTIAL ENERGY = -8297.1318596655
NUCLEUS-NUCLEUS POTENTIAL ENERGY = 2419.0470083866
TOTAL POTENTIAL ENERGY = -2948.4511425439
TOTAL KINETIC ENERGY = 1468.7031672108
VIRIAL RATIO (V/T) = 2.0075201091

GS2 minor type2: The ground state structure (GS2) of the minor product (Type 2) obtained from the click reaction between ABSACN Entry A and BnN_3.

Table S20. Calculated thermodynamic quantities and energy components for the major product (GS2 minor type2).

	E (kcal/mol)	H (kcal/mol)	G (kcal/mol)	CV (cal/(mol·K))	CP (cal/(mol·K))	S (cal/(mol·K))
ELEC.	0	0	0	0	0	0
TRANS.	0.889	1.481	-11.487	2.981	4.968	43.495
ROT.	0.889	0.889	-9.376	2.981	2.981	34.429
VIB.	219.673	219.673	199.498	74.425	74.425	67.666
TOTAL	221.45	222.042	178.635	80.387	82.374	145.591

VIB. THERMAL CORRECTION E(T)-E(0) = H(T)-H(0) = 10679.125 cal/mol

Note: All values are calculated at the B3LYP-D3(BJ)/6-31G(d) level. ELEC., TRANS., ROT., and VIB. denote electronic, translational, rotational, and vibrational contributions, respectively.

ENERGY COMPONENTS□□Value (Hartree)
WAVEFUNCTION NORMALIZATION = 1.0000000000
ONE ELECTRON ENERGY = -7107.5010125063
TWO ELECTRON ENERGY = 3069.1902522379
NUCLEAR REPULSION ENERGY = 2558.5536019597
TOTAL ENERGY = -1479.7571583086
ELECTRON-ELECTRON POTENTIAL ENERGY = 3069.1902522379
NUCLEUS-ELECTRON POTENTIAL ENERGY = -8576.2442153172
NUCLEUS-NUCLEUS POTENTIAL ENERGY = 2558.5536019597
TOTAL POTENTIAL ENERGY = -2948.5003611196
TOTAL KINETIC ENERGY = 1468.7432028110
VIRIAL RATIO (V/T) = 2.0074988980

Table S21/Table 2. **Summary of Reactivity, Frontier Orbital Energies, and Alkyne Bending Angles for N-substituted ABSACN Derivatives**.

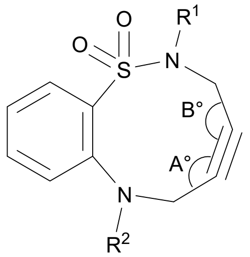

Entry A: R^1 = R^2 = H
Entry B: R^1= Boc, R^2 = H
Entry C: R^1= Boc, R^2 = Ac
Entry D: R^1 = H, R^2 = Ac
Entry E: R^1= Cbz, R^2 = H
Entry F: R^1 = Ts, R^2 = H

Entry	Reactivity	HOMO	HOMO-LUMO gap (eV)	Angle (A°)	Angle (B°)	Alkyne bending
A	0.43	-6.193	5.371	158.1	166.0	18.0
B	1.50	-6.123	5.301	155.1	162.0	21.5
C	0.74	-6.719	5.897	160.2	162.0	18.9
D	0.27	-6.797	5.972<br>(6.770)	162.9	164.4	16.4
E	2.10	-6.142	5.320	155.4	164.0	20.3
F	1.30	-6.234	5.412<br>(5.633)	157.4	167.0	17.8

The Reactivity values (relative rate constants) are experimentally determined. All HOMO and HOMO-LUMO gap energies are calculated in eV at the B3LYP-D3(BJ)/6-31G(d) level using the PCM solvation model with CH_2Cl_2 as the solvent. The values in parentheses for Entry D and F are included for the purpose of electronic comparison between HOMO and LUMO. The alkyne bending angle (degree) is determined using the formula: 180° - (Angle A + Angle B) / 2.
Benzyl azide, solvent=CH_2Cl_2: orbital energy HOMO -6.6804 eV, LUMO -0.8218 eV
Entry D (solvent=CH_2Cl_2: orbital energy HOMO -6.7974 eV, LUMO 0.0898 eV)
Entry F (solvent=CH_2Cl_2: orbital energy HOMO -6.2341 eV, LUMO -1.0476 eV)

Entry A ABSACN (R^1 =R^2 = H)**: The optimized structure of ABSACN Entry A under CH_2Cl_2 solvent conditions.**

Table S22. Calculated thermodynamic quantities and frontier orbital energy for the ABSACN Entry A(R^1=R^2=H) under PCM/CH_2Cl_2 solvent conditions.

	E (kcal/mol)	H (kcal/mol)	G (kcal/mol)	CV (cal/(mol·K))	CP (cal/(mol·K))	S (cal/(mol·K))
ELEC.	0	0	0	0	0	0
TRANS.	0.889	1.481	-11.07	2.981	4.968	42.096
ROT.	0.889	0.889	-8.576	2.981	2.981	31.745
VIB.	126.868	126.868	116.509	44.149	44.149	34.745
TOTAL	128.646	129.238	96.864	50.111	52.098	108.586

VIB. THERMAL CORRECTION E(T)-E(0) = H(T)-H(0) = 6082.743 cal/mol

Note: All thermodynamic properties were calculated at 298.15 K and 1.0 atm. All values are calculated at the B3LYP-D3(BJ)/6-31G(d) level using the PCM solvation model (solvent=CH_2Cl_2). ELEC., TRANS., ROT., and VIB. denote electronic, translational, rotational, and vibrational contributions, respectively. The HOMO energy is -6.1933 eV.

RESULTS OF PCM CALCULATION
FREE ENERGY IN SOLVENT = <PSI| H(0)+V/2 |PSI> = -1044.7054835993 a.u.
INTERNAL ENERGY IN SOLVENT = <PSI| H(0) |PSI> = -1044.6904587527 a.u.
DELTA INTERNAL ENERGY = <D-PSI| H(0) |D-PSI> = 0.0000000000 a.u.
ELECTROSTATIC INTERACTION = -0.0150248466 a.u.
PIEROTTI CAVITATION ENERGY = 0.0000000000 a.u.
DISPERSION FREE ENERGY = 0.0000000000 a.u.
REPULSION FREE ENERGY = 0.0000000000 a.u.
TOTAL INTERACTION (DELTA + ES + CAV + DISP + REP) = -0.0150248466 a.u.
TOTAL FREE ENERGY IN SOLVENT = -1044.7054835993 a.u.

FREE ENERGY IN SOLVENT = -655562.66 kcal/mol
INTERNAL ENERGY IN SOLVENT = -655553.23 kcal/mol
DELTA INTERNAL ENERGY = 0.00 kcal/mol
ELECTROSTATIC INTERACTION = -9.43 kcal/mol
PIEROTTI CAVITATION ENERGY = 0.00 kcal/mol
DISPERSION FREE ENERGY = 0.00 kcal/mol
REPULSION FREE ENERGY = 0.00 kcal/mol
TOTAL INTERACTION = -9.43 kcal/mol
TOTAL FREE ENERGY IN SOLVENT = -655562.66 kcal/mol

Entry B ABSACN (R^1= Boc, R^2 = H): **The optimized structure of ABSACN Entry B under CH_2Cl_2 solvent conditions.**

Table S23. Calculated thermodynamic quantities and frontier orbital energy for the ABSACN Entry B (R^1=Boc, R^2=H) under PCM/CH_2Cl_2 solvent conditions.

	E (kcal/mol)	H (kcal/mol)	G (kcal/mol)	CV (cal/(mol·K))	CP (cal/(mol·K))	S (cal/(mol·K))
ELEC.	0	0	0	0	0	0
TRANS.	0.889	1.481	-11.4	2.981	4.968	43.204
ROT.	0.889	0.889	-9.241	2.981	2.981	33.976
VIB.	212.214	212.214	191.385	74.965	74.965	69.859
TOTAL	213.991	214.583	170.744	80.927	82.914	147.039
VIB. THERMAL CORRECTION E(T)-E(0) = H(T)-H(0) = 11162.631 cal/mol						

Note: All thermodynamic properties were calculated at 298.15 K and 1.0 atm. All values are calculated at the B3LYP-D3(BJ)/6-31G(d) level using the PCM solvation model (solvent=CH_2Cl_2). ELEC., TRANS., ROT., and VIB. denote electronic, translational, rotational, and vibrational contributions, respectively. The HOMO energy is -6.1226 eV.

RESULTS OF PCM CALCULATION
FREE ENERGY IN SOLVENT = <PSI| H(0)+V/2 |PSI> = -1390.3665107822 a.u.
INTERNAL ENERGY IN SOLVENT = <PSI| H(0) |PSI> = -1390.3523902516 a.u.
DELTA INTERNAL ENERGY = <D-PSI| H(0) |D-PSI> = 0.0000000000 a.u.
ELECTROSTATIC INTERACTION = -0.0141205306 a.u.
PIEROTTI CAVITATION ENERGY = 0.0000000000 a.u.
DISPERSION FREE ENERGY = 0.0000000000 a.u.
REPULSION FREE ENERGY = 0.0000000000 a.u.
TOTAL INTERACTION (DELTA + ES + CAV + DISP + REP) = -0.0141205306 a.u.
TOTAL FREE ENERGY IN SOLVENT = -1390.3665107822 a.u.

FREE ENERGY IN SOLVENT = -872468.25 kcal/mol
INTERNAL ENERGY IN SOLVENT = -872459.39 kcal/mol
DELTA INTERNAL ENERGY = 0.00 kcal/mol
ELECTROSTATIC INTERACTION = -8.86 kcal/mol
PIEROTTI CAVITATION ENERGY = 0.00 kcal/mol
DISPERSION FREE ENERGY = 0.00 kcal/mol
REPULSION FREE ENERGY = 0.00 kcal/mol

TOTAL INTERACTION = -8.86 kcal/mol
TOTAL FREE ENERGY IN SOLVENT = -872468.25 kcal/mol

Entry C□ABSACN (R^1= Boc, R^2 = Ac)：**The optimized structure of ABSACN Entry C under CH_2Cl_2 solvent conditions.**

Table S24. Calculated thermodynamic quantities and frontier orbital energy for the ABSACN Entry C (R^1=Boc, R^2=Ac) under PCM/CH_2Cl_2 solvent conditions.

	E (kcal/mol)	H (kcal/mol)	G (kcal/mol)	CV (cal/(mol·K))	CP (cal/(mol·K))	S (cal/(mol·K))
ELEC.	0	0	0	0	0	0
TRANS.	0.889	1.481	-11.509	2.981	4.968	43.57
ROT.	0.889	0.889	-9.477	2.981	2.981	34.766
VIB.	237.638	237.638	212.133	87.205	87.205	85.544
TOTAL	239.415	240.008	191.147	93.167	95.154	163.88

Note: All thermodynamic properties were calculated at 298.15 K and 1.0 atm. All values are calculated at the B3LYP-D3(BJ)/6-31G(d) level using the PCM solvation model (solvent=CH_2Cl_2). ELEC., TRANS., ROT., and VIB. denote electronic, translational, rotational, and vibrational contributions, respectively. The HOMO energy is -6.7185 eV.

RESULTS OF PCM CALCULATION
FREE ENERGY IN SOLVENT = <PSI| H(0)+V/2 |PSI> = -1542.9563293273 a.u.
INTERNAL ENERGY IN SOLVENT = <PSI| H(0) |PSI> = -1542.9358607611 a.u.
DELTA INTERNAL ENERGY = <D-PSI| H(0) |D-PSI> = 0.0000000000 a.u.
ELECTROSTATIC INTERACTION = -0.0204685662 a.u.
PIEROTTI CAVITATION ENERGY = 0.0000000000 a.u.
DISPERSION FREE ENERGY = 0.0000000000 a.u.
REPULSION FREE ENERGY = 0.0000000000 a.u.
TOTAL INTERACTION (DELTA + ES + CAV + DISP + REP) = -0.0204685662 a.u.
TOTAL FREE ENERGY IN SOLVENT = -1542.9563293273 a.u.

FREE ENERGY IN SOLVENT = -968219.82 kcal/mol
INTERNAL ENERGY IN SOLVENT = -968206.97 kcal/mol
DELTA INTERNAL ENERGY = 0.00 kcal/mol
ELECTROSTATIC INTERACTION = -12.84 kcal/mol
PIEROTTI CAVITATION ENERGY = 0.00 kcal/mol
DISPERSION FREE ENERGY = 0.00 kcal/mol
REPULSION FREE ENERGY = 0.00 kcal/mol
TOTAL INTERACTION = -12.84 kcal/mol
TOTAL FREE ENERGY IN SOLVENT = -968219.82 kcal/mol

Entry D□ABSACN (R^1= H, R^2 = Ac)：**The optimized structure of ABSACN Entry D under CH_2Cl_2 solvent conditions.**

Table S25. Calculated thermodynamic quantities and frontier orbital energy for the ABSACN Entry D (R^1=H, R^2=Ac) under PCM/CH_2Cl_2 solvent conditions.

	E (kcal/mol)	H (kcal/mol)	G (kcal/mol)	CV (cal/(mol·K))	CP (cal/(mol·K))	S (cal/(mol·K))
ELEC.	0	0	0	0	0	0
TRANS.	0.889	1.481	-11.224	2.981	4.968	42.612
ROT.	0.889	0.889	-8.874	2.981	2.981	32.744
VIB.	152.546	152.546	137.994	55.95	55.95	48.808
TOTAL	154.324	154.916	117.897	61.912	63.899	124.164
VIB. THERMAL CORRECTION E(T)-E(0) = H(T)-H(0) = 8146.582 cal/mol						

Note: All thermodynamic properties were calculated at 298.15 K and 1.0 atm. All values are calculated at the B3LYP-D3(BJ)/6-31G(d) level using the PCM solvation model (solvent=CH_2Cl_2). ELEC., TRANS., ROT., and VIB. denote electronic, translational, rotational, and vibrational contributions, respectively. The HOMO energy is -6.7974 eV, and the

LUMO energy is 0.0898 eV.

RESULTS OF PCM CALCULATION
FREE ENERGY IN SOLVENT = <PSI| H(0)+V/2 |PSI> = -1197.2966408345 a.u.
INTERNAL ENERGY IN SOLVENT = <PSI| H(0) |PSI> = -1197.2751774128 a.u.
DELTA INTERNAL ENERGY = <D-PSI| H(0) |D-PSI> = 0.0000000000 a.u.
ELECTROSTATIC INTERACTION = -0.0214634216 a.u.
PIEROTTI CAVITATION ENERGY = 0.0000000000 a.u.
DISPERSION FREE ENERGY = 0.0000000000 a.u.
REPULSION FREE ENERGY = 0.0000000000 a.u.
TOTAL INTERACTION (DELTA + ES + CAV + DISP + REP) = -0.0214634216 a.u.
TOTAL FREE ENERGY IN SOLVENT = -1197.2966408345 a.u.

FREE ENERGY IN SOLVENT = -751315.07 kcal/mol
INTERNAL ENERGY IN SOLVENT = -751301.60 kcal/mol
DELTA INTERNAL ENERGY = 0.00 kcal/mol
ELECTROSTATIC INTERACTION = -13.47 kcal/mol
PIEROTTI CAVITATION ENERGY = 0.00 kcal/mol
DISPERSION FREE ENERGY = 0.00 kcal/mol
REPULSION FREE ENERGY = 0.00 kcal/mol
TOTAL INTERACTION = -13.47 kcal/mol
TOTAL FREE ENERGY IN SOLVENT = -751315.07 kcal/mol

Entry E ABSACN (R^1= Cbz, R^2 = H)：**The optimized structure of ABSACN Entry E under CH_2Cl_2 solvent conditions.**

Table S26. Calculated thermodynamic quantities and frontier orbital energy for the ABSACN Entry E (R^1=Cbz, R^2=H) under PCM/CH_2Cl_2 solvent conditions.

	E (kcal/mol)	H (kcal/mol)	G (kcal/mol)	CV (cal/(mol·K))	CP (cal/(mol·K))	S (cal/(mol·K))
ELEC.	0	0	0	0	0	0
TRANS.	0.889	1.481	-11.489	2.981	4.968	43.503
ROT.	0.889	0.889	-9.573	2.981	2.981	35.089
VIB.	210.727	210.727	188.697	76.643	76.643	73.888
TOTAL	212.505	213.097	167.635	82.604	84.591	152.48
VIB. THERMAL CORRECTION E(T)-E(0) = H(T)-H(0) = 11359.987 cal/mol						

Note: All thermodynamic properties were calculated at 298.15 K and 1.0 atm. All values are calculated at the B3LYP-D3(BJ)/6-31G(d) level using the PCM solvation model (solvent=CH_2Cl_2). ELEC., TRANS., ROT., and VIB. denote electronic, translational, rotational, and vibrational contributions, respectively. The HOMO energy is -6.1416 eV.

RESULTS OF PCM CALCULATION
FREE ENERGY IN SOLVENT = <PSI| H(0)+V/2 |PSI> = -1503.4164803067 a.u.
INTERNAL ENERGY IN SOLVENT = <PSI| H(0) |PSI> = -1503.4005544703 a.u.
DELTA INTERNAL ENERGY = <D-PSI| H(0) |D-PSI> = 0.0000000000 a.u.
ELECTROSTATIC INTERACTION = -0.0159258363 a.u.
PIEROTTI CAVITATION ENERGY = 0.0000000000 a.u.
DISPERSION FREE ENERGY = 0.0000000000 a.u.
REPULSION FREE ENERGY = 0.0000000000 a.u.
TOTAL INTERACTION (DELTA + ES + CAV + DISP + REP) = -0.0159258363 a.u.
TOTAL FREE ENERGY IN SOLVENT = -1503.4164803067 a.u.

FREE ENERGY IN SOLVENT = -943408.19 kcal/mol
INTERNAL ENERGY IN SOLVENT = -943398.19 kcal/mol
DELTA INTERNAL ENERGY = 0.00 kcal/mol
ELECTROSTATIC INTERACTION = -9.99 kcal/mol
PIEROTTI CAVITATION ENERGY = 0.00 kcal/mol
DISPERSION FREE ENERGY = 0.00 kcal/mol
REPULSION FREE ENERGY = 0.00 kcal/mol
TOTAL INTERACTION = -9.99 kcal/mol
TOTAL FREE ENERGY IN SOLVENT = -943408.19 kcal/mol

Entry F ABSACN (R^1= Ts, R^2 = H)：**The optimized structure of ABSACN Entry F under CH_2Cl_2 solvent**

conditions.

Table S27. Calculated thermodynamic quantities and frontier orbital energy for the ABSACN Entry E (R^1=Ts, R^2=H) under PCM/CH_2Cl_2 solvent conditions.

	E (kcal/mol)	H (kcal/mol)	G (kcal/mol)	CV (cal/(mol·K))	CP (cal/(mol·K))	S (cal/(mol·K))
ELEC.	0	0	0	0	0	0
TRANS.	0.889	1.481	-11.538	2.981	4.968	43.666
ROT.	0.889	0.889	-9.583	2.981	2.981	35.122
VIB.	207.495	207.495	184.099	80.783	80.783	78.471
TOTAL	209.273	209.865	162.978	86.745	88.732	157.259
VIB. THERMAL CORRECTION E(T)-E(0) = H(T)-H(0) = 12140.795 cal/mol						

Note: All thermodynamic properties were calculated at 298.15 K and 1.0 atm. All values are calculated at the B3LYP-D3(BJ)/6-31G(d) level using the PCM solvation model (solvent=CH_2Cl_2). ELEC., TRANS., ROT., and VIB. denote electronic, translational, rotational, and vibrational contributions, respectively. The HOMO energy is -6.2341 eV, and the LUMO energy is -1.04760 eV.

RESULTS OF PCM CALCULATION
FREE ENERGY IN SOLVENT = <PSI| H(0)+V/2 |PSI> = -1863.3883458528 a.u.
INTERNAL ENERGY IN SOLVENT = <PSI| H(0) |PSI> = -1863.3715713658 a.u.
DELTA INTERNAL ENERGY = <D-PSI| H(0) |D-PSI> = 0.0000000000 a.u.
ELECTROSTATIC INTERACTION = -0.0167744870 a.u.
PIEROTTI CAVITATION ENERGY = 0.0000000000 a.u.
DISPERSION FREE ENERGY = 0.0000000000 a.u.
REPULSION FREE ENERGY = 0.0000000000 a.u.
TOTAL INTERACTION (DELTA + ES + CAV + DISP + REP) = -0.0167744870 a.u.
TOTAL FREE ENERGY IN SOLVENT = -1863.3883458528 a.u.

FREE ENERGY IN SOLVENT = -1169293.97 kcal/mol
INTERNAL ENERGY IN SOLVENT = -1169283.44 kcal/mol
DELTA INTERNAL ENERGY = 0.00 kcal/mol
ELECTROSTATIC INTERACTION = -10.53 kcal/mol
PIEROTTI CAVITATION ENERGY = 0.00 kcal/mol
DISPERSION FREE ENERGY = 0.00 kcal/mol
REPULSION FREE ENERGY = 0.00 kcal/mol
TOTAL INTERACTION = -10.53 kcal/mol
TOTAL FREE ENERGY IN SOLVENT = -1169293.97 kcal/mol

Benzyl azide□ solvent=CH_2Cl_2: The optimized structure of Benzyl azide under CH_2Cl_2 solvent conditions.

Table S28. Calculated thermodynamic quantities and frontier orbital energy for the Benzyl azide (BnN_3) under PCM/CH_2Cl_2 solvent conditions.

	E (kcal/mol)	H (kcal/mol)	G (kcal/mol)	CV (cal/(mol·K))	CP (cal/(mol·K))	S (cal/(mol·K))
ELEC.	0	0	0	0	0	0
TRANS.	0.889	1.481	-10.615	2.981	4.968	40.569
ROT.	0.889	0.889	-7.896	2.981	2.981	29.465
VIB.	86.747	86.747	80.012	24.914	24.914	22.589
TOTAL	88.525	89.117	61.502	30.875	32.862	92.624
VIB. THERMAL CORRECTION E(T)-E(0) = H(T)-H(0) = 3490.118 cal/mol						

Note: All thermodynamic properties were calculated at 298.15 K and 1.0 atm. All values are calculated at the B3LYP-D3(BJ)/6-31G(d) level using the PCM solvation model (solvent=CH_2Cl_2). ELEC., TRANS., ROT., and VIB. denote electronic, translational, rotational, and vibrational contributions, respectively. The HOMO energy is -6.6804 eV, and the LUMO energy is -0.8218 eV.

RESULTS OF PCM CALCULATION
FREE ENERGY IN SOLVENT = <PSI| H(0)+V/2 |PSI> = -434.9230101714 a.u.
INTERNAL ENERGY IN SOLVENT = <PSI| H(0) |PSI> = -434.9170131476 a.u.

```
DELTA INTERNAL ENERGY =    <D-PSI| H(0) |D-PSI>          =            0.0000000000 a.u.
ELECTROSTATIC INTERACTION                                 =            -0.0059970238 a.u.
PIEROTTI CAVITATION ENERGY                                =            0.0000000000 a.u.
DISPERSION FREE ENERGY                                    =            0.0000000000 a.u.
REPULSION FREE ENERGY                                     =            0.0000000000 a.u.
TOTAL INTERACTION (DELTA + ES + CAV + DISP + REP)         =            -0.0059970238 a.u.
TOTAL FREE ENERGY IN SOLVENT                              =         -434.9230101714 a.u.

FREE ENERGY IN SOLVENT           =       -272918.34 kcal/mol
INTERNAL ENERGY IN SOLVENT       =       -272914.58 kcal/mol
DELTA INTERNAL ENERGY            =             0.00 kcal/mol
ELECTROSTATIC INTERACTION        =            -3.76 kcal/mol
PIEROTTI CAVITATION ENERGY       =             0.00 kcal/mol
DISPERSION FREE ENERGY           =             0.00 kcal/mol
REPULSION FREE ENERGY            =             0.00 kcal/mol
TOTAL INTERACTION                =            -3.76 kcal/mol
TOTAL FREE ENERGY IN SOLVENT =           -272918.34 kcal/mol
```

Transition State Energetics for Substituted ABSACN Derivatives (Table 4)

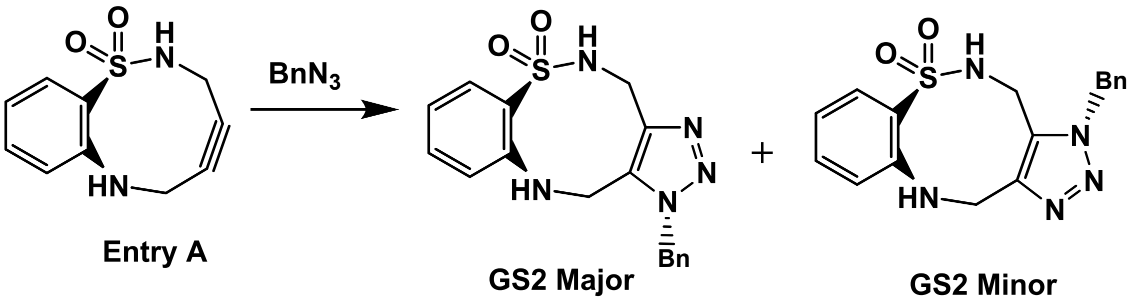

Figure S6. Formation of regioisomers in the strain-promoted cycloaddition of ABSACN Entry A (R^1=R^2=H) and Benzyl azide with PCM calculations applied.

Table S29. Calculated Free Energies for the Cycloaddition Reaction Pathway of ABSACN Entry A with Benzyl Azide in CH_2Cl_2 solvent.

Structure	Imaginary Frequency (cm^{-1})	Total Free Energy in Solvent (au)	Total Free Energy in Solvent (kcal/mol) (Relative to GS1)
GS1	N/A	-1479.62849	0
TS major1	-360	-1479.61953	5.62234
TS major2	-315	-1479.61734	6.99910
TS major3	-329	-1479.61717	7.10755
TS minor1	-360	-1479.61871	6.13757
TS minor2	-366	-1479.61803	6.56823
TS minor3	-350	-1479.61475	8.62675
GS2 Major	N/A	-1479.76905	--88.19973
GS2 Minor	N/A	-1479.76729	-87.09711

Note: All free energies were calculated at the B3LYP-D3(BJ)/6-31G(d) level using the PCM solvation model (solvent=CH_2Cl_2) at 298.15 K and 1.0 atm. Energies are reported as Total Free Energy in Solvent (au) and relative free energies (kcal/mol) with respect to the ground state of the reactants (GS1). The single imaginary frequency (cm^{-1}) confirms the nature of the transition states (TS). GS1 (Reactants) Total Free Energy in Solvent: -1479.62849 au.

GS1
Entry A solvent=CH_2Cl_2 + Benzyl azide□solvent=CH_2Cl_2

The optimized ground state structure (GS1) representing the total energy of the reactants (each optimized ABSACN and optimized BnN_3) under dichloromethane (CH_2Cl_2) solvent conditions.

Entry A TS major1: The transition state structure leading to the major product in the click reaction between ABSACN Entry A and BnN_3 with CH_2Cl_2 solvent.

Table S30. Calculated thermodynamic quantities for TS major1 in the cycloaddition reaction of ABSACN Entry A with Benzyl azide under PCM/CH_2Cl_2 solvent conditions.

	E (kcal/mol)	H (kcal/mol)	G (kcal/mol)	CV (cal/(mol·K))	CP (cal/(mol·K))	S (cal/(mol·K))
ELEC.	0	0	0	0	0	0
TRANS.	0.889	1.481	-11.487	2.981	4.968	43.495
ROT.	0.889	0.889	-9.647	2.981	2.981	35.339
VIB.	216.66	216.66	193.597	78.141	78.141	77.354
TOTAL	218.438	219.03	172.463	84.102	86.09	156.188
VIB. THERMAL CORRECTION E(T)-E(0) = H(T)-H(0) = 11738.389 cal/mol						
Imaginary Frequency: -360 cm^{-1}						

Note: All thermodynamic properties were calculated at 298.15 K and 1.0 atm. All values are calculated at the B3LYP-D3(BJ)/6-31G(d) level using the PCM solvation model (solvent=CH_2Cl_2). The presence of a single imaginary frequency (-360 cm^{-1}) confirms the nature of the transition state. ELEC., TRANS., ROT., and VIB. denote electronic, translational, rotational, and vibrational contributions, respectively.

RESULTS OF PCM CALCULATION
FREE ENERGY IN SOLVENT = <PSI| H(0)+V/2 |PSI> = -1479.6195340169 a.u.
INTERNAL ENERGY IN SOLVENT = <PSI| H(0) |PSI> = -1479.5997256380 a.u.
DELTA INTERNAL ENERGY = <D-PSI| H(0) |D-PSI> = 0.0000000000 a.u.
ELECTROSTATIC INTERACTION = -0.0198083789 a.u.
PIEROTTI CAVITATION ENERGY = 0.0000000000 a.u.
DISPERSION FREE ENERGY = 0.0000000000 a.u.
REPULSION FREE ENERGY = 0.0000000000 a.u.
TOTAL INTERACTION (DELTA + ES + CAV + DISP + REP) = -0.0198083789 a.u.
TOTAL FREE ENERGY IN SOLVENT = -1479.6195340169 a.u.

FREE ENERGY IN SOLVENT = -928475.37 kcal/mol
INTERNAL ENERGY IN SOLVENT = -928462.94 kcal/mol
DELTA INTERNAL ENERGY = 0.00 kcal/mol
ELECTROSTATIC INTERACTION = -12.43 kcal/mol
PIEROTTI CAVITATION ENERGY = 0.00 kcal/mol
DISPERSION FREE ENERGY = 0.00 kcal/mol
REPULSION FREE ENERGY = 0.00 kcal/mol
TOTAL INTERACTION = -12.43 kcal/mol
TOTAL FREE ENERGY IN SOLVENT = -928475.37 kcal/mol

Entry A TS major2: The transition state structure leading to the major product in the click reaction between ABSACN Entry A and BnN_3 with CH_2Cl_2 solvent.

Table S31. Calculated thermodynamic quantities for TS major2 in the cycloaddition reaction of ABSACN Entry A with Benzyl azide under PCM/CH_2Cl_2 solvent conditions.

	E (kcal/mol)	H (kcal/mol)	G (kcal/mol)	CV (cal/(mol·K))	CP (cal/(mol·K))	S (cal/(mol·K))
ELEC.	0	0	0	0	0	0
TRANS.	0.889	1.481	-11.487	2.981	4.968	43.495
ROT.	0.889	0.889	-9.751	2.981	2.981	35.685
VIB.	216.751	216.751	193.594	78.09	78.09	77.668
TOTAL	218.528	219.121	172.356	84.051	86.039	156.848

VIB. THERMAL CORRECTION E(T)-E(0) = H(T)-H(0) = 11769.134 cal/mol	
Imaginary Frequency: -315 cm^{-1}	

Note: All thermodynamic properties were calculated at 298.15 K and 1.0 atm. All values are calculated at the B3LYP-D3(BJ)/6-31G(d) level using the PCM solvation model (solvent=CH_2Cl_2). The presence of a single imaginary frequency (-315 cm^{-1}) confirms the nature of the transition state. ELEC., TRANS., ROT., and VIB. denote electronic, translational, rotational, and vibrational contributions, respectively.

RESULTS OF PCM CALCULATION
FREE ENERGY IN SOLVENT = <PSI| H(0)+V/2 |PSI> = -1479.6173408154 a.u.
INTERNAL ENERGY IN SOLVENT = <PSI| H(0) |PSI> = -1479.5971028530 a.u.
DELTA INTERNAL ENERGY = <D-PSI| H(0) |D-PSI> = 0.0000000000 a.u.
ELECTROSTATIC INTERACTION = -0.0202379624 a.u.
PIEROTTI CAVITATION ENERGY = 0.0000000000 a.u.
DISPERSION FREE ENERGY = 0.0000000000 a.u.
REPULSION FREE ENERGY = 0.0000000000 a.u.
TOTAL INTERACTION (DELTA + ES + CAV + DISP + REP) = -0.0202379624 a.u.
TOTAL FREE ENERGY IN SOLVENT = -1479.6173408154 a.u.

FREE ENERGY IN SOLVENT = -928474.00 kcal/mol
INTERNAL ENERGY IN SOLVENT = -928461.30 kcal/mol
DELTA INTERNAL ENERGY = 0.00 kcal/mol
ELECTROSTATIC INTERACTION = -12.70 kcal/mol
PIEROTTI CAVITATION ENERGY = 0.00 kcal/mol
DISPERSION FREE ENERGY = 0.00 kcal/mol
REPULSION FREE ENERGY = 0.00 kcal/mol
TOTAL INTERACTION = -12.70 kcal/mol
TOTAL FREE ENERGY IN SOLVENT = -928474.00 kcal/mol

Entry A TS major3: The transition state structure leading to the major product in the click reaction between ABSACN Entry A and BnN_3 with CH_2Cl_2 solvent.

Table S32. Calculated thermodynamic quantities for TS major3 in the cycloaddition reaction of ABSACN Entry A with Benzyl azide under PCM/CH_2Cl_2 solvent conditions.

	E (kcal/mol)	H (kcal/mol)	G (kcal/mol)	CV (cal/(mol·K))	CP (cal/(mol·K))	S (cal/(mol·K))
ELEC.	0	0	0	0	0	0
TRANS.	0.889	1.481	-11.487	2.981	4.968	43.495
ROT.	0.889	0.889	-9.759	2.981	2.981	35.713
VIB.	216.689	216.689	193.266	78.178	78.178	78.561
TOTAL	218.466	219.059	172.02	84.14	86.127	157.769
VIB. THERMAL CORRECTION E(T)-E(0) = H(T)-H(0) = 11803.755 cal/mol						
Imaginary Frequency: -329 cm^{-1}						

Note: All thermodynamic properties were calculated at 298.15 K and 1.0 atm. All values are calculated at the B3LYP-D3(BJ)/6-31G(d) level using the PCM solvation model (solvent=CH_2Cl_2). The presence of a single imaginary frequency (-329 cm^{-1}) confirms the nature of the transition state. ELEC., TRANS., ROT., and VIB. denote electronic, translational, rotational, and vibrational contributions, respectively.

RESULTS OF PCM CALCULATION
FREE ENERGY IN SOLVENT = <PSI| H(0)+V/2 |PSI> = -1479.6171671784 a.u.
INTERNAL ENERGY IN SOLVENT = <PSI| H(0) |PSI> = -1479.5971469542 a.u.
DELTA INTERNAL ENERGY = <D-PSI| H(0) |D-PSI> = 0.0000000000 a.u.
ELECTROSTATIC INTERACTION = -0.0200202242 a.u.
PIEROTTI CAVITATION ENERGY = 0.0000000000 a.u.
DISPERSION FREE ENERGY = 0.0000000000 a.u.
REPULSION FREE ENERGY = 0.0000000000 a.u.
TOTAL INTERACTION (DELTA + ES + CAV + DISP + REP) = -0.0200202242 a.u.
TOTAL FREE ENERGY IN SOLVENT = -1479.6171671784 a.u.

FREE ENERGY IN SOLVENT = -928473.89 kcal/mol
INTERNAL ENERGY IN SOLVENT = -928461.33 kcal/mol
DELTA INTERNAL ENERGY = 0.00 kcal/mol
ELECTROSTATIC INTERACTION = -12.56 kcal/mol
PIEROTTI CAVITATION ENERGY = 0.00 kcal/mol
DISPERSION FREE ENERGY = 0.00 kcal/mol
REPULSION FREE ENERGY = 0.00 kcal/mol
TOTAL INTERACTION = -12.56 kcal/mol
TOTAL FREE ENERGY IN SOLVENT = -928473.89 kcal/mol

Entry A TS minor1: The transition state structure leading to the minor product in the click reaction between ABSACN Entry A and BnN_3 with CH_2Cl_2 solvent.

Table S33. Calculated thermodynamic quantities for TS minor1 in the cycloaddition reaction of ABSACN Entry A with Benzyl azide under PCM/CH_2Cl_2 solvent conditions.

	E (kcal/mol)	H (kcal/mol)	G (kcal/mol)	CV (cal/(mol·K))	CP (cal/(mol·K))	S (cal/(mol·K))
ELEC.	0	0	0	0	0	0
TRANS.	0.889	1.481	-11.487	2.981	4.968	43.495
ROT.	0.889	0.889	-9.668	2.981	2.981	35.409
VIB.	216.508	216.508	192.881	78.23	78.23	79.244
TOTAL	218.285	218.878	171.726	84.192	86.179	158.148
VIB. THERMAL CORRECTION E(T)-E(0) = H(T)-H(0) = 11789.874 cal/mol						
Imaginary Frequency: -360 cm^{-1}						

Note: All thermodynamic properties were calculated at 298.15 K and 1.0 atm. All values are calculated at the B3LYP-D3(BJ)/6-31G(d) level using the PCM solvation model (solvent=CH_2Cl_2). The presence of a single imaginary frequency (-360 cm^{-1}) confirms the nature of the transition state. ELEC., TRANS., ROT., and VIB. denote electronic, translational, rotational, and vibrational contributions, respectively.

RESULTS OF PCM CALCULATION
FREE ENERGY IN SOLVENT = <PSI| H(0)+V/2 |PSI> = -1479.6187129432 a.u.
INTERNAL ENERGY IN SOLVENT = <PSI| H(0) |PSI> = -1479.5996295799 a.u.
DELTA INTERNAL ENERGY = <D-PSI| H(0) |D-PSI> = 0.0000000000 a.u.
ELECTROSTATIC INTERACTION = -0.0190833633 a.u.
PIEROTTI CAVITATION ENERGY = 0.0000000000 a.u.
DISPERSION FREE ENERGY = 0.0000000000 a.u.
REPULSION FREE ENERGY = 0.0000000000 a.u.
TOTAL INTERACTION (DELTA + ES + CAV + DISP + REP) = -0.0190833633 a.u.
TOTAL FREE ENERGY IN SOLVENT = -1479.6187129432 a.u.

FREE ENERGY IN SOLVENT = -928474.86 kcal/mol
INTERNAL ENERGY IN SOLVENT = -928462.88 kcal/mol
DELTA INTERNAL ENERGY = 0.00 kcal/mol
ELECTROSTATIC INTERACTION = -11.97 kcal/mol
PIEROTTI CAVITATION ENERGY = 0.00 kcal/mol
DISPERSION FREE ENERGY = 0.00 kcal/mol
REPULSION FREE ENERGY = 0.00 kcal/mol
TOTAL INTERACTION = -11.97 kcal/mol
TOTAL FREE ENERGY IN SOLVENT = -928474.86 kcal/mol

Entry A TS minor2: The transition state structure leading to the minor product in the click reaction between ABSACN Entry A and BnN_3 with CH_2Cl_2 solvent.

Table S34. Calculated thermodynamic quantities for TS minor2 in the cycloaddition reaction of ABSACN Entry A with Benzyl azide under PCM/CH_2Cl_2 solvent conditions.

	E (kcal/mol)	H (kcal/mol)	G (kcal/mol)	CV (cal/(mol·K))	CP (cal/(mol·K))	S (cal/(mol·K))
ELEC.	0	0	0	0	0	0

TRANS.	0.889	1.481	-11.487	2.981	4.968	43.495
ROT.	0.889	0.889	-9.622	2.981	2.981	35.252
VIB.	216.301	216.301	192.254	78.655	78.655	80.656
TOTAL	218.079	218.671	171.145	84.617	86.604	159.403
VIB. THERMAL CORRECTION E(T)-E(0) = H(T)-H(0) = 11925.534 cal/mol						
Imaginary Frequency: -366 cm^{-1}						

Note: All thermodynamic properties were calculated at 298.15 K and 1.0 atm. All values are calculated at the B3LYP-D3(BJ)/6-31G(d) level using the PCM solvation model (solvent=CH_2Cl_2). The presence of a single imaginary frequency (-366 cm^{-1}) confirms the nature of the transition state. ELEC., TRANS., ROT., and VIB. denote electronic, translational, rotational, and vibrational contributions, respectively.

RESULTS OF PCM CALCULATION
FREE ENERGY IN SOLVENT = <PSI| H(0)+V/2 |PSI> = -1479.6180266313 a.u.
INTERNAL ENERGY IN SOLVENT = <PSI| H(0) |PSI> = -1479.5988166708 a.u.
DELTA INTERNAL ENERGY = <D-PSI| H(0) |D-PSI> = 0.0000000000 a.u.
ELECTROSTATIC INTERACTION = -0.0192099604 a.u.
PIEROTTI CAVITATION ENERGY = 0.0000000000 a.u.
DISPERSION FREE ENERGY = 0.0000000000 a.u.
REPULSION FREE ENERGY = 0.0000000000 a.u.
TOTAL INTERACTION (DELTA + ES + CAV + DISP + REP) = -0.0192099604 a.u.
TOTAL FREE ENERGY IN SOLVENT = -1479.6180266313 a.u.

FREE ENERGY IN SOLVENT = -928474.43 kcal/mol
INTERNAL ENERGY IN SOLVENT = -928462.37 kcal/mol
DELTA INTERNAL ENERGY = 0.00 kcal/mol
ELECTROSTATIC INTERACTION = -12.05 kcal/mol
PIEROTTI CAVITATION ENERGY = 0.00 kcal/mol
DISPERSION FREE ENERGY = 0.00 kcal/mol
REPULSION FREE ENERGY = 0.00 kcal/mol
TOTAL INTERACTION = -12.05 kcal/mol
TOTAL FREE ENERGY IN SOLVENT = -928474.43 kcal/mol

Entry A TS minor3: The transition state structure leading to the minor product in the click reaction between ABSACN Entry A and BnN_3 with CH_2Cl_2 solvent.

Table S35. Calculated thermodynamic quantities for TS minor3 in the cycloaddition reaction of ABSACN Entry A with Benzyl azide under PCM/CH_2Cl_2 solvent conditions.

	E (kcal/mol)	H (kcal/mol)	G (kcal/mol)	CV (cal/(mol·K))	CP (cal/(mol·K))	S (cal/(mol·K))
ELEC.	0	0	0	0	0	0
TRANS.	0.889	1.481	-11.487	2.981	4.968	43.495
ROT.	0.889	0.889	-9.743	2.981	2.981	35.659
VIB.	216.399	216.399	192.066	78.462	78.462	81.612
TOTAL	218.176	218.769	170.836	84.423	86.411	160.766
VIB. THERMAL CORRECTION E(T)-E(0) = H(T)-H(0) = 11925.430 cal/mol						
Imaginary Frequency: -350 cm^{-1}						

Note: All thermodynamic properties were calculated at 298.15 K and 1.0 atm. All values are calculated at the B3LYP-D3(BJ)/6-31G(d) level using the PCM solvation model (solvent=CH_2Cl_2). The presence of a single imaginary frequency (-350 cm^{-1}) confirms the nature of the transition state. ELEC., TRANS., ROT., and VIB. denote electronic, translational, rotational, and vibrational contributions, respectively.

RESULTS OF PCM CALCULATION
FREE ENERGY IN SOLVENT = <PSI| H(0)+V/2 |PSI> = -1479.6147461819 a.u.

INTERNAL ENERGY IN SOLVENT = <PSI| H(0) |PSI> = -1479.5944736437 a.u.
DELTA INTERNAL ENERGY = <D-PSI| H(0) |D-PSI> = 0.0000000000 a.u.
ELECTROSTATIC INTERACTION = -0.0202725381 a.u.
PIEROTTI CAVITATION ENERGY = 0.0000000000 a.u.
DISPERSION FREE ENERGY = 0.0000000000 a.u.
REPULSION FREE ENERGY = 0.0000000000 a.u.
TOTAL INTERACTION (DELTA + ES + CAV + DISP + REP) = -0.0202725381 a.u.
TOTAL FREE ENERGY IN SOLVENT = -1479.6147461819 a.u.

FREE ENERGY IN SOLVENT = -928472.37 kcal/mol
INTERNAL ENERGY IN SOLVENT = -928459.65 kcal/mol
DELTA INTERNAL ENERGY = 0.00 kcal/mol
ELECTROSTATIC INTERACTION = -12.72 kcal/mol
PIEROTTI CAVITATION ENERGY = 0.00 kcal/mol
DISPERSION FREE ENERGY = 0.00 kcal/mol
REPULSION FREE ENERGY = 0.00 kcal/mol
TOTAL INTERACTION = -12.72 kcal/mol
TOTAL FREE ENERGY IN SOLVENT = -928472.37 kcal/mol

Entry A GS2 Major product: The ground state structure (GS2) of the major product obtained from the click reaction between ABSACN Entry A and BnN_3 with CH_2Cl_2 as the solvent.

Table S36. Calculated thermodynamic quantities for the Major Product (GS2 Major) in the cycloaddition reaction of ABSACN Entry A with Benzyl azide under PCM/CH_2Cl_2 solvent conditions.

	E (kcal/mol)	H (kcal/mol)	G (kcal/mol)	CV (cal/(mol·K))	CP (cal/(mol·K))	S (cal/(mol·K))
ELEC.	0	0	0	0	0	0
TRANS.	0.889	1.481	-11.487	2.981	4.968	43.495
ROT.	0.889	0.889	-9.578	2.981	2.981	35.106
VIB.	219.675	219.675	198.87	74.461	74.461	69.779
TOTAL	221.452	222.045	177.805	80.423	82.41	148.38
VIB. THERMAL CORRECTION E(T)-E(0) = H(T)-H(0) = 10750.711 cal/mol						

Note: All thermodynamic properties were calculated at 298.15 K and 1.0 atm. All values are calculated at the B3LYP-D3(BJ)/6-31G(d) level using the PCM solvation model (solvent=CH_2Cl_2). ELEC., TRANS., ROT., and VIB. denote electronic, translational, rotational, and vibrational contributions, respectively. The structure corresponds to a ground state (GS), confirmed by the absence of imaginary frequencies.

RESULTS OF PCM CALCULATION
FREE ENERGY IN SOLVENT = <PSI| H(0)+V/2 |PSI> = -1479.7690488618 a.u.
INTERNAL ENERGY IN SOLVENT = <PSI| H(0) |PSI> = -1479.7467680179 a.u.
DELTA INTERNAL ENERGY = <D-PSI| H(0) |D-PSI> = 0.0000000000 a.u.
ELECTROSTATIC INTERACTION = -0.0222808440 a.u.
PIEROTTI CAVITATION ENERGY = 0.0000000000 a.u.
DISPERSION FREE ENERGY = 0.0000000000 a.u.
REPULSION FREE ENERGY = 0.0000000000 a.u.
TOTAL INTERACTION (DELTA + ES + CAV + DISP + REP) = -0.0222808440 a.u.
TOTAL FREE ENERGY IN SOLVENT = -1479.7690488618 a.u.

FREE ENERGY IN SOLVENT = -928569.20 kcal/mol
INTERNAL ENERGY IN SOLVENT = -928555.22 kcal/mol
DELTA INTERNAL ENERGY = 0.00 kcal/mol
ELECTROSTATIC INTERACTION = -13.98 kcal/mol
PIEROTTI CAVITATION ENERGY = 0.00 kcal/mol
DISPERSION FREE ENERGY = 0.00 kcal/mol
REPULSION FREE ENERGY = 0.00 kcal/mol
TOTAL INTERACTION = -13.98 kcal/mol
TOTAL FREE ENERGY IN SOLVENT = -928569.20 kcal/mol

Entry A GS2 Minor product: The ground state structure (GS2) of the minor product obtained from the click reaction between ABSACN Entry A and BnN_3 with CH_2Cl_2 as the solvent.

Table S37. Calculated thermodynamic quantities for the Minor Product (GS2 Minor) in the cycloaddition reaction of

ABSACN Entry A with Benzyl azide under PCM/CH_2Cl_2 solvent conditions.

	E (kcal/mol)	H (kcal/mol)	G (kcal/mol)	CV (cal/(mol·K))	CP (cal/(mol·K))	S (cal/(mol·K))
ELEC.	0	0	0	0	0	0
TRANS.	0.889	1.481	-11.487	2.981	4.968	43.495
ROT.	0.889	0.889	-9.583	2.981	2.981	35.121
VIB.	219.824	219.824	199.347	74.405	74.405	68.681
TOTAL	221.602	222.194	178.277	80.366	82.354	147.298
VIB. THERMAL CORRECTION E(T)-E(0) = H(T)-H(0) = 10710.472 cal/mol						

Note: All thermodynamic properties were calculated at 298.15 K and 1.0 atm. All values are calculated at the B3LYP-D3(BJ)/6-31G(d) level using the PCM solvation model (solvent=CH_2Cl_2). ELEC., TRANS., ROT., and VIB. denote electronic, translational, rotational, and vibrational contributions, respectively. The structure corresponds to a ground state (GS), confirmed by the absence of imaginary frequencies.

RESULTS OF PCM CALCULATION
FREE ENERGY IN SOLVENT = <PSI| H(0)+V/2 |PSI> = -1479.7672917414 a.u.
INTERNAL ENERGY IN SOLVENT = <PSI| H(0) |PSI> = -1479.7449685617 a.u.
DELTA INTERNAL ENERGY = <D-PSI| H(0) |D-PSI> = 0.0000000000 a.u.
ELECTROSTATIC INTERACTION = -0.0223231797 a.u.
PIEROTTI CAVITATION ENERGY = 0.0000000000 a.u.
DISPERSION FREE ENERGY = 0.0000000000 a.u.
REPULSION FREE ENERGY = 0.0000000000 a.u.
TOTAL INTERACTION (DELTA + ES + CAV + DISP + REP) = -0.0223231797 a.u.
TOTAL FREE ENERGY IN SOLVENT = -1479.7672917414 a.u.

FREE ENERGY IN SOLVENT = -928568.09 kcal/mol
INTERNAL ENERGY IN SOLVENT = -928554.09 kcal/mol
DELTA INTERNAL ENERGY = 0.00 kcal/mol
ELECTROSTATIC INTERACTION = -14.01 kcal/mol
PIEROTTI CAVITATION ENERGY = 0.00 kcal/mol
DISPERSION FREE ENERGY = 0.00 kcal/mol
REPULSION FREE ENERGY = 0.00 kcal/mol
TOTAL INTERACTION = -14.01 kcal/mol
TOTAL FREE ENERGY IN SOLVENT = -928568.09 kcal/mol

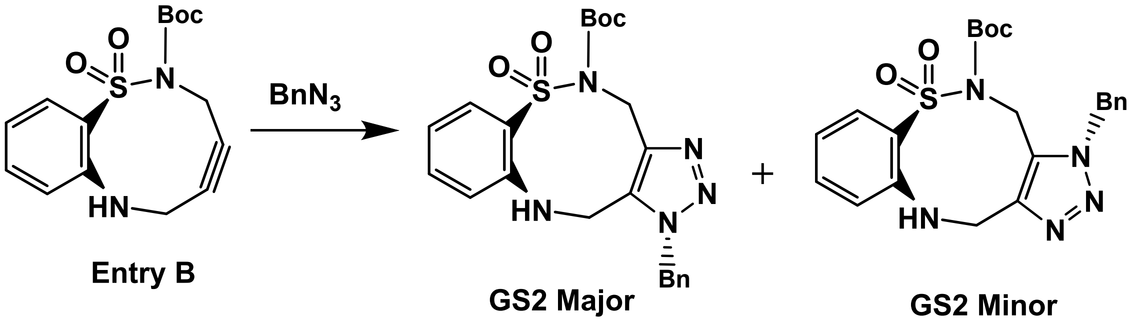

Figure S7. Formation of regioisomers in the strain-promoted cycloaddition of ABSACN Entry B (R^1=Boc, R^2=H) and Benzyl azide with PCM calculations applied.

Table S38. Calculated Free Energies for the Cycloaddition Reaction Pathway of ABSACN Entry B with Benzyl Azide in CH_2Cl_2 solvent.

Structure	Imaginary Frequency (cm^{-1})	Total Free Energy in Solvent (au)	Total Free Energy in Solvent (kcal/mol) (Relative to GS1)
GS1	N/A	-1825.28952	0
TS major1	-359	-1825.28295	4.12502

TS major2	-334	-1825.28107	5.30179
TS major3	-321	-1825.27988	6.04980
TS minor1	-358	-1825.28228	4.54472
TS minor2	-345	-1825.28048	5.67329
TS minor3	-357	-1825.28183	4.82896
GS2 Major	N/A	-1825.42984	-88.05321
GS2 Minor	N/A	-1825.43298	-90.02399

Note: All free energies were calculated at the B3LYP-D3(BJ)/6-31G(d) level using the PCM solvation model (solvent=CH_2Cl_2) at 298.15 K and 1.0 atm. Energies are reported as Total Free Energy in Solvent (au) and relative free energies (kcal/mol) with respect to the ground state of the reactants (GS1). The single imaginary frequency (cm^{-1}) confirms the nature of the transition states (TS). GS1 (Reactants) Total Free Energy in Solvent: -1825.28952 au.

GS1
Entry B solvent=CH_2Cl_2 + Benzyl azide solvent=CH_2Cl_2
The optimized ground state structure (GS1) representing the total energy of the reactants (each optimized ABSACN and optimized BnN_3) under dichloromethane (CH_2Cl_2) solvent conditions.

TS major1: The transition state structure leading to the major product in the click reaction between ABSACN Entry B and BnN_3 with CH_2Cl_2 solvent.

Table S39. Calculated thermodynamic quantities for TS major1 in the cycloaddition reaction of ABSACN Entry B with Benzyl azide under PCM/CH_2Cl_2 solvent conditions.

	E (kcal/mol)	H (kcal/mol)	G (kcal/mol)	CV (cal/(mol·K))	CP (cal/(mol·K))	S (cal/(mol·K))
ELEC.	0	0	0	0	0	0
TRANS.	0.889	1.481	-11.707	2.981	4.968	44.235
ROT.	0.889	0.889	-10.04	2.981	2.981	36.656
VIB.	301.746	301.746	268.26	109.116	109.116	112.311
TOTAL	303.523	304.116	246.513	115.077	117.064	193.202
VIB. THERMAL CORRECTION E(T)-E(0) = H(T)-H(0) = 16862.806 cal/mol						
Imaginary Frequency: -359 cm^{-1}						

Note: All thermodynamic properties were calculated at 298.15 K and 1.0 atm. All values are calculated at the B3LYP-D3(BJ)/6-31G(d) level using the PCM solvation model (solvent=CH_2Cl_2). The presence of a single imaginary frequency (-359 cm^{-1}) confirms the nature of the transition state. ELEC., TRANS., ROT., and VIB. denote electronic, translational, rotational, and vibrational contributions, respectively.

```
RESULTS OF PCM CALCULATION
 FREE ENERGY IN SOLVENT = <PSI| H(0)+V/2 |PSI>          =    -1825.2829473196 a.u.
 INTERNAL ENERGY IN SOLVENT = <PSI| H(0) |PSI>          =    -1825.2639510386 a.u.
 DELTA INTERNAL ENERGY =   <D-PSI| H(0) |D-PSI>         =        0.0000000000 a.u.
 ELECTROSTATIC INTERACTION                              =       -0.0189962810 a.u.
 PIEROTTI CAVITATION ENERGY                             =        0.0000000000 a.u.
 DISPERSION FREE ENERGY                                 =        0.0000000000 a.u.
 REPULSION FREE ENERGY                                  =        0.0000000000 a.u.
 TOTAL INTERACTION (DELTA + ES + CAV + DISP + REP)      =       -0.0189962810 a.u.
 TOTAL FREE ENERGY IN SOLVENT                           =    -1825.2829473196 a.u.

 FREE ENERGY IN SOLVENT         =    -1145382.46 kcal/mol
 INTERNAL ENERGY IN SOLVENT     =    -1145370.54 kcal/mol
 DELTA INTERNAL ENERGY          =           0.00 kcal/mol
 ELECTROSTATIC INTERACTION      =         -11.92 kcal/mol
 PIEROTTI CAVITATION ENERGY     =           0.00 kcal/mol
 DISPERSION FREE ENERGY         =           0.00 kcal/mol
```

REPULSION FREE ENERGY = 0.00 kcal/mol
TOTAL INTERACTION = -11.92 kcal/mol
TOTAL FREE ENERGY IN SOLVENT = -1145382.46 kcal/mol

TS major2: The transition state structure leading to the major product in the click reaction between ABSACN Entry B and BnN_3 with CH_2Cl_2 solvent.

Table S40. Calculated thermodynamic quantities for TS major2 in the cycloaddition reaction of ABSACN Entry B with Benzyl azide under PCM/CH_2Cl_2 solvent conditions.

	E (kcal/mol)	H (kcal/mol)	G (kcal/mol)	CV (cal/(mol·K))	CP (cal/(mol·K))	S (cal/(mol·K))
ELEC.	0	0	0	0	0	0
TRANS.	0.889	1.481	-11.707	2.981	4.968	44.235
ROT.	0.889	0.889	-10.039	2.981	2.981	36.653
VIB.	301.536	301.536	267.427	109.157	109.157	114.401
TOTAL	303.313	303.906	245.68	115.119	117.106	195.289
VIB. THERMAL CORRECTION E(T)-E(0) = H(T)-H(0) = 16917.941 cal/mol						
Imaginary Frequency: -334 cm⁻¹						

Note: All thermodynamic properties were calculated at 298.15 K and 1.0 atm. All values are calculated at the B3LYP-D3(BJ)/6-31G(d) level using the PCM solvation model (solvent=CH_2Cl_2). The presence of a single imaginary frequency (-334 cm^{-1}) confirms the nature of the transition state. ELEC., TRANS., ROT., and VIB. denote electronic, translational, rotational, and vibrational contributions, respectively.

RESULTS OF PCM CALCULATION
FREE ENERGY IN SOLVENT = <PSI| H(0)+V/2 |PSI> = -1825.2810720248 a.u.
INTERNAL ENERGY IN SOLVENT = <PSI| H(0) |PSI> = -1825.2630797845 a.u.
DELTA INTERNAL ENERGY = <D-PSI| H(0) |D-PSI> = 0.0000000000 a.u.
ELECTROSTATIC INTERACTION = -0.0179922403 a.u.
PIEROTTI CAVITATION ENERGY = 0.0000000000 a.u.
DISPERSION FREE ENERGY = 0.0000000000 a.u.
REPULSION FREE ENERGY = 0.0000000000 a.u.
TOTAL INTERACTION (DELTA + ES + CAV + DISP + REP) = -0.0179922403 a.u.
TOTAL FREE ENERGY IN SOLVENT = -1825.2810720248 a.u.

FREE ENERGY IN SOLVENT = -1145381.29 kcal/mol
INTERNAL ENERGY IN SOLVENT = -1145370.00 kcal/mol
DELTA INTERNAL ENERGY = 0.00 kcal/mol
ELECTROSTATIC INTERACTION = -11.29 kcal/mol
PIEROTTI CAVITATION ENERGY = 0.00 kcal/mol
DISPERSION FREE ENERGY = 0.00 kcal/mol
REPULSION FREE ENERGY = 0.00 kcal/mol
TOTAL INTERACTION = -11.29 kcal/mol
TOTAL FREE ENERGY IN SOLVENT = -1145381.29 kcal/mol

TS major3: The transition state structure leading to the major product in the click reaction between ABSACN Entry B and BnN_3 with CH_2Cl_2 solvent.

Table S41. Calculated thermodynamic quantities for TS major3 in the cycloaddition reaction of ABSACN Entry B with Benzyl azide under PCM/CH_2Cl_2 solvent conditions.

	E (kcal/mol)	H (kcal/mol)	G (kcal/mol)	CV (cal/(mol·K))	CP (cal/(mol·K))	S (cal/(mol·K))
ELEC.	0	0	0	0	0	0
TRANS.	0.889	1.481	-11.707	2.981	4.968	44.235
ROT.	0.889	0.889	-10.179	2.981	2.981	37.123
VIB.	301.73	301.73	267.913	108.99	108.99	113.42
TOTAL	303.507	304.1	246.027	114.952	116.939	194.778

VIB. THERMAL CORRECTION E(T)-E(0) = H(T)-H(0) = 16880.177 cal/mol
Imaginary Frequency: -321 cm^{-1}

Note: All thermodynamic properties were calculated at 298.15 K and 1.0 atm. All values are calculated at the B3LYP-D3(BJ)/6-31G(d) level using the PCM solvation model (solvent=CH_2Cl_2). The presence of a single imaginary frequency (-321 cm^{-1}) confirms the nature of the transition state. ELEC., TRANS., ROT., and VIB. denote electronic, translational, rotational, and vibrational contributions, respectively.

RESULTS OF PCM CALCULATION
FREE ENERGY IN SOLVENT = <PSI| H(0)+V/2 |PSI> = -1825.2798799846 a.u.
INTERNAL ENERGY IN SOLVENT = <PSI| H(0) |PSI> = -1825.2616892279 a.u.
DELTA INTERNAL ENERGY = <D-PSI| H(0) |D-PSI> = 0.0000000000 a.u.
ELECTROSTATIC INTERACTION = -0.0181907567 a.u.
PIEROTTI CAVITATION ENERGY = 0.0000000000 a.u.
DISPERSION FREE ENERGY = 0.0000000000 a.u.
REPULSION FREE ENERGY = 0.0000000000 a.u.
TOTAL INTERACTION (DELTA + ES + CAV + DISP + REP) = -0.0181907567 a.u.
TOTAL FREE ENERGY IN SOLVENT = -1825.2798799846 a.u.

FREE ENERGY IN SOLVENT = -1145380.54 kcal/mol
INTERNAL ENERGY IN SOLVENT = -1145369.12 kcal/mol
DELTA INTERNAL ENERGY = 0.00 kcal/mol
ELECTROSTATIC INTERACTION = -11.41 kcal/mol
PIEROTTI CAVITATION ENERGY = 0.00 kcal/mol
DISPERSION FREE ENERGY = 0.00 kcal/mol
REPULSION FREE ENERGY = 0.00 kcal/mol
TOTAL INTERACTION = -11.41 kcal/mol
TOTAL FREE ENERGY IN SOLVENT = -1145380.54 kcal/mol

TS minor1: The transition state structure leading to the minor product in the click reaction between ABSACN Entry B and BnN_3 with CH_2Cl_2 solvent.

Table S42. Calculated thermodynamic quantities for TS minor1 in the cycloaddition reaction of ABSACN Entry B with Benzyl azide under PCM/CH_2Cl_2 solvent conditions.

	E (kcal/mol)	H (kcal/mol)	G (kcal/mol)	CV (cal/(mol·K))	CP (cal/(mol·K))	S (cal/(mol·K))
ELEC.	0	0	0	0	0	0
TRANS.	0.889	1.481	-11.707	2.981	4.968	44.235
ROT.	0.889	0.889	-10.019	2.981	2.981	36.584
VIB.	301.842	301.842	268.04	108.963	108.963	113.371
TOTAL	303.619	304.212	246.314	114.925	116.912	194.19
VIB. THERMAL CORRECTION E(T)-E(0) = H(T)-H(0) = 16841.166 cal/mol						
Imaginary Frequency: -358 cm^{-1}						

Note: All thermodynamic properties were calculated at 298.15 K and 1.0 atm. All values are calculated at the B3LYP-D3(BJ)/6-31G(d) level using the PCM solvation model (solvent=CH_2Cl_2). The presence of a single imaginary frequency (-358 cm^{-1}) confirms the nature of the transition state. ELEC., TRANS., ROT., and VIB. denote electronic, translational, rotational, and vibrational contributions, respectively.

RESULTS OF PCM CALCULATION
FREE ENERGY IN SOLVENT = <PSI| H(0)+V/2 |PSI> = -1825.2822784929 a.u.
INTERNAL ENERGY IN SOLVENT = <PSI| H(0) |PSI> = -1825.2654604791 a.u.
DELTA INTERNAL ENERGY = <D-PSI| H(0) |D-PSI> = 0.0000000000 a.u.
ELECTROSTATIC INTERACTION = -0.0168180138 a.u.
PIEROTTI CAVITATION ENERGY = 0.0000000000 a.u.
DISPERSION FREE ENERGY = 0.0000000000 a.u.
REPULSION FREE ENERGY = 0.0000000000 a.u.
TOTAL INTERACTION (DELTA + ES + CAV + DISP + REP) = -0.0168180138 a.u.
TOTAL FREE ENERGY IN SOLVENT = -1825.2822784929 a.u.

FREE ENERGY IN SOLVENT = -1145382.04 kcal/mol
INTERNAL ENERGY IN SOLVENT = -1145371.49 kcal/mol
DELTA INTERNAL ENERGY = 0.00 kcal/mol
ELECTROSTATIC INTERACTION = -10.55 kcal/mol
PIEROTTI CAVITATION ENERGY = 0.00 kcal/mol
DISPERSION FREE ENERGY = 0.00 kcal/mol
REPULSION FREE ENERGY = 0.00 kcal/mol
TOTAL INTERACTION = -10.55 kcal/mol
TOTAL FREE ENERGY IN SOLVENT = -1145382.04 kcal/mol

TS minor2: The transition state structure leading to the minor product in the click reaction between ABSACN Entry B and BnN_3 with CH_2Cl_2 solvent.

Table S43. Calculated thermodynamic quantities for TS minor2 in the cycloaddition reaction of ABSACN Entry B with Benzyl azide under PCM/CH_2Cl_2 solvent conditions.

	E (kcal/mol)	H (kcal/mol)	G (kcal/mol)	CV (cal/(mol·K))	CP (cal/(mol·K))	S (cal/(mol·K))
ELEC.	0	0	0	0	0	0
TRANS.	0.889	1.481	-11.707	2.981	4.968	44.235
ROT.	0.889	0.889	-9.901	2.981	2.981	36.189
VIB.	301.54	301.54	267.491	109.405	109.405	114.202
TOTAL	303.318	303.91	245.883	115.367	117.354	194.625
VIB. THERMAL CORRECTION E(T)-E(0) = H(T)-H(0) = 16911.557 cal/mol						
Imaginary Frequency: -345 cm^{-1}						

Note: All thermodynamic properties were calculated at 298.15 K and 1.0 atm. All values are calculated at the B3LYP-D3(BJ)/6-31G(d) level using the PCM solvation model (solvent=CH_2Cl_2). The presence of a single imaginary frequency (-345 cm^{-1}) confirms the nature of the transition state. ELEC., TRANS., ROT., and VIB. denote electronic, translational, rotational, and vibrational contributions, respectively.

RESULTS OF PCM CALCULATION
FREE ENERGY IN SOLVENT = <PSI| H(0)+V/2 |PSI> = -1825.2832689007 a.u.
INTERNAL ENERGY IN SOLVENT = <PSI| H(0) |PSI> = -1825.2679526166 a.u.
DELTA INTERNAL ENERGY = <D-PSI| H(0) |D-PSI> = 0.0000000000 a.u.
ELECTROSTATIC INTERACTION = -0.0153162840 a.u.
PIEROTTI CAVITATION ENERGY = 0.0000000000 a.u.
DISPERSION FREE ENERGY = 0.0000000000 a.u.
REPULSION FREE ENERGY = 0.0000000000 a.u.
TOTAL INTERACTION (DELTA + ES + CAV + DISP + REP) = -0.0153162840 a.u.
TOTAL FREE ENERGY IN SOLVENT = -1825.2832689007 a.u.

FREE ENERGY IN SOLVENT = -1145382.67 kcal/mol
INTERNAL ENERGY IN SOLVENT = -1145373.06 kcal/mol
DELTA INTERNAL ENERGY = 0.00 kcal/mol
ELECTROSTATIC INTERACTION = -9.61 kcal/mol
PIEROTTI CAVITATION ENERGY = 0.00 kcal/mol
DISPERSION FREE ENERGY = 0.00 kcal/mol
REPULSION FREE ENERGY = 0.00 kcal/mol
TOTAL INTERACTION = -9.61 kcal/mol
TOTAL FREE ENERGY IN SOLVENT = -1145382.67 kcal/mol

TS minor3: The transition state structure leading to the minor product in the click reaction between ABSACN Entry B and BnN_3 with CH_2Cl_2 solvent.

Table S44. Calculated thermodynamic quantities for TS minor3 in the cycloaddition reaction of ABSACN Entry B with Benzyl azide under PCM/CH_2Cl_2 solvent conditions.

	E (kcal/mol)	H (kcal/mol)	G (kcal/mol)	CV (cal/(mol·K))	CP (cal/(mol·K))	S (cal/(mol·K))
ELEC.	0	0	0	0	0	0

TRANS.	0.889	1.481	-11.707	2.981	4.968	44.235
ROT.	0.889	0.889	-10.065	2.981	2.981	36.738
VIB.	301.796	301.796	268.523	108.951	108.951	111.597
TOTAL	303.574	304.166	246.751	114.912	116.899	192.571
VIB. THERMAL CORRECTION E(T)-E(0) = H(T)-H(0) = 16805.536 cal/mol						
Imaginary Frequency: -357 cm^{-1}						

Note: All thermodynamic properties were calculated at 298.15 K and 1.0 atm. All values are calculated at the B3LYP-D3(BJ)/6-31G(d) level using the PCM solvation model (solvent=CH_2Cl_2). The presence of a single imaginary frequency (-357 cm^{-1}) confirms the nature of the transition state. ELEC., TRANS., ROT., and VIB. denote electronic, translational, rotational, and vibrational contributions, respectively.

RESULTS OF PCM CALCULATION
FREE ENERGY IN SOLVENT = <PSI| H(0)+V/2 |PSI> = -1825.2818255226 a.u.
INTERNAL ENERGY IN SOLVENT = <PSI| H(0) |PSI> = -1825.2669166311 a.u.
DELTA INTERNAL ENERGY = <D-PSI| H(0) |D-PSI> = 0.0000000000 a.u.
ELECTROSTATIC INTERACTION = -0.0149088915 a.u.
PIEROTTI CAVITATION ENERGY = 0.0000000000 a.u.
DISPERSION FREE ENERGY = 0.0000000000 a.u.
REPULSION FREE ENERGY = 0.0000000000 a.u.
TOTAL INTERACTION (DELTA + ES + CAV + DISP + REP) = -0.0149088915 a.u.
TOTAL FREE ENERGY IN SOLVENT = -1825.2818255226 a.u.

FREE ENERGY IN SOLVENT = -1145381.76 kcal/mol
INTERNAL ENERGY IN SOLVENT = -1145372.41 kcal/mol
DELTA INTERNAL ENERGY = 0.00 kcal/mol
ELECTROSTATIC INTERACTION = -9.36 kcal/mol
PIEROTTI CAVITATION ENERGY = 0.00 kcal/mol
DISPERSION FREE ENERGY = 0.00 kcal/mol
REPULSION FREE ENERGY = 0.00 kcal/mol
TOTAL INTERACTION = -9.36 kcal/mol
TOTAL FREE ENERGY IN SOLVENT = -1145381.76 kcal/mol

GS2 Major: The ground state structure (GS2) of the major product obtained from the click reaction between ABSACN Entry B and BnN_3 with CH_2Cl_2 as the solvent.

Table S45. Calculated thermodynamic quantities for the Major Product (GS2 Major) in the cycloaddition reaction of ABSACN Entry B with Benzyl azide under PCM/CH_2Cl_2 solvent conditions.

	E (kcal/mol)	H (kcal/mol)	G (kcal/mol)	CV (cal/(mol·K))	CP (cal/(mol·K))	S (cal/(mol·K))
ELEC.	0	0	0	0	0	0
TRANS.	0.889	1.481	-11.707	2.981	4.968	44.235
ROT.	0.889	0.889	-10.003	2.981	2.981	36.53
VIB.	304.933	304.933	273.483	105.47	105.47	105.486
TOTAL	306.711	307.303	251.772	111.432	113.419	186.252
VIB. THERMAL CORRECTION E(T)-E(0) = H(T)-H(0) = 15900.608 cal/mol						

Note: All thermodynamic properties were calculated at 298.15 K and 1.0 atm. All values are calculated at the B3LYP-D3(BJ)/6-31G(d) level using the PCM solvation model (solvent=CH_2Cl_2). ELEC., TRANS., ROT., and VIB. denote electronic, translational, rotational, and vibrational contributions, respectively. The structure corresponds to a ground state (GS), confirmed by the absence of imaginary frequencies.

RESULTS OF PCM CALCULATION
FREE ENERGY IN SOLVENT = <PSI| H(0)+V/2 |PSI> = -1825.4298425536 a.u.
INTERNAL ENERGY IN SOLVENT = <PSI| H(0) |PSI> = -1825.4052892048 a.u.
DELTA INTERNAL ENERGY = <D-PSI| H(0) |D-PSI> = 0.0000000000 a.u.
ELECTROSTATIC INTERACTION = -0.0245533489 a.u.
PIEROTTI CAVITATION ENERGY = 0.0000000000 a.u.
DISPERSION FREE ENERGY = 0.0000000000 a.u.

REPULSION FREE ENERGY = 0.0000000000 a.u.
TOTAL INTERACTION (DELTA + ES + CAV + DISP + REP) = -0.0245533489 a.u.
TOTAL FREE ENERGY IN SOLVENT = -1825.4298425536 a.u.

FREE ENERGY IN SOLVENT = -1145474.64 kcal/mol
INTERNAL ENERGY IN SOLVENT = -1145459.24 kcal/mol
DELTA INTERNAL ENERGY = 0.00 kcal/mol
ELECTROSTATIC INTERACTION = -15.41 kcal/mol
PIEROTTI CAVITATION ENERGY = 0.00 kcal/mol
DISPERSION FREE ENERGY = 0.00 kcal/mol
REPULSION FREE ENERGY = 0.00 kcal/mol
TOTAL INTERACTION = -15.41 kcal/mol
TOTAL FREE ENERGY IN SOLVENT = -1145474.64 kcal/mol

GS2 Minor: The ground state structure (GS2) of the minor product obtained from the click reaction between ABSACN Entry B and BnN_3 with CH_2Cl_2 as the solvent.

Table S46. Calculated thermodynamic quantities for the Minor Product (GS2 Minor) in the cycloaddition reaction of ABSACN Entry B with Benzyl azide under PCM/CH_2Cl_2 solvent conditions.

	E (kcal/mol)	H (kcal/mol)	G (kcal/mol)	CV (cal/(mol·K))	CP (cal/(mol·K))	S (cal/(mol·K))
ELEC.	0	0	0	0	0	0
TRANS.	0.889	1.481	-11.707	2.981	4.968	44.235
ROT.	0.889	0.889	-9.953	2.981	2.981	36.363
VIB.	304.691	304.691	272.851	105.967	105.967	106.792
TOTAL	306.469	307.061	251.191	111.929	113.916	187.391
VIB. THERMAL CORRECTION E(T)-E(0) = H(T)-H(0) = 16028.762 cal/mol						

Note: All thermodynamic properties were calculated at 298.15 K and 1.0 atm. All values are calculated at the B3LYP-D3(BJ)/6-31G(d) level using the PCM solvation model (solvent=CH_2Cl_2). ELEC., TRANS., ROT., and VIB. denote electronic, translational, rotational, and vibrational contributions, respectively. The structure corresponds to a ground state (GS), confirmed by the absence of imaginary frequencies.

RESULTS OF PCM CALCULATION
FREE ENERGY IN SOLVENT = <PSI| H(0)+V/2 |PSI> = -1825.4329831971 a.u.
INTERNAL ENERGY IN SOLVENT = <PSI| H(0) |PSI> = -1825.4142996203 a.u.
DELTA INTERNAL ENERGY = <D-PSI| H(0) |D-PSI> = 0.0000000000 a.u.
ELECTROSTATIC INTERACTION = -0.0186835768 a.u.
PIEROTTI CAVITATION ENERGY = 0.0000000000 a.u.
DISPERSION FREE ENERGY = 0.0000000000 a.u.
REPULSION FREE ENERGY = 0.0000000000 a.u.
TOTAL INTERACTION (DELTA + ES + CAV + DISP + REP) = -0.0186835768 a.u.
TOTAL FREE ENERGY IN SOLVENT = -1825.4329831971 a.u.

FREE ENERGY IN SOLVENT = -1145476.61 kcal/mol
INTERNAL ENERGY IN SOLVENT = -1145464.89 kcal/mol
DELTA INTERNAL ENERGY = 0.00 kcal/mol
ELECTROSTATIC INTERACTION = -11.72 kcal/mol
PIEROTTI CAVITATION ENERGY = 0.00 kcal/mol
DISPERSION FREE ENERGY = 0.00 kcal/mol
REPULSION FREE ENERGY = 0.00 kcal/mol
TOTAL INTERACTION = -11.72 kcal/mol
TOTAL FREE ENERGY IN SOLVENT = -1145476.61 kcal/mol

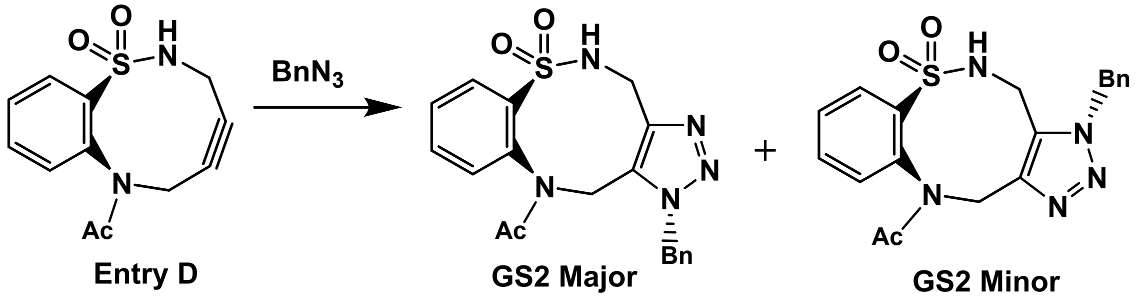

Figure S8. Formation of regioisomers in the strain-promoted cycloaddition of ABSACN Entry D (R^1=H, R^2=Ac) and Benzyl azide with PCM calculations applied.

Table S47. Calculated Free Energies for the Cycloaddition Reaction Pathway of ABSACN Entry D with Benzyl Azide in CH_2Cl_2 solvent.

Structure	Imaginary Frequency (cm^{-1})	Total Free Energy in Solvent (au)	Total Free Energy in Solvent (kcal/mol) (Relative to GS1)
GS1	N/A	-1632.21965	0
TS major1	-341	-1632.20874	6.84373
TS minor1	-339	-1632.20722	7.80196
GS2 Major	N/A	-1632.36004	-88.09790
GS2 Minor	N/A	-1632.35784	-86.71641

Note: All free energies were calculated at the B3LYP-D3(BJ)/6-31G(d) level using the PCM solvation model (solvent=CH_2Cl_2) at 298.15 K and 1.0 atm. Energies are reported as Total Free Energy in Solvent (au) and relative free energies (kcal/mol) with respect to the ground state of the reactants (GS1). The single imaginary frequency (cm^{-1}) confirms the nature of the transition states (TS). GS1 (Reactants) Total Free Energy in Solvent: -1632.21965 au.

GS1
Entry D solvent=CH_2Cl_2 + Benzyl azide solvent=CH_2Cl_2
The optimized ground state structure (GS1) representing the total energy of the reactants (each optimized ABSACN and optimized BnN_3) under dichloromethane (CH_2Cl_2) solvent conditions.

TS major1: The transition state structure leading to the major product in the click reaction between ABSACN Entry D and BnN_3 with CH_2Cl_2 solvent.

Table S48. Calculated thermodynamic quantities for TS major1 in the cycloaddition reaction of ABSACN Entry D with Benzyl azide under PCM/CH_2Cl_2 solvent conditions.

	E (kcal/mol)	H (kcal/mol)	G (kcal/mol)	CV (cal/(mol·K))	CP (cal/(mol·K))	S (cal/(mol·K))
ELEC.	0	0	0	0	0	0
TRANS.	0.889	1.481	-11.586	2.981	4.968	43.828
ROT.	0.889	0.889	-9.838	2.981	2.981	35.978
VIB.	242.035	242.035	214.223	90.372	90.372	93.281
TOTAL	243.812	244.405	192.799	96.333	98.32	173.088
VIB. THERMAL CORRECTION E(T)-E(0) = H(T)-H(0) = 13976.849 cal/mol						

Imaginary Frequency: -341 cm⁻¹

Note: All thermodynamic properties were calculated at 298.15 K and 1.0 atm. All values are calculated at the B3LYP-D3(BJ)/6-31G(d) level using the PCM solvation model (solvent=CH_2Cl_2). The presence of a single imaginary frequency (-341 cm^{-1}) confirms the nature of the transition state. ELEC., TRANS., ROT., and VIB. denote electronic, translational, rotational, and vibrational contributions, respectively.

RESULTS OF PCM CALCULATION
FREE ENERGY IN SOLVENT = <PSI| H(0)+V/2 |PSI> = -1632.2087448394 a.u.
INTERNAL ENERGY IN SOLVENT = <PSI| H(0) |PSI> = -1632.1823538408 a.u.
DELTA INTERNAL ENERGY = <D-PSI| H(0) |D-PSI> = 0.0000000000 a.u.
ELECTROSTATIC INTERACTION = -0.0263909986 a.u.
PIEROTTI CAVITATION ENERGY = 0.0000000000 a.u.
DISPERSION FREE ENERGY = 0.0000000000 a.u.
REPULSION FREE ENERGY = 0.0000000000 a.u.
TOTAL INTERACTION (DELTA + ES + CAV + DISP + REP) = -0.0263909986 a.u.
TOTAL FREE ENERGY IN SOLVENT = -1632.2087448394 a.u.

FREE ENERGY IN SOLVENT = -1024226.56 kcal/mol
INTERNAL ENERGY IN SOLVENT = -1024210.00 kcal/mol
DELTA INTERNAL ENERGY = 0.00 kcal/mol
ELECTROSTATIC INTERACTION = -16.56 kcal/mol
PIEROTTI CAVITATION ENERGY = 0.00 kcal/mol
DISPERSION FREE ENERGY = 0.00 kcal/mol
REPULSION FREE ENERGY = 0.00 kcal/mol
TOTAL INTERACTION = -16.56 kcal/mol
TOTAL FREE ENERGY IN SOLVENT = -1024226.56 kcal/mol

TS minor1: The transition state structure leading to the minor product in the click reaction between ABSACN Entry D and BnN_3 with CH_2Cl_2 solvent.

Table S49. Calculated thermodynamic quantities for TS minor1 in the cycloaddition reaction of ABSACN Entry D with Benzyl azide under PCM/CH_2Cl_2 solvent conditions.

	E (kcal/mol)	H (kcal/mol)	G (kcal/mol)	CV (cal/(mol·K))	CP (cal/(mol·K))	S (cal/(mol·K))
ELEC.	0	0	0	0	0	0
TRANS.	0.889	1.481	-11.586	2.981	4.968	43.828
ROT.	0.889	0.889	-9.883	2.981	2.981	36.128
VIB.	242.24	242.24	214.407	90.039	90.039	93.355
TOTAL	244.018	244.61	192.937	96.001	97.988	173.311
VIB. THERMAL CORRECTION E(T)-E(0) = H(T)-H(0) = 13893.656 cal/mol						
Imaginary Frequency: -339 cm⁻¹						

Note: All thermodynamic properties were calculated at 298.15 K and 1.0 atm. All values are calculated at the B3LYP-D3(BJ)/6-31G(d) level using the PCM solvation model (solvent= CH_2Cl_2). The presence of a single imaginary frequency (-339 cm^{-1}) confirms the nature of the transition state. ELEC., TRANS., ROT., and VIB. denote electronic, translational, rotational, and vibrational contributions, respectively.

RESULTS OF PCM CALCULATION
FREE ENERGY IN SOLVENT = <PSI| H(0)+V/2 |PSI> = -1632.2072177979 a.u.
INTERNAL ENERGY IN SOLVENT = <PSI| H(0) |PSI> = -1632.1797771080 a.u.
DELTA INTERNAL ENERGY = <D-PSI| H(0) |D-PSI> = 0.0000000000 a.u.
ELECTROSTATIC INTERACTION = -0.0274406899 a.u.
PIEROTTI CAVITATION ENERGY = 0.0000000000 a.u.
DISPERSION FREE ENERGY = 0.0000000000 a.u.
REPULSION FREE ENERGY = 0.0000000000 a.u.
TOTAL INTERACTION (DELTA + ES + CAV + DISP + REP) = -0.0274406899 a.u.
TOTAL FREE ENERGY IN SOLVENT = -1632.2072177979 a.u.

FREE ENERGY IN SOLVENT = -1024225.60 kcal/mol
INTERNAL ENERGY IN SOLVENT = -1024208.38 kcal/mol
DELTA INTERNAL ENERGY = 0.00 kcal/mol

ELECTROSTATIC INTERACTION = -17.22 kcal/mol
PIEROTTI CAVITATION ENERGY = 0.00 kcal/mol
DISPERSION FREE ENERGY = 0.00 kcal/mol
REPULSION FREE ENERGY = 0.00 kcal/mol
TOTAL INTERACTION = -17.22 kcal/mol
TOTAL FREE ENERGY IN SOLVENT = -1024225.60 kcal/mol

GS2 Major: The ground state structure (GS2) of the major product obtained from the click reaction between ABSACN Entry D and BnN_3 with CH_2Cl_2 as the solvent.

Table S50. Calculated thermodynamic quantities for the Major Product (GS2 Major) in the cycloaddition reaction of ABSACN Entry D with Benzyl azide under PCM/CH_2Cl_2 solvent conditions.

	E (kcal/mol)	H (kcal/mol)	G (kcal/mol)	CV (cal/(mol·K))	CP (cal/(mol·K))	S (cal/(mol·K))
ELEC.	0	0	0	0	0	0
TRANS.	0.889	1.481	-11.586	2.981	4.968	43.828
ROT.	0.889	0.889	-9.715	2.981	2.981	35.565
VIB.	245.116	245.116	219.794	86.519	86.519	84.932
TOTAL	246.894	247.486	198.492	92.481	94.468	164.326
VIB. THERMAL CORRECTION E(T)-E(0) = H(T)-H(0) = 12982.833 cal/mol						

Note: All thermodynamic properties were calculated at 298.15 K and 1.0 atm. All values are calculated at the B3LYP-D3(BJ)/6-31G(d) level using the PCM solvation model (solvent=CH_2Cl_2). ELEC., TRANS., ROT., and VIB. denote electronic, translational, rotational, and vibrational contributions, respectively. The structure corresponds to a ground state (GS), confirmed by the absence of imaginary frequencies.

RESULTS OF PCM CALCULATION
FREE ENERGY IN SOLVENT = <PSI| H(0)+V/2 |PSI> = -1632.3600438287 a.u.
INTERNAL ENERGY IN SOLVENT = <PSI| H(0) |PSI> = -1632.3330054969 a.u.
DELTA INTERNAL ENERGY = <D-PSI| H(0) |D-PSI> = 0.0000000000 a.u.
ELECTROSTATIC INTERACTION = -0.0270383319 a.u.
PIEROTTI CAVITATION ENERGY = 0.0000000000 a.u.
DISPERSION FREE ENERGY = 0.0000000000 a.u.
REPULSION FREE ENERGY = 0.0000000000 a.u.
TOTAL INTERACTION (DELTA + ES + CAV + DISP + REP) = -0.0270383319 a.u.
TOTAL FREE ENERGY IN SOLVENT = -1632.3600438287 a.u.

FREE ENERGY IN SOLVENT = -1024321.50 kcal/mol
INTERNAL ENERGY IN SOLVENT = -1024304.54 kcal/mol
DELTA INTERNAL ENERGY = 0.00 kcal/mol
ELECTROSTATIC INTERACTION = -16.97 kcal/mol
PIEROTTI CAVITATION ENERGY = 0.00 kcal/mol
DISPERSION FREE ENERGY = 0.00 kcal/mol
REPULSION FREE ENERGY = 0.00 kcal/mol
TOTAL INTERACTION = -16.97 kcal/mol
TOTAL FREE ENERGY IN SOLVENT = -1024321.50 kcal/mol

GS2 Minor: The ground state structure (GS2) of the minor product obtained from the click reaction between ABSACN Entry D and BnN_3 with CH_2Cl_2 as the solvent.

Table S51. Calculated thermodynamic quantities for the Minor Product (GS2 Minor) in the cycloaddition reaction of ABSACN Entry D with Benzyl azide under PCM/CH_2Cl_2 solvent conditions.

	E (kcal/mol)	H (kcal/mol)	G (kcal/mol)	CV (cal/(mol·K))	CP (cal/(mol·K))	S (cal/(mol·K))
ELEC.	0	0	0	0	0	0
TRANS.	0.889	1.481	-11.586	2.981	4.968	43.828
ROT.	0.889	0.889	-9.745	2.981	2.981	35.667
VIB.	245.147	245.147	219.453	86.532	86.532	86.177

TOTAL	246.924	247.517	198.121	92.494	94.481	165.673
VIB. THERMAL CORRECTION E(T)-E(0) = H(T)-H(0) = 12965.200 cal/mol						

Note: All thermodynamic properties were calculated at 298.15 K and 1.0 atm. All values are calculated at the B3LYP-D3(BJ)/6-31G(d) level using the PCM solvation model (solvent=CH_2Cl_2). ELEC., TRANS., ROT., and VIB. denote electronic, translational, rotational, and vibrational contributions, respectively. The structure corresponds to a ground state (GS), confirmed by the absence of imaginary frequencies.

RESULTS OF PCM CALCULATION
FREE ENERGY IN SOLVENT = <PSI| H(0)+V/2 |PSI> = -1632.3578422848 a.u.
INTERNAL ENERGY IN SOLVENT = <PSI| H(0) |PSI> = -1632.3308264346 a.u.
DELTA INTERNAL ENERGY = <D-PSI| H(0) |D-PSI> = 0.0000000000 a.u.
ELECTROSTATIC INTERACTION = -0.0270158502 a.u.
PIEROTTI CAVITATION ENERGY = 0.0000000000 a.u.
DISPERSION FREE ENERGY = 0.0000000000 a.u.
REPULSION FREE ENERGY = 0.0000000000 a.u.
TOTAL INTERACTION (DELTA + ES + CAV + DISP + REP) = -0.0270158502 a.u.
TOTAL FREE ENERGY IN SOLVENT = -1632.3578422848 a.u.

FREE ENERGY IN SOLVENT = -1024320.12 kcal/mol
INTERNAL ENERGY IN SOLVENT = -1024303.17 kcal/mol
DELTA INTERNAL ENERGY = 0.00 kcal/mol
ELECTROSTATIC INTERACTION = -16.95 kcal/mol
PIEROTTI CAVITATION ENERGY = 0.00 kcal/mol
DISPERSION FREE ENERGY = 0.00 kcal/mol
REPULSION FREE ENERGY = 0.00 kcal/mol
TOTAL INTERACTION = -16.95 kcal/mol
TOTAL FREE ENERGY IN SOLVENT = -1024320.12 kcal/mol

Table S52. Calculated Free Energies for the Cycloaddition Reaction Pathway of ABSACN Entry D with Benzyl Azide in CH_3CN solvent.

Structure	Imaginary Frequency (cm^{-1})	Total Free Energy in Solvent (au)	Total Free Energy in Solvent (kcal/mol) (Relative to GS1)
GS1	N/A	-1632.22227	0
TS major1	-364	-1632.21239	6.19486
TS minor1	-339	-1632.20983	7.80423
GS2 Major	N/A	-1632.36265	-88.09464
GS2 Minor	N/A	-1632.36042	-86.69445

Note: All free energies were calculated at the B3LYP-D3(BJ)/6-31G(d) level using the PCM solvation model (solvent= CH_3CN) at 298.15 K and 1.0 atm. Energies are reported as Total Free Energy in Solvent (au) and relative free energies (kcal/mol) with respect to the ground state of the reactants (GS1). The single imaginary frequency (cm^{-1}) confirms the nature of the transition states (TS). GS1 (Reactants) Total Free Energy in Solvent: -1632.22227 au.

GS1
Entry D solvent= CH_3CN + Benzyl azide□solvent= CH_3CN

Entry D□solvent=CH_3CN: The optimized structure of ABSACN Entry D under CH_3CN solvent conditions.
Table S53. Calculated thermodynamic quantities for the ABSACN Entry D (R^1=H, R^2=Ac) under PCM/ CH_3CN solvent conditions.

	E (kcal/mol)	H (kcal/mol)	G (kcal/mol)	CV (cal/(mol·K))	CP (cal/(mol·K))	S (cal/(mol·K))
ELEC.	0	0	0	0	0	0
TRANS.	0.889	1.481	-11.224	2.981	4.968	42.612
ROT.	0.889	0.889	-8.873	2.981	2.981	32.743
VIB.	152.534	152.534	137.986	55.952	55.952	48.793

TOTAL	154.312	154.904	117.889	61.913	63.901	124.148
VIB. THERMAL CORRECTION E(T)-E(0) = H(T)-H(0) =	8146.012 cal/mol					

Note: All thermodynamic properties were calculated at 298.15 K and 1.0 atm. All values are calculated at the B3LYP-D3(BJ)/6-31G(d) level using the PCM solvation model (solvent= CH_3CN). ELEC., TRANS., ROT., and VIB. denote electronic, translational, rotational, and vibrational contributions, respectively. The structure corresponds to a ground state (GS), confirmed by the absence of imaginary frequencies.

RESULTS OF PCM CALCULATION
FREE ENERGY IN SOLVENT = <PSI| H(0)+V/2 |PSI> = -1197.2987138534 a.u.
INTERNAL ENERGY IN SOLVENT = <PSI| H(0) |PSI> = -1197.2742025973 a.u.
DELTA INTERNAL ENERGY = <D-PSI| H(0) |D-PSI> = 0.0000000000 a.u.
ELECTROSTATIC INTERACTION = -0.0245112562 a.u.
PIEROTTI CAVITATION ENERGY = 0.0000000000 a.u.
DISPERSION FREE ENERGY = 0.0000000000 a.u.
REPULSION FREE ENERGY = 0.0000000000 a.u.
TOTAL INTERACTION (DELTA + ES + CAV + DISP + REP) = -0.0245112562 a.u.
TOTAL FREE ENERGY IN SOLVENT = -1197.2987138534 a.u.

FREE ENERGY IN SOLVENT = -751316.37 kcal/mol
INTERNAL ENERGY IN SOLVENT = -751300.99 kcal/mol
DELTA INTERNAL ENERGY = 0.00 kcal/mol
ELECTROSTATIC INTERACTION = -15.38 kcal/mol
PIEROTTI CAVITATION ENERGY = 0.00 kcal/mol
DISPERSION FREE ENERGY = 0.00 kcal/mol
REPULSION FREE ENERGY = 0.00 kcal/mol
TOTAL INTERACTION = -15.38 kcal/mol
TOTAL FREE ENERGY IN SOLVENT = -751316.37 kcal/mol

BnN_3□ solvent=CH_3CN: The optimized structure of Benzyl azide under CH_3CN solvent conditions.
Table S54. Calculated thermodynamic quantities for the Benzyl azide (BnN_3) reactant model (GS1) under PCM/CH_3CN solvent conditions.

	E (kcal/mol)	H (kcal/mol)	G (kcal/mol)	CV (cal/(mol·K))	CP (cal/(mol·K))	S (cal/(mol·K))
ELEC.	0	0	0	0	0	0
TRANS.	0.889	1.481	-10.615	2.981	4.968	40.569
ROT.	0.889	0.889	-7.896	2.981	2.981	29.464
VIB.	86.729	86.729	79.935	24.943	24.943	22.788
TOTAL	88.506	89.099	61.424	30.905	32.892	92.821
VIB. THERMAL CORRECTION E(T)-E(0) = H(T)-H(0) =	3502.124 cal/mol					

Note: All thermodynamic properties were calculated at 298.15 K and 1.0 atm. All values are calculated at the B3LYP-D3(BJ)/6-31G(d) level using the PCM solvation model (solvent= CH_3CN). ELEC., TRANS., ROT., and VIB. denote electronic, translational, rotational, and vibrational contributions, respectively. The structure corresponds to a ground state (GS), confirmed by the absence of imaginary frequencies.

RESULTS OF PCM CALCULATION
FREE ENERGY IN SOLVENT = <PSI| H(0)+V/2 |PSI> = -434.9235514934 a.u.
INTERNAL ENERGY IN SOLVENT = <PSI| H(0) |PSI> = -434.9168626027 a.u.
DELTA INTERNAL ENERGY = <D-PSI| H(0) |D-PSI> = 0.0000000000 a.u.
ELECTROSTATIC INTERACTION = -0.0066888907 a.u.
PIEROTTI CAVITATION ENERGY = 0.0000000000 a.u.
DISPERSION FREE ENERGY = 0.0000000000 a.u.
REPULSION FREE ENERGY = 0.0000000000 a.u.
TOTAL INTERACTION (DELTA + ES + CAV + DISP + REP) = -0.0066888907 a.u.
TOTAL FREE ENERGY IN SOLVENT = -434.9235514934 a.u.

FREE ENERGY IN SOLVENT = -272918.68 kcal/mol
INTERNAL ENERGY IN SOLVENT = -272914.48 kcal/mol
DELTA INTERNAL ENERGY = 0.00 kcal/mol
ELECTROSTATIC INTERACTION = -4.20 kcal/mol

PIEROTTI CAVITATION ENERGY = 0.00 kcal/mol
DISPERSION FREE ENERGY = 0.00 kcal/mol
REPULSION FREE ENERGY = 0.00 kcal/mol
TOTAL INTERACTION = -4.20 kcal/mol
TOTAL FREE ENERGY IN SOLVENT = -272918.68 kcal/mol

TS major1: The transition state structure leading to the major product in the click reaction between ABSACN Entry D and BnN_3 with CH_3CN solvent.

Table S55. Calculated thermodynamic quantities for TS major1 in the cycloaddition reaction of ABSACN Entry D with Benzyl azide under PCM/CH_3CN solvent conditions.

	E (kcal/mol)	H (kcal/mol)	G (kcal/mol)	CV (cal/(mol·K))	CP (cal/(mol·K))	S (cal/(mol·K))
ELEC.	0	0	0	0	0	0
TRANS.	0.889	1.481	-11.586	2.981	4.968	43.828
ROT.	0.889	0.889	-9.79	2.981	2.981	35.815
VIB.	242.376	242.376	215.466	90.016	90.016	90.257
TOTAL	244.154	244.746	194.09	95.978	97.965	169.901
VIB. THERMAL CORRECTION E(T)-E(0) = H(T)-H(0) = 13799.590 cal/mol						
Imaginary Frequency: -364 cm^{-1}						

Note: All thermodynamic properties were calculated at 298.15 K and 1.0 atm. All values are calculated at the B3LYP-D3(BJ)/6-31G(d) level using the PCM solvation model (solvent= CH_3CN). The presence of a single imaginary frequency (-364 cm^{-1}) confirms the nature of the transition state. ELEC., TRANS., ROT., and VIB. denote electronic, translational, rotational, and vibrational contributions, respectively.

RESULTS OF PCM CALCULATION
FREE ENERGY IN SOLVENT = <PSI| H(0)+V/2 |PSI> = -1632.2123932095 a.u.
INTERNAL ENERGY IN SOLVENT = <PSI| H(0) |PSI> = -1632.1828581917 a.u.
DELTA INTERNAL ENERGY = <D-PSI| H(0) |D-PSI> = 0.0000000000 a.u.
ELECTROSTATIC INTERACTION = -0.0295350178 a.u.
PIEROTTI CAVITATION ENERGY = 0.0000000000 a.u.
DISPERSION FREE ENERGY = 0.0000000000 a.u.
REPULSION FREE ENERGY = 0.0000000000 a.u.
TOTAL INTERACTION (DELTA + ES + CAV + DISP + REP) = -0.0295350178 a.u.
TOTAL FREE ENERGY IN SOLVENT = -1632.2123932095 a.u.

FREE ENERGY IN SOLVENT = -1024228.85 kcal/mol
INTERNAL ENERGY IN SOLVENT = -1024210.32 kcal/mol
DELTA INTERNAL ENERGY = 0.00 kcal/mol
ELECTROSTATIC INTERACTION = -18.53 kcal/mol
PIEROTTI CAVITATION ENERGY = 0.00 kcal/mol
DISPERSION FREE ENERGY = 0.00 kcal/mol
REPULSION FREE ENERGY = 0.00 kcal/mol
TOTAL INTERACTION = -18.53 kcal/mol
TOTAL FREE ENERGY IN SOLVENT = -1024228.85 kcal/mol

TS minor1: The transition state structure leading to the minor product in the click reaction between ABSACN Entry D and BnN_3 with CH_3CN solvent.

Table S56. Calculated thermodynamic quantities for TS minor1 in the cycloaddition reaction of ABSACN Entry D with Benzyl azide under PCM/CH_3CN solvent conditions.

	E (kcal/mol)	H (kcal/mol)	G (kcal/mol)	CV (cal/(mol·K))	CP (cal/(mol·K))	S (cal/(mol·K))
ELEC.	0	0	0	0	0	0
TRANS.	0.889	1.481	-11.586	2.981	4.968	43.828
ROT.	0.889	0.889	-9.885	2.981	2.981	36.134

VIB.	242.288	242.288	214.569	89.981	89.981	92.969
TOTAL	244.065	244.658	193.098	95.943	97.93	172.932
VIB. THERMAL CORRECTION E(T)-E(0) = H(T)-H(0) = 13871.165 cal/mol						
Imaginary Frequency: -339 cm^{-1}						

Note: All thermodynamic properties were calculated at 298.15 K and 1.0 atm. All values are calculated at the B3LYP-D3(BJ)/6-31G(d) level using the PCM solvation model (solvent= CH_3CN). The presence of a single imaginary frequency (-339 cm^{-1}) confirms the nature of the transition state. ELEC., TRANS., ROT., and VIB. denote electronic, translational, rotational, and vibrational contributions, respectively.

RESULTS OF PCM CALCULATION
FREE ENERGY IN SOLVENT = <PSI| H(0)+V/2 |PSI> = -1632.2098285285 a.u.
INTERNAL ENERGY IN SOLVENT = <PSI| H(0) |PSI> = -1632.1783482807 a.u.
DELTA INTERNAL ENERGY = <D-PSI| H(0) |D-PSI> = 0.0000000000 a.u.
ELECTROSTATIC INTERACTION = -0.0314802478 a.u.
PIEROTTI CAVITATION ENERGY = 0.0000000000 a.u.
DISPERSION FREE ENERGY = 0.0000000000 a.u.
REPULSION FREE ENERGY = 0.0000000000 a.u.
TOTAL INTERACTION (DELTA + ES + CAV + DISP + REP) = -0.0314802478 a.u.
TOTAL FREE ENERGY IN SOLVENT = -1632.2098285285 a.u.

FREE ENERGY IN SOLVENT = -1024227.24 kcal/mol
INTERNAL ENERGY IN SOLVENT = -1024207.49 kcal/mol
DELTA INTERNAL ENERGY = 0.00 kcal/mol
ELECTROSTATIC INTERACTION = -19.75 kcal/mol
PIEROTTI CAVITATION ENERGY = 0.00 kcal/mol
DISPERSION FREE ENERGY = 0.00 kcal/mol
REPULSION FREE ENERGY = 0.00 kcal/mol
TOTAL INTERACTION = -19.75 kcal/mol
TOTAL FREE ENERGY IN SOLVENT = -1024227.24 kcal/mol

GS2 Major: The ground state structure (GS2) of the major product obtained from the click reaction between ABSACN Entry D and BnN_3 with CH_3CN as the solvent.

Table S57. Calculated thermodynamic quantities for the Major Product (GS2 Major) in the cycloaddition reaction of ABSACN Entry D with Benzyl azide under PCM/CH_3CN solvent conditions.

	E (kcal/mol)	H (kcal/mol)	G (kcal/mol)	CV (cal/(mol·K))	CP (cal/(mol·K))	S (cal/(mol·K))
ELEC.	0	0	0	0	0	0
TRANS.	0.889	1.481	-11.586	2.981	4.968	43.828
ROT.	0.889	0.889	-9.765	2.981	2.981	35.733
VIB.	244.968	244.968	220.139	86.489	86.489	83.277
TOTAL	246.746	247.338	198.788	92.451	94.438	162.838
VIB. THERMAL CORRECTION E(T)-E(0) = H(T)-H(0) = 12853.083 cal/mol						

Note: All thermodynamic properties were calculated at 298.15 K and 1.0 atm. All values are calculated at the B3LYP-D3(BJ)/6-31G(d) level using the PCM solvation model (solvent= CH_3CN). ELEC., TRANS., ROT., and VIB. denote electronic, translational, rotational, and vibrational contributions, respectively. The structure corresponds to a ground state (GS), confirmed by the absence of imaginary frequencies.

RESULTS OF PCM CALCULATION
FREE ENERGY IN SOLVENT = <PSI| H(0)+V/2 |PSI> = -1632.2389625104 a.u.
INTERNAL ENERGY IN SOLVENT = <PSI| H(0) |PSI> = -1632.2082621908 a.u.
DELTA INTERNAL ENERGY = <D-PSI| H(0) |D-PSI> = 0.0000000000 a.u.
ELECTROSTATIC INTERACTION = -0.0307003196 a.u.
PIEROTTI CAVITATION ENERGY = 0.0000000000 a.u.
DISPERSION FREE ENERGY = 0.0000000000 a.u.
REPULSION FREE ENERGY = 0.0000000000 a.u.
TOTAL INTERACTION (DELTA + ES + CAV + DISP + REP) = -0.0307003196 a.u.
TOTAL FREE ENERGY IN SOLVENT = -1632.2389625104 a.u.

FREE ENERGY IN SOLVENT = -1024245.52 kcal/mol
INTERNAL ENERGY IN SOLVENT = -1024226.26 kcal/mol

DELTA INTERNAL ENERGY = 0.00 kcal/mol
ELECTROSTATIC INTERACTION = -19.26 kcal/mol
PIEROTTI CAVITATION ENERGY = 0.00 kcal/mol
DISPERSION FREE ENERGY = 0.00 kcal/mol
REPULSION FREE ENERGY = 0.00 kcal/mol
TOTAL INTERACTION = -19.26 kcal/mol
TOTAL FREE ENERGY IN SOLVENT = -1024245.52 kcal/mol

GS2 Minor: The ground state structure (GS2) of the minor product obtained from the click reaction between ABSACN Entry D and BnN_3 with CH_3CN as the solvent.

Table S58. Calculated thermodynamic quantities for the Minor Product (GS2 Minor) in the cycloaddition reaction of ABSACN Entry D with Benzyl azide under PCM/CH_3CN solvent conditions.

	E (kcal/mol)	H (kcal/mol)	G (kcal/mol)	CV (cal/(mol·K))	CP (cal/(mol·K))	S (cal/(mol·K))
ELEC.	0	0	0	0	0	0
TRANS.	0.889	1.481	-11.586	2.981	4.968	43.828
ROT.	0.889	0.889	-9.744	2.981	2.981	35.664
VIB.	245.233	245.233	219.824	86.413	86.413	85.220
TOTAL	247.01	247.603	198.494	92.374	94.361	164.713
VIB. THERMAL CORRECTION E(T)-E(0) = H(T)-H(0) = 12931.039 cal/mol						

Note: All thermodynamic properties were calculated at 298.15 K and 1.0 atm. All values are calculated at the B3LYP-D3(BJ)/6-31G(d) level using the PCM solvation model (solvent= CH_3CN). ELEC., TRANS., ROT., and VIB. denote electronic, translational, rotational, and vibrational contributions, respectively. The structure corresponds to a ground state (GS), confirmed by the absence of imaginary frequencies.

RESULTS OF PCM CALCULATION
FREE ENERGY IN SOLVENT = <PSI| H(0)+V/2 |PSI> = -1632.3604216385 a.u.
INTERNAL ENERGY IN SOLVENT = <PSI| H(0) |PSI> = -1632.3297882423 a.u.
DELTA INTERNAL ENERGY = <D-PSI| H(0) |D-PSI> = 0.0000000000 a.u.
ELECTROSTATIC INTERACTION = -0.0306333963 a.u.
PIEROTTI CAVITATION ENERGY = 0.0000000000 a.u.
DISPERSION FREE ENERGY = 0.0000000000 a.u.
REPULSION FREE ENERGY = 0.0000000000 a.u.
TOTAL INTERACTION (DELTA + ES + CAV + DISP + REP) = -0.0306333963 a.u.
TOTAL FREE ENERGY IN SOLVENT = -1632.3604216385 a.u.

FREE ENERGY IN SOLVENT = -1024321.74 kcal/mol
INTERNAL ENERGY IN SOLVENT = -1024302.52 kcal/mol
DELTA INTERNAL ENERGY = 0.00 kcal/mol
ELECTROSTATIC INTERACTION = -19.22 kcal/mol
PIEROTTI CAVITATION ENERGY = 0.00 kcal/mol
DISPERSION FREE ENERGY = 0.00 kcal/mol
REPULSION FREE ENERGY = 0.00 kcal/mol
TOTAL INTERACTION = -19.22 kcal/mol
TOTAL FREE ENERGY IN SOLVENT = -1024321.74 kcal/mol

Boltzmann Distribution for Product Ratio
The ratio of products can be predicted using the Boltzmann distribution (or Eyring equation at constant temperature), which relates the relative populations to the energy difference:

$$\frac{Major\ Product}{Minor\ Product} = e^{-\frac{\Delta\Delta G\ddagger}{RT}}$$

R is the ideal gas constant (1.987 cal/(mol·K) or 0.001987 kcal/(mol·K))
T is the temperature in Kelvin. Since no temperature is specified, we'll assume room temperature (298.15 K), which is standard for these types of predictions unless otherwise stated.

Table S59. Comparison of Boltzmann Distribution Predictions and Observed Product Ratios for the Cycloaddition Reactions of ABSACN Derivatives (Entry A and B).

Entry	Transition State Energy Difference (ΔΔG‡, kcal/mol)	Product Ratio (Major : Minor)	Observed product ratio (Major : Minor)
Entry A	0.52	71:29	5 : 2(= 71.4:28.6)
Entry B	0.41	67:33	5 : 3(= 62.5:37.5)

Note: The predicted product ratio was calculated using the Boltzmann distribution: Major/Minor = $e^{-(\Delta\Delta G\ddagger)/RT}$.
$\Delta\Delta G^\ddagger$ is the difference in Gibbs free energy between the major and minor transition states (TS) in kcal/mol. The predicted ratio calculation assumes a temperature (T) of 298.15 K and the ideal gas constant (R) of 0.001987 kcal/(mol·K). The $\Delta\Delta G^\ddagger$ values were obtained from B3LYP-D3(BJ)/6-31G(d) PCM/CH_2Cl_2 calculations (relative to GS1). $\Delta\Delta G^\ddagger$ is the free energy difference between TS minor1 and TS major1 ($\Delta G^\ddagger_{minor1}$ - $\Delta G^\ddagger_{major1}$).

3.Cartesian Coordinates of Optimized Structures

ABSACN model: The optimized ground state structure (GS1) representing the total energy of the reactants (model molecules: optimized ABSACN Entry A and optimized MeN_3). (Table 1 & Table S2)

C	8.032096	5.011177	7.411510
C	8.717739	5.998100	7.547344
O	6.623756	9.586610	5.970628
N	5.983673	5.081813	6.195087
C	5.651087	7.403630	7.101762
C	5.331854	6.030424	7.019998
C	4.273956	5.566767	7.821239
H	4.008258	4.516607	7.743073
C	4.909488	8.267717	7.919352
C	9.307206	7.329687	7.479805
O	7.441772	7.328940	5.118990
S	7.008640	8.194659	6.228219
C	6.910852	4.188620	6.929865
H	6.369892	3.699916	7.745871
H	7.251657	3.404591	6.245381
H	6.518491	5.566217	5.475026
N	8.228087	8.343526	7.411421
C	3.869470	7.780881	8.698872
H	5.162741	9.321820	7.932009
C	3.560482	6.417705	8.653962
H	3.300128	8.457148	9.328916
H	2.743624	6.023787	9.252424
H	8.582308	9.296210	7.331772
H	9.976515	7.386403	6.609215
H	9.894296	7.572547	8.373413

MeN_3 model: The optimized ground state structure (GS1) representing the total energy of the reactants (model molecules: optimized ABSACN Entry A and optimized MeN_3). (Table 1 & Table S3)

N	0.560202	-3.042146	3.563729
N	0.320273	-3.338847	1.221031
N	0.472297	-3.250982	2.443277
C	0.739840	-4.618377	0.620991
H	0.171374	-5.464009	1.028462
H	0.537642	-4.538358	-0.447469
H	1.811649	-4.803144	0.767421

ABSACN Entry A solvent=CH_2Cl_2: The optimized structure of ABSACN Entry A under CH_2Cl_2 solvent conditions. (Table 2 & Table S22)

C	8.021798	5.013852	7.418190
C	8.702182	6.004057	7.563127
O	6.612059	9.575197	5.944341
N	5.980274	5.070543	6.191319
C	5.655254	7.397056	7.096016
C	5.329840	6.024673	7.013338
C	4.270584	5.565796	7.815898
H	4.002750	4.515778	7.744877
C	4.920154	8.264213	7.918549
C	9.299904	7.333006	7.496124
O	7.443108	7.329245	5.104749
S	7.012926	8.187158	6.225821
C	6.908818	4.180172	6.935669
H	6.367259	3.690889	7.750177
H	7.258007	3.397904	6.254545
H	6.521605	5.551877	5.474822
N	8.224471	8.352869	7.399235
C	3.876905	7.781840	8.696749
H	5.178465	9.316961	7.940096
C	3.560678	6.419770	8.649605
H	3.313348	8.460390	9.329328
H	2.742909	6.028910	9.248107
H	8.589045	9.302836	7.322889
H	9.982390	7.385890	6.637636
H	9.871655	7.585395	8.395566

ABSACN Entry B solvent=CH_2Cl_2: The optimized structure of ABSACN Entry B under CH_2Cl_2 solvent conditions. (Table 2 & Table S23)

S 6.603153 7.750081 5.239264
O 6.854118 6.428885 4.644504
O 5.953886 8.784618 4.435494
N 6.298254 5.157650 7.212815
C 5.695775 7.557543 6.771865
C 5.742121 6.397065 7.586207
C 5.111508 6.476415 8.841301
H 5.126858 5.592200 9.471625
C 4.950181 8.681221 7.158106
C 9.312054 7.355338 5.592062
C 9.134065 6.339261 6.626534
C 8.622947 5.558075 7.398491
C 7.523584 4.743779 7.941699
H 7.377961 4.886645 9.015513
H 7.689770 3.675708 7.770145
H 6.506424 5.151068 6.216521
N 8.193466 8.333087 5.646167
C 4.320034 8.717736 8.393331
H 4.888299 9.518467 6.477813
C 4.432167 7.616110 9.249785
H 3.745388 9.590308 8.687240
H 3.950412 7.631918 10.222967
C 8.432195 9.500145 6.395265
H 9.314059 6.892527 4.603181
H 10.223640 7.941438 5.701446
O 7.356763 10.279387 6.452294
O 9.523748 9.721526 6.888193
C 7.374782 11.533006 7.260406
C 7.581365 11.173154 8.730584
C 8.443062 12.483352 6.718828
C 5.981850 12.113005 7.025765
H 6.830551 10.442662 9.049649
H 8.577369 10.761436 8.902712
H 7.466778 12.075201 9.341186
H 9.447893 12.099049 6.893289
H 8.300917 12.641782 5.644414
H 8.343768 13.450689 7.222562
H 5.783220 12.220735 5.955020
H 5.207771 11.481266 7.468221
H 5.918154 13.101087 7.491715

ABSACN Entry C solvent=CH_2Cl_2: The optimized structure of ABSACN Entry C under CH_2Cl_2 solvent conditions. (Table 2 & Table S24)

S 6.886781 7.976958 5.370642
O 7.221078 6.772699 4.610121
O 6.303557 9.124748 4.675785
N 6.267062 5.085676 6.926455
C 5.784352 7.566138 6.745871
C 5.619733 6.290697 7.336681
C 4.734802 6.177430 8.415765
H 4.606597 5.194440 8.858509
C 5.014254 8.646457 7.192466
C 9.532563 7.545960 5.982336
C 9.190632 6.372629 6.778756
C 8.558657 5.506547 7.335593
C 7.438008 4.642552 7.706801
H 7.203158 4.706336 8.774310
H 7.618486 3.597322 7.449367
C 5.898750 4.359419 5.809191
N 8.396864 8.502625 6.033802
C 4.136710 8.504714 8.262738
H 5.108090 9.594586 6.685083
C 4.008500 7.266718 8.889886
H 3.551096 9.356333 8.594528
H 3.326449 7.139495 9.724695
C 8.591844 9.692711 6.756883
H 9.718493 7.267544 4.942506
H 10.397997 8.096963 6.350955

O 7.452355 10.347422 6.945340
O 9.699289 10.023484 7.141562
C 7.441890 11.694081 7.582543
C 7.945908 11.585345 9.021320
C 8.261244 12.661703 6.730714
C 5.961538 12.067019 7.554698
H 7.383679 10.816055 9.561399
H 9.008408 11.341870 9.057085
H 7.785310 12.543595 9.526556
H 9.319218 12.396256 6.732720
H 7.893787 12.664702 5.699177
H 8.154568 13.673399 7.135906
H 5.572817 12.036530 6.531975
H 5.376201 11.387234 8.180970
H 5.835033 13.081580 7.944273
O 6.521549 3.355662 5.475055
C 4.684705 4.847426 5.046652
H 4.292309 4.010599 4.466399
H 3.904224 5.243040 5.701002
H 4.988570 5.640267 4.356308

ABSACN Entry D solvent=CH_2Cl_2: The optimized structure of ABSACN Entry D under CH_2Cl_2 solvent conditions. (Table 2 & Table S25)

S 7.153461 8.379644 6.418987
O 7.713281 7.630027 5.288091
O 6.670967 9.752933 6.197027
N 6.208782 5.206264 6.146131
C 5.771381 7.473431 7.154109
C 5.438796 6.122558 6.926294
C 4.302557 5.603291 7.559577
H 4.053508 4.562114 7.377483
C 4.965040 8.262185 7.984819
C 9.386460 7.586366 7.800511
C 8.854898 6.230852 7.708747
C 8.198591 5.254846 7.434624
C 7.171905 4.368793 6.885461
H 6.645557 3.813837 7.669881
H 7.582969 3.656013 6.167749
C 6.158761 5.123826 4.769645
N 8.256997 8.543197 7.693319
C 3.841857 7.724215 8.604996
H 5.221972 9.304223 8.135177
C 3.507542 6.386903 8.390564
H 3.229435 8.350092 9.246238
H 2.630838 5.954852 8.863116
H 8.560371 9.517285 7.701769
H 10.131076 7.747340 7.009997
H 9.868520 7.787014 8.762877
O 6.873831 4.337958 4.152848
C 5.169496 6.032771 4.072135
H 4.989266 5.634007 3.072642
H 4.223525 6.118309 4.612684
H 5.603641 7.033202 3.979263

ABSACN Entry E solvent=CH_2Cl_2: The optimized structure of ABSACN Entry E under CH_2Cl_2 solvent conditions. (Table 2 & Table S26)

S 6.552976 7.431366 5.422248
O 7.029491 6.194631 4.786286
O 5.924105 8.458031 4.593560
N 6.168972 4.637538 7.092725
C 5.447263 7.031419 6.775702
C 5.442094 5.790192 7.462908
C 4.585971 5.682495 8.573618
H 4.557436 4.731818 9.098211
C 4.565318 8.062410 7.138281
C 9.200744 7.279613 6.216515
C 8.920272 6.150979 7.098136
C 8.377547 5.252828 7.702491

C 7.305606 4.282452 7.980523
H 6.974084 4.302450 9.022101
H 7.620266 3.259197 7.753715
H 6.529375 4.740655 6.146474
N 7.991110 8.136424 6.100853
C 3.718391 7.912665 8.226813
H 4.559455 8.973885 6.556139
C 3.750677 6.721751 8.960699
H 3.035391 8.711954 8.496164
H 3.092196 6.590130 9.814501
C 8.039287 9.396153 6.705326
H 9.481517 6.927128 5.220926
H 9.990170 7.937760 6.581183
O 6.837450 9.972780 6.758918
O 9.072605 9.877150 7.135348
C 6.790237 11.336322 7.288389
C 7.047819 12.351963 6.209491
H 5.775760 11.413408 7.681527
H 7.509401 11.411595 8.104271
C 6.012032 12.733254 5.347194
C 8.318754 12.916448 6.044653
C 8.548596 13.851711 5.033797
C 6.240996 13.663617 4.333707
C 7.511132 14.225007 4.176444
H 9.125334 12.616408 6.706888
H 9.536111 14.289735 4.916203
H 5.022274 12.301263 5.473909
H 5.430095 13.954448 3.671583
H 7.689813 14.953499 3.390031

ABSACN Entry F solvent=CH_2Cl_2: The optimized structure of ABSACN Entry F under CH_2Cl_2 solvent conditions. (Table 2 & Table S27)

S 6.624632 8.037714 5.987020
O 7.274664 7.019222 5.152520
O 5.818050 9.080611 5.352700
N 6.555936 4.925492 7.035533
C 5.623478 7.259734 7.252209
C 5.714612 5.898606 7.627734
C 4.888202 5.472397 8.681967
H 4.933259 4.424787 8.964564
C 4.717653 8.128998 7.880082
C 9.214930 8.190349 6.975280
C 9.020163 6.860168 7.535171
C 8.626240 5.761657 7.854525
C 7.744231 4.584080 7.860582
H 7.417829 4.310792 8.867777
H 8.234285 3.708016 7.425309
H 6.891605 5.257822 6.133450
N 7.887490 8.862011 6.829214
C 3.917122 7.673851 8.918510
H 4.668635 9.161428 7.557846
C 4.013545 6.338625 9.324350
H 3.221246 8.351601 9.402349
H 3.386705 5.967540 10.130061
S 7.770286 10.547689 7.185098
H 9.709762 8.128494 6.000097
H 9.808662 8.833289 7.626691
O 6.410014 10.800354 7.657754
O 8.915158 10.796476 8.067785
C 8.049563 11.405743 5.657235
C 6.965245 11.868580 4.913166
C 9.367764 11.606935 5.237483
C 9.593151 12.279030 4.041248
C 7.215730 12.543578 3.720712
C 8.524369 12.752682 3.263666
H 10.198413 11.264438 5.844988
H 10.614176 12.446087 3.709164
H 5.954426 11.701364 5.262648
H 6.378869 12.914584 3.135570
C 8.786026 13.456527 1.956565

H 9.636262 14.141840 2.037961
H 9.029159 12.729649 1.170658
H 7.911919 14.024294 1.625029

Model Major TS: The transition state structure leading to the major product in the click reaction between the model molecules, ABSACN Entry A and MeN_3. (Figure 2 & Table S4)

S -1.791233 -1.389487 0.346033
O -1.200476 -1.615766 1.674400
O -3.098025 -1.945283 -0.000887
N -0.167329 1.092285 1.653761
C -1.897807 0.376443 0.001895
C -1.072117 1.349639 0.604824
C -1.189861 2.672495 0.142146
H -0.582629 3.434749 0.621312
C -2.828933 0.733351 -0.984651
C 0.720215 -2.221003 -0.430890
C 1.471321 -0.979029 -0.151391
C 1.644257 0.104661 0.412817
C 1.255021 1.224896 1.289103
H 1.416555 2.190676 0.802548
H 1.851844 1.222529 2.208544
H -0.331714 0.165648 2.044348
N -0.700697 -2.041119 -0.765685
C -2.919660 2.046928 -1.426336
H -3.478738 -0.034393 -1.390037
C -2.083007 3.015966 -0.863218
H -3.641759 2.315628 -2.190937
H -2.149171 4.049938 -1.190465
H -0.875918 -1.680974 -1.700110
H 0.765229 -2.864198 0.452694
H 1.151967 -2.783124 -1.263089
N 3.356894 -1.292554 -1.297918
N 3.697166 0.760749 -0.343110
N 3.893606 -0.315118 -0.959817
C 4.834440 1.467272 0.242907
H 5.527432 1.826714 -0.527134
H 4.421739 2.334369 0.762881
H 5.384387 0.850698 0.965346

Model Minor TS: The transition state structure leading to the minor product in the click reaction between the model molecules, ABSACN Entry A and MeN_3. (Figure 2 & Table S5)

S 1.339768 1.621492 0.372286
O 0.711244 1.612154 1.702597
O 2.388754 2.586606 0.048607
N 0.613282 -1.278219 1.595580
C 2.001510 -0.002224 -0.033103
C 1.519333 -1.207100 0.523964
C 2.017008 -2.408860 -0.012981
H 1.674951 -3.341683 0.425257
C 2.979638 -0.010924 -1.039091
C -1.316989 1.646480 -0.367960
C -1.648801 0.249574 -0.031511
C -1.492532 -0.870272 0.466018
C -0.711688 -1.833991 1.272342
H -0.594366 -2.787964 0.750903
H -1.238107 -2.049127 2.208272
H 0.496959 -0.362121 2.024695
N 0.086132 1.897658 -0.734934
C 3.453664 -1.212667 -1.548254
H 3.357408 0.936564 -1.407853
C 2.953273 -2.415371 -1.037304
H 4.210569 -1.213756 -2.326327
H 3.318986 -3.363782 -1.421240
H 0.344600 1.552003 -1.656017
H -1.546025 2.285370 0.490344
H -1.905081 2.015783 -1.212443
N -3.771223 0.064153 -0.873208
N -3.374441 -1.954308 0.135777
N -3.944439 -1.080299 -0.385342

C -4.888424 1.008009 -0.858547
H -4.486369 1.966611 -1.193816
H -5.679554 0.707838 -1.555981
H -5.316402 1.135036 0.143622

GS2 model major: The ground state structure (GS2) of the major product obtained from the click reaction between the model molecules, ABSACN Entry A and MeN_3. (Table 3 & Table S6)

S -0.075068 -0.115533 0.067389
O 1.315523 0.155998 -0.334291
O -1.048862 0.957222 0.253329
N 1.383999 -2.416604 -1.526980
C -0.774135 -1.267332 -1.123030
C -0.000673 -2.289935 -1.714656
C -0.670270 -3.220815 -2.526539
H -0.087088 -3.997108 -3.013678
C -2.145867 -1.159596 -1.375567
C 1.077037 -1.883058 1.835763
C 0.727503 -3.286551 1.446757
C 1.064991 -4.023810 0.323834
C 1.852341 -3.659527 -0.906434
H 1.811783 -4.480633 -1.630431
H 2.911118 -3.518367 -0.664541
H 1.788001 -1.584899 -1.106848
N 0.034153 -0.881273 1.552436
C -2.784391 -2.091263 -2.186997
H -2.693878 -0.341547 -0.921152
C -2.039100 -3.130237 -2.751240
H -3.849317 -2.007122 -2.378911
H -2.524286 -3.862741 -3.390646
H -0.884712 -1.112111 1.923845
H 1.992033 -1.535761 1.352205
H 1.245759 -1.847046 2.916382
N -0.106510 -4.035942 2.225673
N 0.393025 -5.195095 0.495118
N -0.310440 -5.190649 1.654631
C 0.315301 -6.349330 -0.380830
H -0.332581 -7.074984 0.111391
H -0.120006 -6.065308 -1.343617
H 1.304906 -6.788436 -0.537340

GS2 model minor: The ground state structure (GS2) of the minor product obtained from the click reaction between the model molecules, ABSACN Entry A and MeN_3. (Table 3 & Table S7)

S -0.051240 -0.103901 -0.065623
O 1.363592 0.056144 -0.440408
O -0.935896 1.046117 0.104131
N 1.193918 -2.593967 -1.497690
C -0.841141 -1.234580 -1.203430
C -0.172055 -2.384726 -1.681337
C -0.944451 -3.336674 -2.371050
H -0.459968 -4.236340 -2.735834
C -2.196072 -1.009600 -1.476764
C 0.976480 -1.880736 1.782654
C 0.608439 -3.301730 1.475152
C 0.892700 -4.140923 0.409153
C 1.632186 -3.841748 -0.865737
H 1.496788 -4.693413 -1.539563
H 2.710276 -3.746422 -0.695379
H 1.676805 -1.768894 -1.159839
N -0.017969 -0.852113 1.446483
C -2.927070 -1.951453 -2.189396
H -2.655784 -0.097890 -1.111754
C -2.295152 -3.125662 -2.614405
H -3.976882 -1.778929 -2.404183
H -2.859232 -3.878797 -3.157602
H -0.959732 -1.004288 1.796958
H 1.892753 -1.606206 1.257336
H 1.191239 -1.773448 2.852520
N -0.152869 -4.077200 2.300097
N 0.297542 -5.346640 0.634583
N -0.331341 -5.312823 1.779584

C -0.746002 -3.747535 3.582407
H 0.024927 -3.461652 4.304476
H -1.468178 -2.930823 3.481047
H -1.260530 -4.642371 3.933318

GS1 Entry A: The optimized ground state structure (GS1) representing the total energy of the reactants (optimized ABSACN Entry A and optimized BnN_3). (Figure 3&4, Table S9))

C 8.032096 5.011177 7.411510
C 8.717739 5.998100 7.547344
O 6.623756 9.586610 5.970628
N 5.983673 5.081813 6.195087
C 5.651087 7.403630 7.101762
C 5.331854 6.030424 7.019998
C 4.273956 5.566767 7.821239
H 4.008258 4.516607 7.743073
C 4.909488 8.267717 7.919352
C 9.307206 7.329687 7.479805
O 7.441772 7.328940 5.118990
S 7.008640 8.194659 6.228219
C 6.910852 4.188620 6.929865
H 6.369892 3.699916 7.745871
H 7.251657 3.404591 6.245381
H 6.518491 5.566217 5.475026
N 8.228087 8.343526 7.411421
C 3.869470 7.780881 8.698872
H 5.162741 9.321820 7.932009
C 3.560482 6.417705 8.653962
H 3.300128 8.457148 9.328916
H 2.743624 6.023787 9.252424
H 8.582308 9.296210 7.331772
H 9.976515 7.386403 6.609215
H 9.894296 7.572547 8.373413

GS1 BnN_3: The optimized ground state structure (GS1) representing the total energy of the reactants (optimized ABSACN Entry A and optimized BnN_3). (Figure 3&4, Table S10))

N 0.777908 -2.955269 3.757762
N 0.446852 -3.431246 1.454845
N 0.643369 -3.241350 2.660057
C 0.931114 -4.748539 0.944790
H 0.306402 -5.551159 1.358018
H 0.746731 -4.701178 -0.130492
C 2.388483 -5.002511 1.243746
C 2.761751 -5.878351 2.267696
C 3.384346 -4.318913 0.534178
C 4.729199 -4.515125 0.839056
C 4.109182 -6.078375 2.574132
C 5.094385 -5.397114 1.859912
H 3.097842 -3.627794 -0.254710
H 5.494087 -3.982560 0.280473
H 1.993499 -6.408520 2.825993
H 4.386978 -6.763789 3.370040
H 6.143580 -5.551354 2.096503

GS2 major1: The ground state structure (GS2) of the major product (Type 1) obtained from the click reaction between ABSACN Entry A and BnN_3. (Figure 3, Table S17))

S -0.110866 -0.083870 0.074439
O 1.260906 0.218299 -0.367902
O -1.094095 0.968994 0.317830
N 1.326724 -2.334843 -1.603375
C -0.832853 -1.226174 -1.109992
C -0.065088 -2.231982 -1.737775
C -0.750990 -3.170027 -2.527841
H -0.175262 -3.937488 -3.037319
C -2.214579 -1.137904 -1.310459
C 1.128102 -1.863791 1.773089
C 0.796944 -3.268788 1.372932
C 1.109783 -3.978772 0.225816
C 1.843797 -3.579586 -1.025717
H 1.797990 -4.389267 -1.759788
H 2.907638 -3.424676 -0.817786

H 1.731427 -1.502445 -1.185464
N 0.060175 -0.876381 1.539973
C -2.868299 -2.073571 -2.104614
H -2.757395 -0.332208 -0.828525
C -2.128336 -3.098603 -2.701131
H -3.940715 -2.004737 -2.256934
H -2.626050 -3.836117 -3.324861
H -0.842362 -1.128201 1.936700
H 2.021764 -1.491697 1.268416
H 1.329558 -1.843263 2.848607
N 0.024342 -4.057144 2.177460
N 0.478240 -5.170839 0.405218
N -0.164376 -5.211373 1.598873
C 0.498765 -6.355309 -0.443230
H -0.215192 -7.043430 0.018848
H 0.114082 -6.089058 -1.433406
C 1.881886 -6.963127 -0.542492
C 2.652359 -7.139022 0.612998
C 2.400835 -7.353040 -1.779591
C 3.673977 -7.920672 -1.864353
C 3.924412 -7.700996 0.528399
C 4.438116 -8.093686 -0.710594
H 1.808718 -7.212555 -2.681385
H 4.069072 -8.218871 -2.831484
H 2.251058 -6.830099 1.574695
H 4.516080 -7.832123 1.430045
H 5.430723 -8.530327 -0.775263

GS2 major2: The ground state structure (GS2) of the major product (Type 2) obtained from the click reaction between ABSACN Entry A and BnN_3. (Figure 3, Table S18))

S -0.124276 -0.188275 -0.226529
O 1.221279 -0.036177 -0.808599
O -1.006097 0.959317 -0.026716
N 0.972466 -2.758485 -1.625827
C -1.032877 -1.391503 -1.195827
C -0.412241 -2.561078 -1.689037
C -1.237337 -3.527228 -2.288226
H -0.794088 -4.439241 -2.672590
C -2.405506 -1.173912 -1.352328
C 1.221587 -1.783596 1.576783
C 0.886760 -3.242189 1.470695
C 1.026390 -4.144747 0.426621
C 1.482449 -3.972052 -1.000990
H 1.182840 -4.845367 -1.588704
H 2.576467 -3.925456 -1.051305
H 1.476348 -1.916755 -1.365218
N 0.128472 -0.841572 1.292745
C -3.197220 -2.135613 -1.969855
H -2.830612 -0.250130 -0.976237
C -2.604874 -3.318135 -2.421259
H -4.262431 -1.967236 -2.093762
H -3.208746 -4.085842 -2.894138
H -0.742415 -1.019320 1.786055
H 2.053648 -1.504296 0.928182
H 1.536050 -1.589954 2.607054
N 0.380512 -3.906361 2.552417
N 0.601264 -5.321712 0.960619
N 0.205968 -5.162132 2.249025
C 0.471384 -6.633465 0.335702
H 1.394616 -6.863339 -0.204303
H 0.389132 -7.335362 1.170534
C -0.730177 -6.726858 -0.580274
C -1.991108 -6.332957 -0.118224
C -0.596935 -7.218787 -1.881163
C -1.713775 -7.324492 -2.714013
C -3.105083 -6.441217 -0.946449
C -2.969770 -6.938242 -2.245903
H 0.381787 -7.524140 -2.245362
H -1.600292 -7.707751 -3.724226
H -2.090698 -5.938210 0.889257

H -4.080022 -6.131911 -0.580631
H -3.840812 -7.022071 -2.890006

GS2 minor1: The ground state structure (GS2) of the minor product (Type 1) obtained from the click reaction between ABSACN Entry A and BnN_3. (Figure 4, Table S19))

S -0.158934 -0.045508 -0.132252
O 1.227010 0.185729 -0.571339
O -1.105901 1.053796 0.039893
N 1.190338 -2.487651 -1.567482
C -0.918412 -1.256418 -1.208370
C -0.193574 -2.368146 -1.696231
C -0.928812 -3.383435 -2.334484
H -0.399597 -4.255616 -2.703809
C -2.296596 -1.130726 -1.423238
C 1.030482 -1.719255 1.713423
C 0.715827 -3.158808 1.431106
C 1.030595 -4.004135 0.378903
C 1.725783 -3.688444 -0.916420
H 1.635131 -4.563297 -1.567795
H 2.797778 -3.515737 -0.770494
H 1.631481 -1.626840 -1.263140
N -0.020518 -0.740428 1.398144
C -2.992165 -2.134192 -2.085538
H -2.801469 -0.245164 -1.053384
C -2.300269 -3.270444 -2.519737
H -4.060032 -2.038167 -2.254490
H -2.834278 -4.070977 -3.024249
H -0.937465 -0.924882 1.795862
H 1.916746 -1.412809 1.155552
H 1.270089 -1.594872 2.774472
N -0.009019 -3.951113 2.272493
N 0.495949 -5.233146 0.631677
N -0.125806 -5.206427 1.781099
C -0.559104 -3.642688 3.586899
C 0.515831 -3.416796 4.629076
H -1.214280 -2.767889 3.508028
H -1.186352 -4.503293 3.836384
C 1.571353 -4.328609 4.750636
C 0.460882 -2.309511 5.479442
C 1.445625 -2.116685 6.450760
C 2.555669 -4.133972 5.717024
C 2.494179 -3.028497 6.570051
H -0.353758 -1.595315 5.381703
H 1.395870 -1.252239 7.106614
H 1.615601 -5.184549 4.082564
H 3.371961 -4.845227 5.805703
H 3.263350 -2.877752 7.322134

GS2 minor2: The ground state structure (GS2) of the minor product (Type 2) obtained from the click reaction between ABSACN Entry A and BnN_3. (Figure 4, Table S20))

S -0.772169 -0.374085 0.501510
O 0.494664 0.257835 0.095673
O -1.950874 0.414105 0.854392
N 1.058639 -2.110271 -1.370700
C -1.265260 -1.539080 -0.767203
C -0.317298 -2.330665 -1.454486
C -0.810170 -3.389668 -2.238776
H -0.100710 -4.027397 -2.755310
C -2.640505 -1.741831 -0.932193
C 0.807891 -2.053866 2.015764
C 0.798863 -3.441777 1.436189
C 1.302273 -3.952822 0.250396
C 1.903796 -3.224395 -0.916551
H 2.063462 -3.952235 -1.718748
H 2.883914 -2.798586 -0.675960
H 1.281938 -1.227042 -0.924221
N -0.413566 -1.250445 1.885836
C -3.101404 -2.773454 -1.740718
H -3.327737 -1.090325 -0.404884
C -2.174493 -3.610545 -2.372914

H -4.167700 -2.929021 -1.874008
H -2.520316 -4.432966 -2.993012
H -1.252543 -1.657006 2.290117
H 1.614223 -1.475598 1.560092
H 1.029806 -2.101676 3.088161
N 0.257824 -4.535937 2.049294
N 1.029501 -5.288049 0.196925
N 0.398955 -5.643548 1.283684
C -0.498518 -4.627527 3.290627
C -1.786936 -3.834160 3.227242
H -0.681736 -5.696924 3.428451
H 0.124197 -4.281981 4.122810
C -2.606045 -3.902296 2.092029
C -2.139709 -2.982996 4.279078
C -3.293442 -2.200089 4.199600
C -3.757777 -3.121286 2.014424
C -4.101194 -2.264126 3.063803
H -1.497326 -2.915520 5.154062
H -3.547176 -1.528217 5.014181
H -2.322900 -4.539352 1.258662
H -4.376014 -3.168616 1.123771
H -4.986714 -1.640048 2.990818

TS major1: The transition state structure (Type 1) leading to the major product in the click reaction between ABSACN Entry A and BnN_3. (Figure 3, Table S11)

S 2.757814 -1.411999 -0.673720
O 1.744605 -1.592374 -1.724772
O 4.085842 -2.011333 -0.795558
N 0.880991 1.137367 -1.356224
C 3.029377 0.342424 -0.364782
C 2.083783 1.349674 -0.655269
C 2.385217 2.657646 -0.234860
H 1.679054 3.445877 -0.478508
C 4.238860 0.652257 0.274634
C 0.630080 -2.169081 0.907609
C -0.117212 -0.894484 0.899137
C -0.445392 0.196469 0.423619
C -0.341228 1.301367 -0.548603
H -0.321789 2.274967 -0.050915
H -1.206634 1.296102 -1.220187
H 0.879227 0.211489 -1.781386
N 2.087640 -2.059142 0.733906
C 4.506934 1.951755 0.684377
H 4.960604 -0.140737 0.437648
C 3.563715 2.954283 0.435426
H 5.443803 2.184180 1.180954
H 3.762525 3.977711 0.741574
H 2.590141 -1.733275 1.555405
H 0.255051 -2.798558 0.095324
H 0.486144 -2.723381 1.838719
N -1.546464 -1.170496 2.602547
N -2.114038 0.905546 1.799911
N -2.133982 -0.180438 2.431761
C -3.407581 1.467866 1.354690
H -4.095304 1.507577 2.207770
H -3.189545 2.497076 1.060513
C -4.010102 0.703066 0.196552
C -4.539098 -0.579045 0.391470
C -4.015759 1.250606 -1.090130
C -4.536315 0.531086 -2.167650
C -5.056451 -1.300770 -0.682335
C -5.055276 -0.747465 -1.965202
H -3.613353 2.248852 -1.247529
H -4.535763 0.969065 -3.161904
H -4.542658 -1.015382 1.387106
H -5.463306 -2.294799 -0.519069
H -5.458845 -1.311043 -2.801768

TS major2: The transition state structure (Type 2) leading to the major product in the click reaction between

ABSACN Entry A and BnN$_3$. (Figure 3, Table S12)

S	3.382793	0.472138	-0.038757
O	3.200052	1.236388	1.204218
O	4.721284	0.168726	-0.543240
N	0.972267	-0.470698	1.909561
C	2.511325	-1.101050	0.051453
C	1.402449	-1.343385	0.891376
C	0.718786	-2.560703	0.720330
H	-0.124050	-2.769232	1.372898
C	2.947613	-2.068614	-0.865294
C	1.567461	2.346814	-0.938905
C	0.375952	1.833010	-0.234915
C	-0.219720	1.163380	0.612384
C	-0.338307	0.165756	1.690728
H	-1.084041	-0.598405	1.450697
H	-0.654580	0.644250	2.623606
H	1.670633	0.252089	2.075891
N	2.612096	1.358281	-1.255052
C	2.257784	-3.265024	-1.008238
H	3.834236	-1.863233	-1.455915
C	1.126485	-3.498521	-0.219229
H	2.601858	-4.009789	-1.719172
H	0.577925	-4.431536	-0.316559
H	2.403269	0.764301	-2.052988
H	2.028614	3.121754	-0.319430
H	1.305873	2.814492	-1.892155
N	-1.215302	3.173427	-1.059573
N	-2.296678	2.107575	0.652947
N	-2.075421	2.817164	-0.357132
C	-3.649460	1.590549	0.909067
H	-3.740872	1.503069	1.995187
H	-4.389961	2.328980	0.579428
C	-3.903291	0.244627	0.262270
C	-4.177282	-0.877404	1.049897
C	-3.873039	0.106370	-1.131370
C	-4.125876	-1.128807	-1.723434
C	-4.432898	-2.116963	0.458716
C	-4.409372	-2.243624	-0.929613
H	-3.651422	0.969829	-1.753597
H	-4.106769	-1.222427	-2.805883
H	-4.201001	-0.777975	2.133025
H	-4.652167	-2.978992	1.083068
H	-4.612767	-3.204963	-1.392953

TS major3: The transition state structure (Type 3) leading to the major product in the click reaction between ABSACN Entry A and BnN$_3$. (Figure 3, Table S13)

S	9.559718	6.967166	7.012666
O	9.970348	5.672542	7.576628
O	10.551703	7.921414	6.520412
N	7.734506	4.400385	6.295408
C	8.380246	6.725706	5.672025
C	7.575602	5.573706	5.533569
C	6.580289	5.597802	4.539957
H	5.973136	4.707382	4.404583
C	8.226732	7.821416	4.809599
C	8.113320	7.010939	9.360383
C	7.111651	5.985051	9.006212
C	6.638793	5.058756	8.339921
C	6.633242	4.093502	7.223301
H	5.684315	4.107441	6.677987
H	6.770793	3.075256	7.602851
H	8.607651	4.432960	6.818715
N	8.709520	7.757770	8.239582
C	7.234822	7.818237	3.837121
H	8.898093	8.666929	4.915824
C	6.400179	6.701942	3.717416
H	7.119713	8.669681	3.173193
H	5.623264	6.681636	2.957859
H	8.119747	8.490822	7.855356
H	8.929780	6.523662	9.901778

H 7.696229 7.769769 10.028181
N 5.819076 6.040669 10.812995
N 5.002736 4.244606 9.656214
N 5.120151 5.158207 10.513022
C 3.660714 3.709759 9.372885
H 3.822545 2.985152 8.568834
C 3.018869 3.044108 10.571090
H 3.006872 4.500037 8.979455
C 3.596783 1.904263 11.143226
C 1.856603 3.576764 11.134139
C 1.273061 2.977801 12.252916
C 3.018089 1.306748 12.260445
C 1.853309 1.842355 12.817626
H 1.408632 4.465571 10.696437
H 0.369521 3.400898 12.682795
H 4.510387 1.498126 10.716464
H 3.473640 0.422999 12.697754
H 1.402526 1.375792 13.689218

TS minor1:The transition state structure (Type 1) leading to the minor product in the click reaction between ABSACN Entry A and BnN_3. (Figure 4, Table S14)

S -2.100344 1.724054 -0.144225
O -1.109456 1.697682 -1.231602
O -2.954549 2.892458 0.058259
N -1.709195 -1.098899 -1.661272
C -3.196091 0.299347 -0.233423
C -2.846902 -0.918629 -0.856936
C -3.727665 -2.003012 -0.689509
H -3.483324 -2.937471 -1.185736
C -4.404352 0.433168 0.467750
C 0.145524 0.973870 1.264211
C 0.284627 -0.385770 0.711272
C 0.052255 -1.359970 -0.013908
C -0.679893 -2.005880 -1.124810
H -1.145515 -2.942582 -0.804893
H 0.016568 -2.259627 -1.930542
H -1.280847 -0.201181 -1.879244
N -1.219944 1.520412 1.291594
C -5.256231 -0.653733 0.612491
H -4.658091 1.399048 0.891090
C -4.901092 -1.880946 0.041493
H -6.190231 -0.545927 1.155163
H -5.559699 -2.739154 0.142735
H -1.811979 1.125198 2.017718
H 0.763880 1.662595 0.679997
H 0.498713 1.024141 2.298322
N 2.211536 -1.007193 1.715157
N 1.709852 -2.760349 0.328926
N 2.312794 -2.073174 1.051373
C 3.409045 -0.177371 1.887228
H 3.033926 0.733312 2.369186
H 4.087817 -0.648583 2.610030
C 4.155391 0.164103 0.611594
C 3.484965 0.331731 -0.606253
C 5.541503 0.346553 0.657018
C 6.249353 0.701916 -0.491168
C 4.193979 0.681384 -1.755681
C 5.575962 0.870038 -1.701935
H 6.070761 0.206481 1.597259
H 7.326237 0.838239 -0.441054
H 2.412000 0.176476 -0.659226
H 3.663314 0.806337 -2.695647
H 6.125331 1.140859 -2.599404

TS minor2 :The transition state structure (Type 2) leading to the minor product in the click reaction between ABSACN Entry A and BnN_3. (Figure 4, Table S15)

S 0.227100 2.054480 -1.176631
O 1.695855 1.984961 -1.189507
O -0.464582 3.341460 -1.139397

N 1.544980 -0.260297 -3.010563
C -0.464593 1.148408 -2.570168
C 0.202602 0.093188 -3.228836
C -0.524149 -0.622060 -4.199276
H -0.012352 -1.416802 -4.732800
C -1.777882 1.501279 -2.916150
C 0.626500 0.254494 0.875076
C 1.014552 -0.914977 0.063409
C 1.388796 -1.522429 -0.945896
C 1.760415 -1.571565 -2.376170
H 1.186863 -2.336218 -2.908006
H 2.816434 -1.842018 -2.479028
H 2.019584 0.457632 -2.466540
N -0.264089 1.219361 0.212470
C -2.469675 0.778324 -3.878850
H -2.237536 2.349840 -2.422188
C -1.837035 -0.298320 -4.509941
H -3.484672 1.057451 -4.144035
H -2.363090 -0.867671 -5.271085
H -1.231367 0.917560 0.128576
H 1.530476 0.792558 1.173808
H 0.106279 -0.029643 1.794319
N 1.407139 -2.455914 1.687390
N 1.967745 -3.524628 -0.260171
N 1.720466 -3.356588 0.865690
C 0.558245 -2.819731 2.829637
H 1.149756 -3.368986 3.573138
H 0.300888 -1.858357 3.287694
C -0.688898 -3.596660 2.464882
C -0.982310 -4.800070 3.111673
C -1.558259 -3.125660 1.472511
C -2.708045 -3.841440 1.146052
C -2.134080 -5.519238 2.785601
C -3.000687 -5.039043 1.804121
H -1.318198 -2.209057 0.940186
H -3.377107 -3.466303 0.376293
H -0.303030 -5.178692 3.872521
H -2.347319 -6.455691 3.293668
H -3.896694 -5.597208 1.547400

TS minor3 :The transition state structure (Type 2) leading to the minor product in the click reaction between ABSACN Entry A and BnN_3. (Figure 4, Table S16)

S -0.173852 -0.166817 0.268915
O 1.289687 -0.026518 0.217026
O -1.027156 1.005088 0.456759
N 1.360470 -2.127739 -1.786555
C -0.789969 -1.023142 -1.187952
C -0.023055 -1.942646 -1.938636
C -0.694932 -2.677466 -2.933309
H -0.113133 -3.368752 -3.536177
C -2.146610 -0.808706 -1.473465
C 0.558737 -2.048900 2.146572
C 1.145322 -3.052859 1.240382
C 1.527768 -3.544659 0.173031
C 1.791248 -3.442117 -1.278368
H 1.281888 -4.234608 -1.834342
H 2.862439 -3.558947 -1.472486
H 1.757611 -1.395202 -1.200669
N -0.501864 -1.210057 1.566564
C -2.780847 -1.544000 -2.465780
H -2.686545 -0.059829 -0.905199
C -2.047822 -2.495213 -3.184122
H -3.830398 -1.373522 -2.683767
H -2.529532 -3.075067 -3.966488
H -1.381046 -1.698027 1.415281
H 1.345335 -1.377199 2.502743
H 0.108005 -2.514461 3.027495
N 1.848531 -4.589242 2.745806
N 2.562056 -5.412762 0.729207
N 2.445524 -5.282397 1.880893
C 2.552606 -4.313022 4.011435

H 3.520346 -3.834067 3.815435
H 1.916949 -3.578680 4.516546
C 2.735792 -5.539318 4.879203
C 4.017022 -5.967176 5.235314
C 1.623944 -6.262246 5.328600
C 1.793610 -7.391456 6.126363
C 4.189176 -7.096938 6.037534
C 3.077817 -7.810337 6.484602
H 0.626337 -5.940728 5.039491
H 0.924554 -7.945510 6.470570
H 4.884531 -5.413427 4.884080
H 5.190778 -7.418606 6.308810
H 3.209188 -8.690132 7.108648

TS Entry A major1 solvent=CH_2Cl_2: The transition state structure leading to the major product in the click reaction between ABSACN Entry A and BnN_3 with CH_2Cl_2 solvent. (Table 4, Table S30)

S 2.787510 -1.405342 -0.652081
O 1.789154 -1.615801 -1.715795
O 4.124748 -1.996075 -0.790137
N 0.885328 1.113783 -1.371469
C 3.040066 0.348724 -0.360771
C 2.082681 1.341871 -0.662588
C 2.364443 2.655344 -0.245621
H 1.648852 3.433595 -0.493132
C 4.239799 0.675279 0.291455
C 0.677229 -2.187240 0.935509
C -0.080299 -0.919108 0.905183
C -0.428503 0.160125 0.413828
C -0.341653 1.264007 -0.562825
H -0.334564 2.238860 -0.068636
H -1.206022 1.244765 -1.234539
H 0.891634 0.184878 -1.788354
N 2.136902 -2.074148 0.745629
C 4.488313 1.980270 0.695088
H 4.969314 -0.105181 0.476914
C 3.534771 2.970188 0.432022
H 5.416538 2.224448 1.201430
H 3.717324 3.996905 0.736021
H 2.641103 -1.752396 1.569549
H 0.294199 -2.843961 0.148831
H 0.555594 -2.716864 1.883101
N -1.528867 -1.211807 2.586587
N -2.093798 0.854646 1.768663
N -2.120137 -0.223410 2.407989
C -3.377295 1.456442 1.340020
H -4.049828 1.507089 2.203125
H -3.134246 2.480192 1.049179
C -4.010475 0.710746 0.186022
C -4.567487 -0.560151 0.381998
C -4.020720 1.267049 -1.097455
C -4.577630 0.567694 -2.171123
C -5.121736 -1.261391 -0.688066
C -5.125081 -0.700034 -1.968545
H -3.598369 2.256793 -1.255254
H -4.581356 1.012698 -3.162230
H -4.571796 -1.000897 1.375787
H -5.552501 -2.245200 -0.523469
H -5.557824 -1.246884 -2.801557

TS Entry A major2 solvent=CH_2Cl_2: The transition state structure leading to the major product in the click reaction between ABSACN Entry A and BnN_3 with CH_2Cl_2 solvent. (Table 4, Table S31)

S 3.656197 -0.793222 0.232588
O 3.465004 -1.500792 -1.046662
O 4.987993 -0.777042 0.851082
N 1.671490 0.611921 -1.907780
C 3.134676 0.919768 0.096515
C 2.180128 1.377653 -0.838450
C 1.740520 2.707559 -0.710351
H 1.023230 3.080229 -1.435393

C 3.659818 1.781024 1.073330
C 1.449717 -2.296157 0.896341
C 0.436448 -1.519313 0.152702
C 0.064536 -0.727710 -0.719394
C 0.241004 0.261408 -1.798874
H -0.344140 1.166624 -1.617423
H -0.089259 -0.151357 -2.757725
H 2.213543 -0.242173 -2.025589
N 2.639049 -1.544717 1.340541
C 3.208070 3.089991 1.173901
H 4.420262 1.408242 1.750144
C 2.229090 3.545046 0.283553
H 3.615621 3.749077 1.933616
H 1.866585 4.566803 0.348359
H 2.481622 -0.952909 2.154035
H 1.796853 -3.115482 0.260224
H 1.038669 -2.745428 1.803157
N -1.444071 -2.412976 0.910427
N -2.187021 -0.934890 -0.660692
N -2.179942 -1.828521 0.214861
C -3.415572 -0.640341 -1.421700
H -3.102362 0.092985 -2.169254
H -3.750975 -1.537038 -1.955788
C -4.519109 -0.079988 -0.552259
C -4.534761 1.277690 -0.211430
C -5.509593 -0.924648 -0.038813
C -6.502965 -0.420525 0.803103
C -5.526253 1.784206 0.628934
C -6.512686 0.934992 1.138637
H -5.502557 -1.980205 -0.300700
H -7.269441 -1.084624 1.193338
H -3.765856 1.938667 -0.604216
H -5.531789 2.840310 0.884901
H -7.285964 1.329677 1.792004

TS Entry A major3 solvent=CH_2Cl_2: The transition state structure leading to the major product in the click reaction between ABSACN Entry A and BnN_3 with CH_2Cl_2 solvent. (Table 4, Table S32)
S 9.540343 6.998999 6.993027
O 10.008428 5.728825 7.574745
O 10.520798 7.945103 6.446565
N 7.785838 4.360703 6.355939
C 8.336438 6.705474 5.692928
C 7.572535 5.523051 5.586684
C 6.549030 5.501198 4.622375
H 5.971288 4.588280 4.512201
C 8.106106 7.795454 4.837800
C 8.166589 7.058015 9.373000
C 7.183102 6.004878 9.049193
C 6.722558 5.051027 8.411948
C 6.713123 4.053716 7.322548
H 5.752243 4.031370 6.800781
H 6.885743 3.050538 7.725420
H 8.667659 4.426914 6.860153
N 8.728715 7.808015 8.228691
C 7.085383 7.746237 3.897276
H 8.732761 8.676647 4.921512
C 6.297009 6.593826 3.803436
H 6.909265 8.592524 3.241050
H 5.496683 6.539260 3.070864
H 8.093592 8.505057 7.842491
H 9.004725 6.604396 9.909431
H 7.742145 7.821490 10.029635
N 5.841657 6.114440 10.838926
N 5.069092 4.285419 9.702919
N 5.156818 5.220930 10.534451
C 3.733028 3.766404 9.345489
H 3.924879 3.060961 8.532673
C 3.046482 3.077949 10.503979
H 3.108191 4.575152 8.947843
C 3.531558 1.858528 10.993220

C	1.943019	3.672141	11.122316
C	1.326475	3.056073	12.214352
C	2.919791	1.243365	12.084502
C	1.816561	1.843205	12.699731
H	1.564593	4.619174	10.746381
H	0.468207	3.526108	12.685804
H	4.392113	1.395128	10.517524
H	3.301891	0.296906	12.456162
H	1.340452	1.362849	13.550078

TS Entry A minor1 solvent=CH_2Cl_2: The transition state structure leading to the minor product in the click reaction between ABSACN Entry A and BnN_3 with CH_2Cl_2 solvent. (Table 4, Table S33)

S	-2.340006	1.795062	-0.120188
O	-1.517891	1.910145	-1.338153
O	-3.285309	2.867898	0.212178
N	-1.852704	-0.943163	-1.775200
C	-3.286181	0.270282	-0.125782
C	-2.906986	-0.885995	-0.842501
C	-3.661647	-2.053055	-0.623985
H	-3.396547	-2.944027	-1.185554
C	-4.401072	0.260554	0.728475
C	0.124602	1.249703	0.977529
C	0.274637	-0.125386	0.463468
C	0.038284	-1.100691	-0.259749
C	-0.700238	-1.764774	-1.355366
H	-1.049088	-2.757661	-1.059970
H	-0.039100	-1.904149	-2.216897
H	-1.524917	-0.006597	-2.001526
N	-1.262095	1.715440	1.171974
C	-5.125433	-0.907415	0.924461
H	-4.687960	1.177049	1.231614
C	-4.739573	-2.071913	0.250636
H	-5.985612	-0.908664	1.586070
H	-5.298985	-2.992553	0.389314
H	-1.718507	1.333268	1.998869
H	0.606260	1.944601	0.282961
H	0.605704	1.383582	1.948981
N	2.078464	-0.849927	1.624267
N	1.587600	-2.587407	0.215139
N	2.165188	-1.926643	0.983319
C	3.312147	-0.101586	1.910984
H	2.958024	0.862335	2.293931
H	3.849897	-0.582462	2.736544
C	4.227210	0.092899	0.719427
C	3.709985	0.388463	-0.548688
C	5.612924	-0.003512	0.884353
C	6.472432	0.202646	-0.196798
C	4.567377	0.591330	-1.630030
C	5.951152	0.502858	-1.456757
H	6.020716	-0.243422	1.863612
H	7.546930	0.122999	-0.055281
H	2.635054	0.442379	-0.692950
H	4.154549	0.818108	-2.609335
H	6.617995	0.659683	-2.300176

TS Entry A minor2 solvent=CH_2Cl_2: The transition state structure leading to the minor product in the click reaction between ABSACN Entry A and BnN_3 with CH_2Cl_2 solvent. (Table 4, Table S34)

S	0.280587	2.121946	-1.310314
O	1.751456	2.078656	-1.389701
O	-0.413611	3.414186	-1.362539
N	1.565765	-0.324615	-2.994211
C	-0.444884	1.108056	-2.602743
C	0.216319	0.016489	-3.208453
C	-0.524587	-0.761229	-4.116707
H	-0.018381	-1.587912	-4.607219
C	-1.771123	1.425641	-2.937275
C	0.761202	0.496267	0.868660
C	1.050472	-0.752795	0.134917
C	1.400219	-1.432201	-0.838016
C	1.776336	-1.588383	-2.259463

H 1.202816 -2.387751 -2.736729
H 2.832973 -1.864025 -2.339932
H 2.051017 0.425224 -2.506320
N -0.119477 1.452802 0.174621
C -2.476003 0.640191 -3.839252
H -2.237098 2.293480 -2.484094
C -1.847287 -0.467342 -4.419399
H -3.500381 0.891986 -4.094211
H -2.383022 -1.087085 -5.132967
H -1.116787 1.263639 0.247196
H 1.704027 1.009211 1.079439
H 0.276673 0.319620 1.831981
N 1.189869 -2.258291 1.819021
N 1.747459 -3.455609 -0.054509
N 1.453599 -3.213861 1.047567
C 0.164751 -2.475214 2.860500
H 0.580500 -3.110865 3.650628
H 0.011420 -1.484520 3.298904
C -1.127032 -3.051314 2.327259
C -1.552398 -4.324093 2.719624
C -1.896725 -2.330100 1.405273
C -3.074614 -2.867452 0.890769
C -2.733875 -4.866173 2.207277
C -3.495776 -4.139215 1.291894
H -1.564978 -1.347863 1.081032
H -3.663540 -2.296718 0.178690
H -0.956509 -4.893300 3.429456
H -3.053938 -5.856125 2.520523
H -4.413597 -4.559487 0.890364

TS Entry A minor3 solvent=CH_2Cl_2: The transition state structure leading to the minor product in the click reaction between ABSACN Entry A and BnN_3 with CH_2Cl_2 solvent. (Table 4, Table S35)

S 0.323411 2.148906 -1.339830
O 1.795916 2.105230 -1.376444
O -0.362177 3.445397 -1.398498
N 1.647002 -0.271166 -3.039766
C -0.377123 1.145202 -2.653517
C 0.295305 0.060315 -3.258931
C -0.438185 -0.723077 -4.168731
H 0.073750 -1.544641 -4.660840
C -1.707214 1.449597 -2.986149
C 0.753373 0.502310 0.821610
C 1.077844 -0.725797 0.068951
C 1.451157 -1.399264 -0.897664
C 1.862696 -1.538410 -2.310930
H 1.313261 -2.342327 -2.808788
H 2.925678 -1.795913 -2.371050
H 2.121916 0.478629 -2.541771
N -0.133999 1.452968 0.124413
C -2.405046 0.657149 -3.887095
H -2.181184 2.311474 -2.530260
C -1.764577 -0.443320 -4.468535
H -3.433459 0.896581 -4.137840
H -2.295554 -1.069809 -5.179335
H -1.109351 1.159554 0.093499
H 1.680788 1.030407 1.058531
H 0.253684 0.296116 1.770557
N 1.062413 -2.265029 1.720807
N 1.697860 -3.466759 -0.119643
N 1.345779 -3.221498 0.966806
C 0.108842 -2.451609 2.835129
H -0.692751 -1.722026 2.676593
H -0.340673 -3.447289 2.776506
C 0.761096 -2.223318 4.180140
C 0.474742 -1.075624 4.924792
C 1.666942 -3.161545 4.692077
C 2.268785 -2.958111 5.933063
C 1.077529 -0.867562 6.168124
C 1.973505 -1.809819 6.675316
H 1.899604 -4.053841 4.115302

H 2.966141 -3.694226 6.324090
H -0.230204 -0.344469 4.535272
H 0.844504 0.027711 6.738155
H 2.441741 -1.651314 7.642721

TS Entry B major1 solvent=CH_2Cl_2: The transition state structure leading to the major product in the click reaction between ABSACN Entry B and BnN_3 with CH_2Cl_2 solvent. (Table 4, Table S39)

S 1.554519 -0.666161 1.722199
O 0.275662 -1.042876 2.346806
O 2.745504 -0.558215 2.564065
O 1.815204 2.552819 -0.426261
O 3.368439 1.043188 0.291198
N 1.235012 0.883905 1.026504
N -0.476835 -2.383487 -0.074969
N -3.007300 0.603681 -1.976608
N -2.892307 1.758141 -1.496565
N -2.237644 2.430656 -0.810101
C 1.897549 -1.750677 0.346931
C 0.874386 -2.330041 -0.444150
C 1.273496 -2.958339 -1.638812
H 0.508157 -3.418273 -2.256685
C 2.611040 -3.052425 -1.998755
H 2.878078 -3.554293 -2.924567
C 3.613543 -2.553241 -1.158143
H 4.661335 -2.669787 -1.415764
C 3.250551 -1.900889 0.011921
H 3.995501 -1.478735 0.672176
C -0.160243 1.387812 1.016295
H -0.077000 2.462372 0.864612
H -0.589256 1.213170 2.005294
C -0.992633 0.734873 -0.006493
C -1.415251 -0.244019 -0.629965
C -1.455117 -1.687223 -0.931676
H -2.447360 -2.098851 -0.721835
H -1.237030 -1.889340 -1.983775
C 2.152855 1.577691 0.220507
C 4.508214 1.648110 -0.449711
C 5.686042 0.763178 -0.045882
H 5.561069 -0.256517 -0.419972
H 5.789232 0.731530 1.043137
H 6.607972 1.170644 -0.471981
C 4.725113 3.082112 0.033330
H 3.888479 3.725657 -0.239932
H 5.637445 3.477583 -0.425404
H 4.852650 3.100155 1.120914
C 4.240850 1.553185 -1.950948
H 3.405607 2.189715 -2.247394
H 4.020374 0.517437 -2.230066
H 5.136054 1.871613 -2.495854
H -0.605923 -2.087895 0.889251
C -4.380470 0.043439 -2.067868
H -4.238356 -0.976484 -2.430816
C -5.109305 0.059090 -0.744801
H -4.934097 0.592510 -2.838219
C -5.717435 1.237340 -0.292298
C -5.138973 -1.076810 0.071933
C -5.757189 -1.036993 1.322909
C -6.334429 1.281334 0.957907
C -6.353171 0.144110 1.769876
H -4.684502 -2.001603 -0.275179
H -5.774144 -1.927391 1.945399
H -5.709957 2.123847 -0.922127
H -6.803735 2.201094 1.296107
H -6.834923 0.178141 2.743181

TS Entry B major2 solvent=CH_2Cl_2: The transition state structure leading to the major product in the click reaction between ABSACN Entry B and BnN_3 with CH_2Cl_2 solvent. (Table 4, Table S40)

S 9.462307 7.120171 6.939981
O 9.908674 5.832298 7.496167
O 10.463644 8.109436 6.543281

N	7.647140	4.540270	6.306718
C	8.365140	6.818474	5.561400
C	7.547051	5.664078	5.470385
C	6.623748	5.620561	4.409613
H	6.002958	4.734607	4.311484
C	8.316335	7.824037	4.584340
C	8.195543	6.946108	9.390021
C	7.156551	5.943651	9.099804
C	6.595237	5.104769	8.387966
C	6.506141	4.240358	7.195168
H	5.568732	4.388544	6.651194
H	6.551910	3.185047	7.482892
H	8.501693	4.570500	6.856883
N	8.508997	7.796105	8.212863
C	7.410425	7.737152	3.535745
H	8.991529	8.665312	4.666987
C	6.545060	6.638404	3.468980
H	7.379887	8.511490	2.775698
H	5.829616	6.557470	2.655277
C	7.988582	9.101376	8.209045
H	9.111208	6.442792	9.706521
H	7.899992	7.641327	10.173432
O	8.173914	9.703094	7.038178
O	7.435443	9.565864	9.190343
C	7.849306	11.147355	6.868967
C	6.351828	11.375834	7.081862
C	8.713889	11.974097	7.820683
C	8.234578	11.409560	5.415961
H	5.768528	10.707409	6.439813
H	6.067138	11.209990	8.120905
H	6.107514	12.408669	6.810622
H	8.467090	11.769964	8.863898
H	9.775054	11.761580	7.654340
H	8.543123	13.038656	7.626768
H	9.292558	11.186139	5.246494
H	7.628530	10.800776	4.738172
H	8.060094	12.464377	5.181101
N	6.021663	5.859895	11.022872
N	5.027894	4.208712	9.781806
N	5.268342	5.032414	10.693371
C	3.642282	3.737918	9.573880
H	3.240719	3.389177	10.532481
C	2.731888	4.772529	8.946350
H	3.730371	2.865729	8.922402
C	2.340901	5.909845	9.666465
C	2.259484	4.601344	7.640802
C	1.408488	5.546738	7.061805
C	1.497665	6.858653	9.088925
C	1.027687	6.678212	7.784325
H	2.547757	3.716778	7.077367
H	1.043447	5.396732	6.049357
H	2.691467	6.052047	10.685916
H	1.201293	7.735479	9.658379
H	0.365947	7.415023	7.336991

TS Entry B major3 solvent=CH_2Cl_2: The transition state structure leading to the major product in the click reaction between ABSACN Entry B and BnN_3 with CH_2Cl_2 solvent. (Table 4, Table S41)

S	9.453203	7.131410	6.984703
O	9.907316	5.850442	7.550216
O	10.447004	8.132184	6.598713
N	7.696312	4.519417	6.323992
C	8.373203	6.815474	5.596521
C	7.575980	5.647689	5.496075
C	6.652826	5.597456	4.435404
H	6.047018	4.702141	4.329693
C	8.306184	7.828458	4.628238
C	8.144474	6.945158	9.412100
C	7.121081	5.931901	9.102846
C	6.600958	5.067769	8.388994

C	6.555891	4.191361	7.202495
H	5.622112	4.302746	6.643924
H	6.631878	3.140492	7.500103
H	8.545968	4.565284	6.880707
N	8.474090	7.797899	8.242253
C	7.400881	7.735624	3.579984
H	8.964849	8.681415	4.719829
C	6.555061	6.622346	3.504201
H	7.355158	8.516740	2.827653
H	5.839921	6.536268	2.690841
C	7.937457	9.095515	8.222954
H	9.058112	6.448942	9.745148
H	7.828033	7.637251	10.190007
O	8.135025	9.690970	7.050986
O	7.362877	9.559493	9.192019
C	7.813351	11.133404	6.870905
C	6.313694	11.361739	7.063858
C	8.666961	11.961234	7.831088
C	8.217812	11.389473	5.422103
H	5.739886	10.687881	6.418940
H	6.017352	11.200902	8.100359
H	6.070899	12.392438	6.783602
H	8.408631	11.756842	8.871486
H	9.729690	11.747808	7.676267
H	8.498242	13.025613	7.635204
H	9.277482	11.163179	5.267677
H	7.619233	10.779217	4.739087
H	8.048458	12.443609	5.181045
N	5.886723	5.899317	10.960782
N	4.998868	4.186013	9.727847
N	5.166093	5.046771	10.623884
C	3.635729	3.689596	9.450454
H	3.767285	2.982336	8.627242
C	2.992120	3.012418	10.641891
H	3.003206	4.509149	9.088080
C	3.508727	1.811595	11.146631
C	1.882521	3.592940	11.263633
C	1.289371	2.981237	12.371199
C	2.920248	1.200975	12.253452
C	1.808834	1.785732	12.869435
H	1.479560	4.525970	10.877460
H	0.425306	3.440539	12.843470
H	4.374817	1.358629	10.670668
H	3.325659	0.268281	12.636246
H	1.350000	1.307978	13.730795

TS Entry B minor1 solvent=CH_2Cl_2: The transition state structure leading to the minor product in the click reaction between ABSACN Entry B and BnN_3 with CH_2Cl_2 solvent. (Table 4, Table S42)

S	0.349068	2.097670	-1.042362
O	1.820319	2.047252	-1.082305
O	-0.318089	3.397842	-1.007766
N	1.674271	-0.347359	-2.681249
C	-0.318259	1.137051	-2.390268
C	0.344020	0.014706	-2.947025
C	-0.380274	-0.747894	-3.883242
H	0.113035	-1.603379	-4.334451
C	-1.594855	1.520734	-2.827179
C	1.012677	0.477340	1.090511
C	1.257134	-0.807126	0.412014
C	1.524031	-1.505679	-0.570870
C	1.881623	-1.642046	-1.997043
H	1.289630	-2.413752	-2.495086
H	2.933579	-1.927738	-2.101668
H	2.153489	0.384783	-2.162879
N	-0.048711	1.262434	0.419006
C	-2.272922	0.757891	-3.766162
H	-2.039292	2.412927	-2.408662
C	-1.665484	-0.397471	-4.273180
H	-3.259103	1.060153	-4.104015
H	-2.184915	-1.008373	-5.005858

C	-1.361100	1.034385	0.866807
H	1.925032	1.075451	1.085483
H	0.676246	0.368330	2.118650
O	-2.255157	1.653963	0.103313
O	-1.580200	0.348915	1.849818
C	-3.707688	1.583250	0.421069
C	-4.178086	0.133109	0.327665
C	-3.956248	2.207978	1.793840
C	-4.335055	2.434000	-0.680712
H	-3.911546	-0.289606	-0.646677
H	-3.737055	-0.478350	1.115909
H	-5.268721	0.104290	0.426682
H	-3.498041	1.618453	2.588947
H	-3.554045	3.225728	1.825687
H	-5.034698	2.260950	1.974386
H	-3.909599	3.442463	-0.679103
H	-4.174390	1.981053	-1.662610
H	-5.413361	2.510778	-0.511526
N	0.671369	-2.362886	1.950521
N	1.424696	-3.637497	0.193180
N	1.083832	-3.320540	1.260251
C	1.063647	-2.284347	3.374792
H	0.532878	-1.409849	3.757033
H	0.672089	-3.161916	3.900715
C	2.556560	-2.146148	3.561055
C	3.373385	-3.283483	3.579033
C	3.148322	-0.881782	3.664917
C	4.533386	-0.753621	3.782141
C	4.758670	-3.158990	3.695489
C	5.341459	-1.892715	3.796154
H	2.522067	0.006771	3.663529
H	4.979233	0.233928	3.863841
H	2.922801	-4.271256	3.508523
H	5.381706	-4.049025	3.713979
H	6.419702	-1.795626	3.889426

TS Entry B minor2 solvent=CH_2Cl_2: The transition state structure leading to the minor product in the click reaction between ABSACN Entry B and BnN_3 with CH_2Cl_2 solvent. (Table 4, Table S43)

S	0.316488	1.901986	-1.418746
O	1.755912	1.698747	-1.653293
O	-0.231962	3.254970	-1.508215
N	1.163249	-0.885279	-2.775657
C	-0.630111	0.822719	-2.478597
C	-0.162927	-0.447680	-2.896197
C	-1.091940	-1.285206	-3.543077
H	-0.753181	-2.260314	-3.879145
C	-1.919628	1.267344	-2.803563
C	1.199113	0.619624	0.867018
C	1.349867	-0.757455	0.366409
C	1.431159	-1.633493	-0.500154
C	1.416714	-2.056873	-1.914388
H	0.650758	-2.816008	-2.095192
H	2.376642	-2.502388	-2.192333
H	1.774798	-0.123552	-2.492256
N	0.085223	1.333418	0.195602
C	-2.799822	0.430906	-3.474654
H	-2.212918	2.265518	-2.508879
C	-2.386017	-0.863285	-3.814322
H	-3.795596	0.778755	-3.730442
H	-3.067492	-1.534667	-4.329194
C	-1.163112	1.271576	0.843731
H	2.117960	1.181881	0.690570
H	0.965445	0.661648	1.928341
O	-2.126893	1.847846	0.128307
O	-1.275034	0.751412	1.935704
C	-3.541032	1.762351	0.590634
C	-3.977840	0.299329	0.543657
C	-3.688705	2.378069	1.982727
C	-4.288375	2.599040	-0.444639
H	-3.827389	-0.110001	-0.461176

H -3.422861 -0.301809 1.266283
H -5.044489 0.235501 0.785405
H -3.204233 1.765945 2.742853
H -3.260312 3.385992 2.003144
H -4.755658 2.458446 2.218073
H -3.898057 3.620736 -0.472049
H -4.202302 2.160231 -1.441069
H -5.349479 2.639267 -0.180616
N 1.862377 -1.943812 2.251420
N 1.983532 -3.507742 0.577429
N 1.945088 -3.033008 1.642644
C 1.241801 -1.894621 3.584477
H 1.617236 -2.735668 4.180970
H 1.621432 -0.983664 4.054143
C -0.276246 -1.873946 3.574266
C -0.946453 -1.194744 4.597521
C -1.024476 -2.531135 2.593186
C -2.419269 -2.517576 2.642890
C -2.339899 -1.180320 4.648597
C -3.082027 -1.843264 3.669996
H -0.528913 -3.046406 1.777549
H -2.988221 -3.031358 1.872631
H -0.373287 -0.666711 5.356556
H -2.844774 -0.643184 5.447023
H -4.168134 -1.829212 3.702996

TS Entry B minor3 solvent=CH_2Cl_2: The transition state structure leading to the minor product in the click reaction between ABSACN Entry B and BnN_3 with CH_2Cl_2 solvent. (Table 4, Table S44)
S 0.204564 2.069086 -1.255349
O 1.673183 2.067273 -1.357884
O -0.501181 3.345357 -1.159612
N 1.548639 -0.292129 -2.997931
C -0.479597 1.127389 -2.610533
C 0.207442 0.053564 -3.231209
C -0.500561 -0.672028 -4.208434
H 0.016166 -1.485033 -4.709687
C -1.769487 1.500195 -3.018706
C 1.026157 0.441002 0.817866
C 1.290716 -0.822522 0.105093
C 1.538698 -1.494864 -0.901539
C 1.799738 -1.598276 -2.351430
H 1.175408 -2.362698 -2.821490
H 2.842047 -1.882053 -2.531671
H 2.028720 0.437563 -2.477384
N -0.098816 1.183429 0.202244
C -2.427839 0.778619 -4.004034
H -2.238495 2.355447 -2.551039
C -1.792782 -0.328344 -4.580733
H -3.420272 1.077080 -4.326514
H -2.295387 -0.901818 -5.354305
C -1.370260 0.987170 0.760276
H 1.911123 1.078153 0.778349
H 0.753109 0.304743 1.861926
O -2.324350 1.546047 0.024964
O -1.517856 0.367095 1.801274
C -3.719068 1.658069 0.535870
C -4.319691 0.265763 0.724932
C -3.716902 2.480698 1.823965
C -4.425462 2.410586 -0.589033
H -4.220916 -0.322115 -0.193790
H -3.836143 -0.268082 1.543627
H -5.386388 0.367534 0.952504
H -3.190112 1.963189 2.627774
H -3.244819 3.453892 1.654439
H -4.750800 2.653234 2.141198
H -3.940592 3.374540 -0.771836
H -4.417756 1.825634 -1.513689
H -5.467032 2.593256 -0.306358
N 1.240221 -2.377836 1.733007
N 1.687975 -3.616433 -0.147338

N 1.404528 -3.335926 0.948251
C 0.210984 -2.470300 2.795853
H -0.552450 -1.723875 2.553803
H -0.260321 -3.457368 2.761500
C 0.794901 -2.199219 4.163451
C 0.416481 -1.061473 4.882958
C 1.713438 -3.092495 4.731442
C 2.235094 -2.858188 6.002784
C 0.937592 -0.824324 6.158641
C 1.845556 -1.722789 6.721186
H 2.016463 -3.976167 4.174420
H 2.943571 -3.559573 6.435686
H -0.293076 -0.364452 4.443118
H 0.634866 0.061524 6.710665
H 2.250277 -1.541135 7.713277

TS Entry D major1 solvent=CH_2Cl_2: The transition state structure leading to the major product in the click reaction between ABSACN Entry D and BnN_3 with CH_2Cl_2 solvent. (Table 4, Table S48)

S 2.891665 -0.987748 -1.180600
O 1.993035 -0.334767 -2.137058
O 4.253303 -1.354919 -1.592366
N 1.037290 1.426098 0.079645
C 3.119233 0.035918 0.298497
C 2.253678 1.059644 0.732338
C 2.581654 1.757257 1.902029
H 1.917963 2.554795 2.222273
C 4.282072 -0.252454 1.026908
C 0.693310 -2.585828 -0.833811
C -0.109296 -1.636670 -0.035289
C -0.367392 -0.512375 0.404515
C -0.212748 0.923359 0.670088
H -0.212469 1.122048 1.747158
H -1.022852 1.494030 0.211228
C 0.967858 2.283883 -1.002291
N 2.151822 -2.419658 -0.722722
C 4.582836 0.447132 2.192066
H 4.960397 -1.014771 0.662313
C 3.728243 1.457303 2.632540
H 5.487434 0.210278 2.743348
H 3.958181 2.017341 3.533893
H 2.566231 -2.810256 0.121023
H 0.427395 -2.475782 -1.888806
H 0.494241 -3.623648 -0.557347
N -1.777307 -2.886463 0.559192
N -2.310339 -0.894806 1.533348
N -2.398008 -2.080318 1.138625
C -3.526853 -0.161257 1.942172
H -4.055633 -0.747655 2.702420
H -3.157636 0.745372 2.426796
C -4.442445 0.189989 0.789375
C -5.360166 -0.747982 0.297657
C -4.368799 1.450014 0.185592
C -5.194131 1.770453 -0.894065
C -6.184963 -0.431801 -0.782643
C -6.101763 0.827856 -1.382291
H -3.668942 2.188433 0.570064
H -5.130093 2.754424 -1.350429
H -5.433709 -1.727393 0.765278
H -6.896794 -1.164968 -1.152642
H -6.745866 1.076052 -2.221547
O -0.112479 2.580906 -1.505965
C 2.275208 2.844939 -1.520750
H 2.959078 3.128595 -0.716800
H 2.771377 2.094163 -2.143788
H 2.046205 3.717392 -2.135159

TS Entry D minor1 solvent=CH_2Cl_2: The transition state structure leading to the minor product in the click reaction between ABSACN Entry D and BnN_3 with CH_2Cl_2 solvent. (Table 4, Table S49)

S 13.141031 -0.830613 15.276523

O 14.281663 -0.669095 16.180976
O 11.771081 -0.681497 15.783475
N 13.234252 -2.387866 14.659673
N 15.666972 0.886117 14.090174
N 17.721647 -2.172641 12.034815
N 17.378842 -3.291541 12.067831
N 16.475555 -4.005651 12.557985
C 13.237844 0.316954 13.878082
C 14.391234 1.009933 13.465141
C 14.301793 1.847586 12.346475
H 15.192663 2.388296 12.041541
C 13.107300 2.000651 11.648075
H 13.067179 2.659481 10.786194
C 11.966471 1.317599 12.070042
H 11.024525 1.435464 11.543738
C 12.031505 0.486972 13.184732
H 11.139896 -0.021964 13.532048
C 14.533873 -3.075720 14.548442
H 14.301521 -4.049294 14.112487
H 14.904059 -3.250733 15.562971
C 15.559321 -2.345923 13.779707
C 16.244165 -1.380227 13.428078
C 16.647620 0.035965 13.403427
H 17.598702 0.174369 13.921032
H 16.770521 0.383960 12.372414
H 12.628901 -2.504694 13.849255
C 16.597774 -5.474759 12.565225
H 16.512200 -5.849046 11.538248
H 15.713742 -5.818843 13.109164
C 17.862911 -5.981287 13.218789
C 18.978507 -6.297874 12.435271
H 18.919128 -6.202705 11.353394
C 20.159387 -6.743345 13.032496
H 21.017611 -6.992119 12.414182
C 20.234708 -6.871050 14.421119
H 21.151703 -7.222265 14.886497
C 19.125081 -6.554578 15.210023
H 19.177377 -6.657382 16.290473
C 17.946835 -6.108494 14.610298
H 17.083295 -5.864869 15.224947
C 16.024858 1.537838 15.252727
O 17.138807 1.391114 15.750122
C 14.984983 2.450476 15.868405
H 15.498989 3.158366 16.520914
H 14.405374 2.992969 15.117609
H 14.293175 1.855435 16.472457

TS Entry D major1 solvent=CH_3CN: The transition state structure leading to the major product in the click reaction between ABSACN Entry D and BnN_3 with CH_3CN solvent. (Table 4, Table S55)

S 2.700035 -0.924760 -1.263567
O 1.773083 -0.248167 -2.177013
O 4.051033 -1.266955 -1.730094
N 0.889253 1.452353 0.098520
C 2.961085 0.044190 0.244243
C 2.108426 1.056868 0.724611
C 2.448411 1.705651 1.917583
H 1.791565 2.493551 2.273543
C 4.128809 -0.280255 0.949018
C 0.511233 -2.523281 -0.902500
C -0.239347 -1.604282 -0.025018
C -0.489601 -0.496302 0.457195
C -0.349713 0.944686 0.703047
H -0.344466 1.159388 1.776809
H -1.170381 1.496940 0.240616
C 0.815635 2.314734 -0.975660
N 1.977160 -2.378123 -0.839063
C 4.442638 0.371704 2.138165
H 4.799135 -1.031282 0.548350
C 3.598028 1.368768 2.626303
H 5.349794 0.107965 2.672570

H 3.839168 1.892201 3.546193
H 2.401811 -2.780426 -0.005593
H 0.210168 -2.354151 -1.939826
H 0.311857 -3.572364 -0.673484
N -1.812183 -2.923283 0.805479
N -2.206482 -0.927270 1.853360
N -2.341739 -2.105982 1.448126
C -3.415863 -0.112831 2.108906
H -4.039991 -0.627972 2.847727
H -3.044387 0.802688 2.573691
C -4.192415 0.198785 0.850367
C -5.049797 -0.759045 0.293242
C -4.042363 1.433359 0.209646
C -4.734930 1.709537 -0.971153
C -5.742819 -0.486212 -0.886372
C -5.585374 0.748807 -1.521752
H -3.391548 2.188584 0.643697
H -4.612560 2.674010 -1.455917
H -5.180879 -1.718822 0.787794
H -6.409302 -1.234458 -1.306607
H -6.126911 0.961879 -2.439179
O -0.267020 2.623771 -1.468618
C 2.124870 2.869397 -1.495997
H 2.817769 3.128127 -0.691274
H 2.606087 2.123983 -2.136152
H 1.903485 3.756014 -2.092311

TS Entry D minor1 solvent=CH_3CN: The transition state structure leading to the minor product in the click reaction between ABSACN Entry D and BnN_3 with CH_3CN solvent. (Table 4, Table S56)
S 13.139461 -0.825110 15.274333
O 14.280336 -0.662257 16.179112
O 11.770785 -0.675863 15.786208
N 13.231869 -2.382581 14.661007
N 15.662073 0.893522 14.087768
N 17.732638 -2.167889 12.053387
N 17.393992 -3.288344 12.090274
N 16.493330 -4.003067 12.582373
C 13.233293 0.322627 13.876857
C 14.385675 1.016882 13.463081
C 14.295360 1.854293 12.344662
H 15.185404 2.395770 12.038769
C 13.100184 2.005879 11.646902
H 13.059197 2.664334 10.784882
C 11.960384 1.321519 12.069292
H 11.018226 1.437762 11.543167
C 12.026537 0.490772 13.183892
H 11.135468 -0.019719 13.530064
C 14.530863 -3.071855 14.547932
H 14.296898 -4.042774 14.107373
H 14.900785 -3.252504 15.561552
C 15.557303 -2.340040 13.782317
C 16.241601 -1.374092 13.430139
C 16.642132 0.042908 13.400496
H 17.594970 0.184532 13.913946
H 16.760593 0.388765 12.368183
H 12.624270 -2.502913 13.852582
C 16.606141 -5.473254 12.574201
H 16.517850 -5.835749 11.543424
H 15.721097 -5.817978 13.115861
C 17.870132 -5.990604 13.221797
C 18.984242 -6.302780 12.434223
H 18.923898 -6.198257 11.353093
C 20.165076 -6.755492 13.026679
H 21.022032 -7.000905 12.405209
C 20.241726 -6.895474 14.414378
H 21.158685 -7.252551 14.875699
C 19.133416 -6.583728 15.207237
H 19.186640 -6.695936 16.286927
C 17.955182 -6.130370 14.612327
H 17.092803 -5.890255 15.230011

C 16.018932 1.544905 15.249065
O 17.132486 1.396595 15.749141
C 14.980049 2.459418 15.863323
H 15.494020 3.167129 16.516112
H 14.402156 3.002320 15.111656
H 14.286394 1.865877 16.466779

BnN_3 solvent=CH_2Cl_2: The optimized ground state structure (GS1) representing the total energy of the reactants (each optimized ABSACN and optimized BnN_3) under dichloromethane (CH_2Cl_2) solvent conditions. (Table 4, Table S28)

N 0.844744 -2.849386 3.688997
N 0.437581 -3.435167 1.425997
N 0.671319 -3.188466 2.611162
C 0.925931 -4.768592 0.946937
H 0.299864 -5.555244 1.383813
H 0.739893 -4.747757 -0.128309
C 2.383883 -5.008874 1.252528
C 2.759762 -5.895775 2.266707
C 3.376983 -4.310899 0.551370
C 4.723623 -4.506195 0.852980
C 4.109369 -6.096566 2.567781
C 5.092481 -5.400896 1.862752
H 3.088922 -3.613905 -0.231729
H 5.486185 -3.963347 0.301265
H 1.993893 -6.437726 2.816404
H 4.390114 -6.792821 3.353138
H 6.142306 -5.555171 2.096566

BnN_3 solvent=CH_3CN: The optimized ground state structure (GS1) representing the total energy of the reactants (optimized ABSACN Entry D and optimized BnN_3) under dichloromethane (CH_3CN) solvent conditions. (Table 4, Table S54)

N 0.785047 -2.857460 3.664170
N 0.413212 -3.435427 1.393477
N 0.627857 -3.192008 2.582374
C 0.919432 -4.764274 0.917761
H 0.291240 -5.556252 1.341550
H 0.751168 -4.741237 -0.160378
C 2.373289 -4.994983 1.248230
C 2.737979 -5.886623 2.262382
C 3.373030 -4.285496 0.568391
C 4.715942 -4.475867 0.889405
C 4.084019 -6.082771 2.582623
C 5.074024 -5.376812 1.897721
H 3.093308 -3.584938 -0.214625
H 5.483788 -3.925136 0.353149
H 1.966783 -6.437245 2.795604
H 4.356491 -6.783963 3.366540
H 6.121084 -5.528282 2.145488

GS2 Entry A major solvent=CH_2Cl_2: The ground state structure (GS2) of the major product obtained from the click reaction between ABSACN Entry A and BnN_3 with CH_2Cl_2 as the solvent. (Table 4, Table S36)

S -0.134804 -0.082424 0.084163
O 1.220628 0.271356 -0.380447
O -1.121074 0.968585 0.354284
N 1.315555 -2.300293 -1.621435
C -0.854617 -1.214513 -1.100402
C -0.079019 -2.204459 -1.745216
C -0.761557 -3.138528 -2.543084
H -0.184267 -3.894139 -3.067576
C -2.241616 -1.144053 -1.280478
C 1.161107 -1.854779 1.746338
C 0.824323 -3.258076 1.344854
C 1.123159 -3.963850 0.189700
C 1.841006 -3.556158 -1.068125
H 1.778382 -4.354106 -1.812453
H 2.906987 -3.405503 -0.873168
H 1.721042 -1.480578 -1.182052
N 0.081279 -0.865820 1.544264
C -2.890546 -2.078847 -2.078621

H -2.795827 -0.358154 -0.779785
C -2.141850 -3.083721 -2.700020
H -3.965832 -2.025497 -2.214400
H -2.635243 -3.820437 -3.327643
H -0.811723 -1.132208 1.956609
H 2.044552 -1.478940 1.228154
H 1.382899 -1.835578 2.817202
N 0.071127 -4.054970 2.157100
N 0.502457 -5.159579 0.372011
N -0.121159 -5.209212 1.571614
C 0.524292 -6.343328 -0.484781
H -0.216384 -7.021162 -0.052366
H 0.172312 -6.062131 -1.481471
C 1.898463 -6.975516 -0.545659
C 2.607658 -7.226397 0.635622
C 2.465231 -7.322009 -1.775004
C 3.725030 -7.924222 -1.825862
C 3.866286 -7.822900 0.585226
C 4.426419 -8.176601 -0.646301
H 1.920796 -7.124873 -2.695561
H 4.157620 -8.189437 -2.786584
H 2.171484 -6.950527 1.592405
H 4.410144 -8.013183 1.506262
H 5.407161 -8.642491 -0.684068

GS2 Entry A minor solvent=CH_2Cl_2: The ground state structure (GS2) of the minor product obtained from the click reaction between ABSACN Entry A and BnN_3 with CH_2Cl_2 as the solvent. (Table 4, Table S37)
S -0.169201 -0.058874 -0.131854
O 1.217886 0.189509 -0.568926
O -1.104538 1.058545 0.026116
N 1.194361 -2.493091 -1.563547
C -0.919842 -1.255735 -1.222558
C -0.188462 -2.362195 -1.713511
C -0.914781 -3.363408 -2.383510
H -0.380020 -4.226371 -2.766755
C -2.297010 -1.128176 -1.448624
C 1.010924 -1.723519 1.720334
C 0.691805 -3.162555 1.439886
C 1.000198 -4.009333 0.385051
C 1.704312 -3.705430 -0.908671
H 1.599743 -4.574947 -1.564242
H 2.777677 -3.553807 -0.755886
H 1.637628 -1.644213 -1.230747
N -0.038136 -0.738681 1.397393
C -2.983745 -2.121326 -2.135283
H -2.813091 -0.254259 -1.067373
C -2.284745 -3.248299 -2.583623
H -4.050331 -2.024844 -2.310795
H -2.810783 -4.038511 -3.111835
H -0.959166 -0.924997 1.788348
H 1.905002 -1.422739 1.173094
H 1.239076 -1.597074 2.782880
N -0.018267 -3.953756 2.293397
N 0.472830 -5.237561 0.649026
N -0.137314 -5.206606 1.809453
C -0.551512 -3.636259 3.616866
C 0.538890 -3.412078 4.643911
H -1.204262 -2.761327 3.538428
H -1.180545 -4.490019 3.882634
C 1.614222 -4.304542 4.736065
C 0.465870 -2.327583 5.523010
C 1.451342 -2.141354 6.495411
C 2.599777 -4.116113 5.703664
C 2.519358 -3.034972 6.586756
H -0.365680 -1.630934 5.450180
H 1.386391 -1.296326 7.174900
H 1.674633 -5.143988 4.048687
H 3.431115 -4.812239 5.769501
H 3.288389 -2.888534 7.339877

GS2 Entry B major solvent=CH_2Cl_2: The ground state structure (GS2) of the major product obtained from the click reaction between ABSACN Entry B and BnN_3 with CH_2Cl_2 as the solvent. (Table 4, Table S45)

S 13.898820 -1.252057 15.228777
O 15.209606 -1.177817 15.903621
O 12.697024 -1.362341 16.052467
O 12.972526 -4.280546 13.027105
O 11.886652 -2.344836 13.561411
N 14.024193 -2.641248 14.224007
N 16.109970 -0.089915 13.451286
N 16.708939 -2.484066 10.676659
N 16.295122 -3.765225 10.537497
N 15.770537 -4.127538 11.676325
C 13.744214 0.088865 14.080833
C 14.829528 0.403361 13.228329
C 14.567262 1.291906 12.169412
H 15.372388 1.562981 11.494572
C 13.309798 1.861404 12.008229
H 13.145631 2.552117 11.186224
C 12.267787 1.583924 12.901930
H 11.296459 2.052277 12.782585
C 12.487844 0.683245 13.935898
H 11.696452 0.407358 14.621845
C 15.340456 -3.278176 13.968677
H 15.220592 -4.348023 14.144648
H 16.030007 -2.889838 14.715381
C 15.849530 -3.094097 12.567769
C 16.465603 -2.021634 11.930901
C 16.913612 -0.653429 12.382156
H 17.949462 -0.708699 12.734819
H 16.926233 0.031927 11.531293
C 12.916831 -3.184931 13.550687
C 10.561614 -2.743924 13.021910
C 9.700341 -1.523165 13.336008
H 10.089283 -0.636964 12.826120
H 9.686395 -1.332197 14.413572
H 8.674550 -1.699497 12.997770
C 10.056480 -3.979026 13.765218
H 10.666038 -4.855249 13.539443
H 9.023779 -4.182787 13.462299
H 10.068817 -3.801849 14.845676
C 10.681319 -2.961997 11.514615
H 11.313711 -3.822471 11.289611
H 11.103951 -2.072160 11.035705
H 9.684350 -3.136531 11.095074
H 16.235801 -0.537304 14.351681
C 17.433243 -1.829601 9.589449
H 17.295531 -2.479808 8.721597
H 16.953615 -0.871582 9.369549
C 18.898598 -1.641890 9.921723
C 19.646499 -2.714969 10.423587
C 19.516567 -0.403699 9.728306
C 20.872875 -0.239942 10.020480
C 20.998274 -2.550759 10.720316
C 21.615588 -1.312682 10.516311
H 18.936960 0.434338 9.347927
H 21.344364 0.726664 9.866923
H 19.165582 -3.676750 10.583143
H 21.572191 -3.387842 11.109063
H 22.669864 -1.186665 10.747164

GS2 Entry B minor solvent=CH_2Cl_2: The ground state structure (GS2) of the minor product obtained from the click reaction between ABSACN Entry B and BnN_3 with CH_2Cl_2 as the solvent. (Table 4, Table S46)

S 13.308185 -1.049401 15.418815
O 14.415149 -0.679739 16.322716
O 12.030582 -1.437563 16.011440
O 13.624569 -4.037309 12.975965
O 11.967478 -2.533832 13.415748
N 13.943610 -2.369772 14.498709
N 15.486706 0.451471 13.924839
N 17.340231 -1.794274 11.607909

N	17.342247	-3.104817	11.549058
N	16.631941	-3.556822	12.595551
C	13.071145	0.205964	14.196984
C	14.196897	0.691213	13.486970
C	13.935314	1.473974	12.345516
H	14.767157	1.862390	11.767738
C	12.632661	1.799185	11.988340
H	12.467936	2.420545	11.112505
C	11.537710	1.368564	12.750114
H	10.527202	1.650782	12.473660
C	11.762228	0.553221	13.851340
H	10.942151	0.148452	14.431931
C	15.368037	-2.752113	14.617779
H	15.413727	-3.803059	14.906468
H	15.777535	-2.179056	15.448018
C	16.170029	-2.526233	13.364771
C	16.642120	-1.395030	12.707710
C	16.580830	0.074292	13.047681
H	17.516075	0.371154	13.537373
H	16.530396	0.643249	12.116403
C	13.171706	-3.069746	13.562388
C	11.006018	-3.058523	12.408040
C	9.784019	-2.170074	12.624369
H	10.020187	-1.126174	12.402558
H	9.436744	-2.241997	13.659698
H	8.975897	-2.492785	11.960549
C	10.675187	-4.518506	12.714089
H	11.542298	-5.164978	12.574925
H	9.877305	-4.849912	12.041031
H	10.315355	-4.618468	13.743525
C	11.597681	-2.854467	11.014750
H	12.473363	-3.486938	10.858144
H	11.883268	-1.806021	10.877441
H	10.843609	-3.106522	10.261694
C	16.557589	-4.995755	12.842493
H	16.931850	-5.461288	11.926617
H	15.507454	-5.261744	12.962679
C	17.375764	-5.409707	14.048894
C	18.633638	-4.843740	14.289793
H	19.025096	-4.093660	13.607883
C	19.379926	-5.235383	15.400726
H	20.352994	-4.786868	15.581132
C	18.877522	-6.198548	16.280427
H	19.458006	-6.500089	17.147838
C	17.626193	-6.769299	16.041841
H	17.227141	-7.515341	16.723431
C	16.876310	-6.371824	14.932458
H	15.896862	-6.809187	14.753756
H	15.561022	0.114914	14.876646

GS2 Entry D major solvent=CH_2Cl_2: The ground state structure (GS2) of the major product obtained from the click reaction between ABSACN Entry D and BnN_3 with CH_2Cl_2 as the solvent. (Table 4, Table S50)

S	13.835873	-1.342590	15.116622
O	14.840086	-0.991268	16.126319
O	12.409168	-1.346944	15.460906
N	14.167880	-2.911068	14.653494
N	16.468556	0.018929	13.857163
N	16.527180	-2.045612	11.033272
N	15.772396	-3.117525	10.704846
N	15.385054	-3.681421	11.821616
C	13.992839	-0.217432	13.704099
C	15.209086	0.336633	13.261403
C	15.191923	1.198431	12.160258
H	16.128887	1.642791	11.837277
C	14.002179	1.500475	11.499057
H	14.015464	2.172466	10.646535
C	12.802933	0.943810	11.941667
H	11.869483	1.173511	11.437785
C	12.798556	0.093121	13.045273

H 11.870570 -0.331220 13.408951
C 15.543378 -3.307670 14.289615
H 15.605947 -4.386175 14.458811
H 16.220348 -2.820622 14.993854
C 15.896348 -2.981579 12.874403
C 16.643902 -1.927623 12.383304
C 17.386503 -0.844116 13.090081
H 18.095219 -1.263552 13.806804
H 17.974924 -0.252959 12.379208
H 13.452289 -3.262787 14.018379
C 17.006443 0.736474 14.912691
C 17.123261 -1.228048 9.979025
H 16.663326 -1.581979 9.052754
H 16.812841 -0.189748 10.126594
C 18.631752 -1.344839 9.936354
C 19.249728 -2.595911 10.052534
C 19.418392 -0.202781 9.759189
C 20.809280 -0.308397 9.689005
C 20.638243 -2.700803 9.988831
C 21.421384 -1.557514 9.804825
H 18.944519 0.772728 9.677199
H 21.411316 0.585493 9.552501
H 18.642110 -3.485610 10.195236
H 21.109579 -3.675285 10.081453
H 22.503513 -1.640004 9.756927
O 18.149220 0.508451 15.299473
C 16.130352 1.801563 15.534185
H 15.773594 2.512858 14.782641
H 15.260096 1.343206 16.009038
H 16.719974 2.328917 16.285159

GS2 Entry D minor solvent=CH_2Cl_2: The ground state structure (GS2) of the minor product obtained from the click reaction between ABSACN Entry D and BnN_3 with CH_2Cl_2 as the solvent. (Table 4, Table S51)

S 13.513267 -1.118551 15.516949
O 14.186215 -0.328380 16.552485
O 12.150425 -1.618246 15.732519
N 14.431241 -2.500003 15.314211
N 15.723319 0.834717 14.258631
N 17.054935 -1.378552 11.803561
N 16.816694 -2.653089 11.613375
N 16.383089 -3.163376 12.785266
C 13.466262 -0.178426 13.973081
C 14.509287 0.655727 13.525665
C 14.366453 1.299775 12.293727
H 15.171208 1.941293 11.947912
C 13.217368 1.133686 11.522294
H 13.131824 1.647154 10.569556
C 12.183243 0.318618 11.981005
H 11.279963 0.189572 11.393267
C 12.307217 -0.334061 13.205771
H 11.509127 -0.965280 13.577056
C 15.893257 -2.428704 15.157195
H 16.302144 -3.360858 15.556720
H 16.246205 -1.617927 15.796344
C 16.355491 -2.205354 13.752978
C 16.788050 -1.063898 13.103385
C 16.962008 0.316048 13.643582
H 17.723979 0.351515 14.424117
H 17.294766 0.968711 12.827545
H 13.948299 -3.173937 14.723992
C 16.074506 -4.589609 12.891224
H 16.141119 -4.974100 11.870195
H 15.036101 -4.706583 13.216939
C 17.019113 -5.311877 13.827450
C 18.404158 -5.177153 13.670544
H 18.796128 -4.546094 12.877033
C 19.274728 -5.843492 14.531111
H 20.348088 -5.734217 14.403491
C 18.768829 -6.650142 15.555340
H 19.449035 -7.165452 16.227435

C	17.389587	-6.787406	15.714875
H	16.990121	-7.408823	16.511198
C	16.516981	-6.116850	14.853960
H	15.442029	-6.220965	14.982673
C	15.857685	1.810454	15.237597
O	16.949395	2.033223	15.753152
C	14.616741	2.598015	15.598747
H	14.883440	3.305145	16.385431
H	14.245950	3.148817	14.727241
H	13.819777	1.942044	15.951574
